# Dual energy X-ray absorptiometry body composition reference values of limbs and trunk from NHANES 1999–2004 with additional visualization methods

**DOI:** 10.1371/journal.pone.0174180

**Published:** 2017-03-27

**Authors:** Benjamin J. Hinton, Bo Fan, Bennett K. Ng, John A. Shepherd

**Affiliations:** 1 Department of Radiology & Biomedical Imaging, University of California—San Francisco, San Francisco, California, United States of America; 2 Department of Bioengineering, University of California Berkeley and University of California San Francisco, San Francisco, California, United States of America; Montclair State University, UNITED STATES

## Abstract

Body Mass Index has traditionally been used as a measure of health, but Fat Mass Index (FMI) and Lean Mass Index (LMI) have been shown to be more predictive of mortality and health risk. Total body FMI and LMI reference curves have particularly been useful in quantifying sarcopenia and sarcopenic obesity. Research has shown regional composition has significant associations to health outcomes. We derived FMI and LMI reference curves of the regions of the body (leg, arm, and trunk) for 15,908 individuals in the 1999–2004 National Health and Nutrition Examination Survey data for each sex and ethnicity using the Lambda-Mu-Sigma (LMS) method and developed software to visualize this regional composition. These reference curves displayed differentiation between males and females during puberty and sharper limb LMI declines during late adulthood for males. For adults ages 30–50, females had 39%, 83%, and 47% larger arm, leg, and trunk FMI values than males, respectively. Males had 49%, 20%, and 15% higher regional LMI values than females for the arms, legs, and trunk respectively. The leg FMI and LMI of black females were 14% and 15% higher respectively than those of Hispanic and white females. White and Hispanic males had 37% higher trunk FMI values than black males. Hispanic females had 20% higher trunk FMI than white and black females. These data underscore the importance of accounting for sex and ethnicity in studies of regional composition. This study is the first to produce regional LMI and FMI reference tables and curves from the NHANES dataset. These reference curves provide a framework useful in studies and research involving sarcopenia, obesity, sarcopenic obesity, and other studies of compositional phenotypes. Further, the software tool we provide for visualizing regional composition will prove useful in monitoring progress in physical therapy, diets, or other attempts to attain healthier compositions.

## Introduction

Body composition is a known risk factor for a number of conditions such as diabetes and heart disease that contribute to higher healthcare costs and reduced lifespan [1,2]. Body mass index (BMI, total mass/height^2^) and waist circumference have long been used as indicators of body shape and adiposity and as crude measures of health risk [3,4], but these measures are not specific to lean or fat mass. Fat Mass Index (FMI, fat mass/height^2^) and Lean Mass Index (LMI, lean mass/height^2^) have been introduced as more specific composition measures than BMI [5–8], but even these measures are not specific to the composition of each region (arms, legs, trunk) of the body.

In many studies regional fat mass and composition has been shown to be predictive of cardiovascular disease, regional lipolysis, blood pressure, and other conditions. [9–16]. Wilson et al. showed that the volume ratio of trunk to leg had a strong association to diabetes and mortality that was independent of total fat distribution [17]. Prado et al. used regional composition of the limbs to calculate Appendicular Lean Mass Index (ALMI) and proposed new body shape and composition phenotypes to study along with ways to diagnose sarcopenia and sarcopenic obesity [18]. Regional composition and volume measurements play an important role in both direct associations to disease states and in developing an improved understanding of healthy compositional phenotypes.

Performing studies with standardized reference curves of regional composition provides advantages over using raw regional FMI and LMI values. First, reference curves inherently control for differences in sex, age, and ethnicity [19]. Second, Z-scores and T-scores are more interpretable than raw FMI and LMI values or ratios in many cases. Lastly, conditions such as sarcopenia and sarcopenic obesity rely on Z-score or T-score cutoff values for diagnosis [20–22]. Reference curves have been generated using the LMS method for total BMI, FMI, and LMI [3,23–25], but as of yet no reference curves have been produced for regional fat and lean composition of the U.S. population. Deriving such reference curves would prove useful for groups studying how regional body composition varies across demographic groups and how it affects different health outcomes.

In this study, we produced FMI and LMI reference curves and LMS tables for the legs, arms, and trunk by sex and ethnicity in a representative U.S. sample. These LMS tables will allow researchers to determine when individuals have higher or lower fat or lean mass in different regions of the body for a given age, sex, and ethnicity by calculating Z-scores in each of those regions. We further produced software to visualize an individual’s regional distribution of FMI and LMI Z-scores using radar charts. We do not aim to explain many of the differences found between demographics, but to provide this data as a useful tool for groups investigating the effects of regional distribution, body shape, and composition on metabolic conditions such as sarcopenia, sarcopenic obesity, and many other conditions.

## Subjects and methods

Our study aimed to produce regional reference values for FMI and LMI of the arm, leg, and trunk for by sex and ethnicity in the cross sectional dual-energy X-ray (DXA) measurements from the 1999–2004 National Health and Nutrition Examination Survey (NHANES). NHANES uses a rigorous sampling method and has been used many times to provide an accurate representative sample of descriptive health statistics of the U.S. population [25,26].

### Subjects

NHANES DXA scans report whole body and regional measures of fat mass, lean mass, bone mineral content, and bone mineral density [25]. Measurements for our study were taken from 15,908 individuals from the NHANES reference database from 1999–2004 for all individuals aged 8–85 [25].

This survey used a multistage sampling method to enroll individuals in the study. Because reference compositional values are unique by ethnicity, the survey provides representative statistics for different self-reported U.S. ethnic groups (non-Hispanic whites, non-Hispanic blacks, Mexican Americans, other Hispanics, and other minorities) [25–27]. In order to provide more reliable estimates, blacks, Mexican Americans, low-income whites, individuals between 12–19 years old and above 60 years old were oversampled [25]. Subjects were excluded if they were above the weight (136 kg) or height (196 cm) limit of the DXA table. Females were excluded if they reported they were pregnant or if a pregnancy test was positive at exam time [25]. Approval for the study was obtained from the National Center for Health Statistics international review board.

### DXA measurement protocol

Our analysis used the DXA data sets released by NHANES from 1999–2004 without imputation on the Center for Disease Control website (http://www.cdc.gov/nchs/about/major/nhanes/dxx/dxa.htm). DXA scans in NHANES were acquired per manufacturer recommendations of the QDR 4500A fan beam densitometer (Hologic, Inc., Bedford, MA). All subjects wore paper gowns and removed jewelry and other personal items capable of interfering with the DXA exam. These exams were reviewed and analyzed by the University of California-San Francisco Department of Radiology Bone Density Group. Prosthetics, implants and other regional devices capable of affecting results were listed as missing in the dataset and not included in our analysis [25].

Body composition results are calibration dependent and results provided by different instruments can vary. In 1999–2004 NHANES, the DXA scans were analyzed using the Hologic Discovery software version 12.1. NHANES calibration from Schoeller et al [28] were applied before results publicly released. The NHANES data sets contained whole body bone mineral content, bone mineral density, percent fat, lean mass, fat mass as well as with regional measurements (each arm and leg along with trunk) [25].

### Producing reference curves

From the DXA measures, we calculated the FMI and LMI for the trunk, average arm, and average leg by dividing fat and lean mass of each region by the square of height [24,26,29]. Next, we calculated the reference curves of these regional FMI and LMI values using a LMS curve fitting method (lmsChartMaker Pro Version 2.54) [30,31]. LMS is a mathematical method to produce reference curves for measures that corrects for skewed data by generating an “L” (power), “M” (Median), and “S” (Coefficient of Variation) curve across ages of interest. It has been used in the past to calculate reference curves and centiles for height, BMI, and total FMI and LMI [26,31–33]. This method produces Z-scores via the following equation [19]:

$$z=\frac{{\left[\frac{y}{M\left(t\right)}\right]}^{L\left(t\right)}-1}{L\left(t\right)S\left(t\right)}$$ (1)

The centile curves of y (measure of interest) for a given t (age) are modeled by:

$$C_{100\alpha }\left(t\right)=M\left(t\right){\left(1+L\left(t\right)S\left(t\right)Z_{\alpha }\right)}^{1/L\left(t\right)}$$ (2)

We developed these reference curves and LMS tables for the three major self-reported U.S. ethnic groups from NHANES: non-Hispanic whites, non-Hispanic blacks, and Mexican Americans/other Hispanics (hereafter referred to as Hispanic). Mexican Americans and other Hispanics were grouped to increase power of the model. There were not enough observations to develop reference data for the other ethnic minorities group.

The degrees of freedom of the model were increased for each LMS parameter in the order suggested by the developers of LMS [19], and were only increased if it improved the Bayesian Information Criterion more than ln(N) units (N = Sample Size of demographic group), as done in other work to prevent overfitting [29]. As recommended by the LMS developers, we examined de-trended Q-Q plots and the fitted curves for smoothness of fit [30].

We used Eq 1 to apply the LMS values for each individual based on their demographic and their FMI and LMI data to produce Z-scores for every limb and the trunk. We applied the LMS values and from the average arm and leg to the left and right limbs to produce Z-scores for each of the four limbs, which allowed us to compare symmetry of the left and right appendages of the body. These Z-scores can then be used to determine if an individual has high or low fat or lean mass in different regions of the body for their respective age, sex, and ethnicity.

### Radar charts

To visualize regional differences, we created software that outputs a pentagonal radar chart of regional body composition, where each spoke represents the Z-score FMI and LMI values of each region (each leg, each arm, and trunk) of the body. These radar charts were produced in R (Version 3.2.3) with the fmsb and shiny packages. We opted to plot the Z-score of FMI and LMI for each appendage as opposed to an absolute value because it provided better scaled images and provided more information about regional composition relative to people of the same age/sex/ethnicity.

## Results

The number of observations used in the reference database by age group, sex, and ethnicity is provided in Table 1. These data show the distribution of participants across a wide age range and set of ethnicities and an adequate number of individuals across the age distribution for each sex and ethnicity except for the oldest nonwhite individuals.

**Table 1:** Number of observations in the NHANES reference database.

Age Group	Sex	Whites	Blacks	Hispanic
8 to 9	Male	128	162	197
	Female	67	92	75
10 to 11	Male	132	169	166
	Female	52	63	66
12 to 13	Male	205	269	331
	Female	149	177	199
14 to 15	Male	197	244	284
	Female	144	153	187
16 to 17	Male	208	271	316
	Female	145	129	147
18 to 19	Male	188	212	276
	Female	166	163	257
20 to 24	Male	191	105	162
	Female	186	78	155
25 to 29	Male	202	74	160
	Female	165	64	115
30 to 34	Male	202	88	132
	Female	198	81	98
35 to 39	Male	199	85	133
	Female	204	81	115
40 to 44	Male	220	109	152
	Female	199	99	161
45 to 49	Male	186	97	125
	Female	196	96	128
50 to 54	Male	223	79	81
	Female	224	60	98
55 to 59	Male	158	44	64
	Female	140	47	56
60 to 64	Male	185	68	133
	Female	185	87	150
65 to 69	Male	178	67	107
	Female	179	59	119
70 to 74	Male	198	47	88
	Female	168	38	91
75 to 79	Male	149	30	56
	Female	127	36	40
80 to 84	Male	159	12	27
	Female	170	17	25
85+	Male	75	10	10
	Female	86	13	18
Total	Male	3583	2242	3000
	Female	3150	1633	2300
		6733	3875	5300

We created reference curves and tables of LMS values and included them as supplemental figures and tables. A list of the reference curves and tables is provided in Table 2. For completeness, the tables for total FMI and total LMI were included. These centile curves show smooth transitions throughout the age range. De-trended Q-Q plots of the data affirmed the goodness of fit and our inclusion criterion for allowing extra degrees of freedom reduced overfitting. As expected, average Z-scores were very close to zero with standard deviations very close to one for all the fitted regional DXA measures.

**Table 2:** List of Reference curves and tables generated from NHANES DXA data.

DXA Measure	Supplemental Figure	Supplemental Tables (Female, Male)
Average Arm FMI	S1 Fig	S1 & S2 Tables (Black), S17 & S18 Tables (Hispanic), S33 & S34 Tables (White)
Average Arm LMI	S2 Fig	S3 & S4 Tables (Black), S19 & S20 Tables (Hispanic), S35 & S36 Tables (White)
Average Leg FMI	S3 Fig	S5 & S6 Tables (Black), S21 & S22 Tables (Hispanic), S37 & S38 Tables (White)
Average Leg LMI	S4 Fig	S7 & S8 Tables (Black), S23 & S24 Tables (Hispanic), S39 & S40 Tables (White)
Trunk FMI	S5 Fig	S9 & S10 Tables (Black), S25 & S26 Tables (Hispanic), S41 & S42 Tables (White)
Trunk LMI	S6 Fig	S11 & S12 Tables (Black), S27 & S28 Tables (Hispanic), S43 & S44 Tables (White)
Total FMI	S7 Fig	S13 & S14 Tables (Black), S29 & S30 Tables (Hispanic), S45 & S46 Tables (White)
Total LMI	S8 Fig	S15 & S16 Tables (Black), S31 & S32 Tables (Hispanic), S47 & S48 Tables (White)

For each DXA measure in column 1, male and female reference curves for white, black, and Hispanic subjects were modeled against age. Ages ranged from 8–85 years.

**Fig 1:**
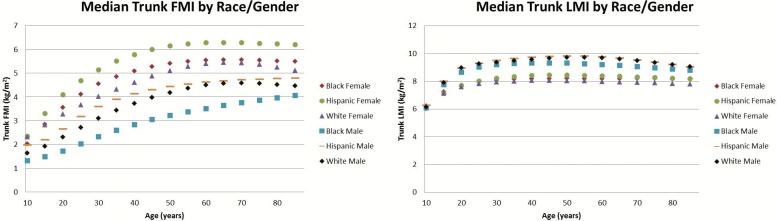
Median Trunk FMI and LMI values by ethnicity and sex. This comparison of the median trunk FMI values by ethnicity and sex (left) and median LMI values by ethnicity and sex (right). Females generally have larger trunk FMI and lower trunk LMI values than males, and males have a more pronounced drop off in trunk LMI values as they age compared to females. Deviations of each median measure not shown for figure clarity; consult S1–S8 Figs to examine individual data points with percentiles shown or the LMS tables to further examine coefficients of variation

**Fig 2:**
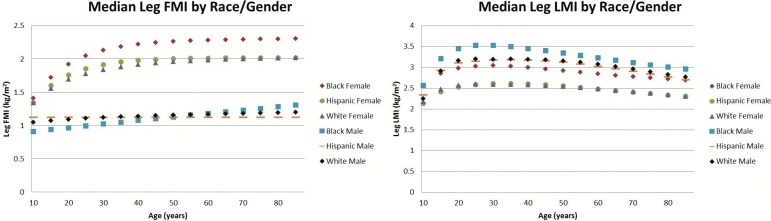
Median Leg FMI and LMI values by ethnicity and sex. This comparison of the median leg FMI values by ethnicity and sex (left) and median LMI values by ethnicity and sex (right). Females generally have larger leg FMI and lower leg LMI values than males, and black females tended to have larger FMI and LMI values in the legs compared to females of other ethnicities. Deviations of each median measure not shown for figure clarity; consult S1–S8 Figs to examine individual data points with percentiles shown or the LMS tables to further examine coefficients of variation

**Fig 3:**
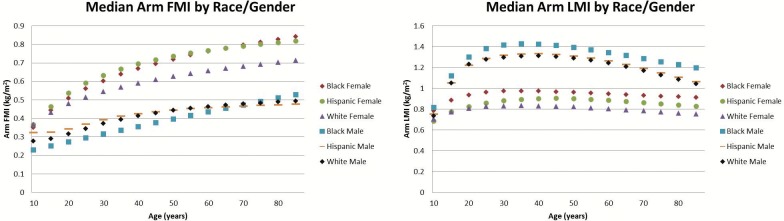
Median Arm FMI and LMI values by ethnicity and sex. This comparison of the median arm FMI values by ethnicity and sex (left) and median LMI values by ethnicity and sex (right). Females generally have larger arm FMI and lower arm LMI values than males. Males have a more pronounced drop off in their arm LMI values as they age compared to females. Deviations of each median measure not shown for figure clarity; consult S1–S8 Figs to examine individual data points with percentiles shown or the LMS tables to further examine coefficients of variation

There were noticeable differences observed across sex for the various measures, many of which varied with age. To help visualize some of these differences, we plotted the median (M) values across sex and ethnicity for the LMI and FMI of the trunk (Fig 1), the average leg (Fig 2), and the average arm (Fig 3). First, we noticed that in most cases and especially for regional LMI, differentiation occurred between males and females during the years of puberty and young adult development. Further in adults between 30 and 50, females had 39%, 83%, and 47% larger median arm, leg, and trunk FMI values than males. Males in this age range had 49%, 20%, and 15% higher regional LMI values than females for the arms, legs, and trunk respectively. Male median LMI values peaked in adulthood and decreased thereafter especially in limbs, while female median LMI values peaked in adulthood and did not experience as much of a decrease as male LMI values going into old age in the arm and trunk.

In this adult range of 30–50 years of age, there were also apparent differences in regional composition across ethnicity. The leg FMI and LMI of black females were 14% and 15% higher respectively than for Hispanic and white females. White and Hispanic males had 37% higher trunk FMI values than black males, while black males averaged 9% higher leg LMI than white and Hispanic males. Hispanic females had 20% higher trunk FMI than white and black females. Lastly, black and Hispanic females on average had 15% higher arm FMI than white females.

### Radar charts

We developed software to produce radar charts of regional FMI and LMI based on the age, sex, and ethnicity of an individual and the regional fat and lean mass values. The software selects the appropriate LMS table based on demographic information and calculates and displays Z-scores based on the fat and lean mass entries. An example output of the software which displays demographic information, composition information, and the radar chart is included in S1 File and an operating version of the software will be run on the Shepherd lab website (https://radiology.ucsf.edu/research/labs/breast-bone-density/resources) and will be free for anyone to use to further their research. The software is protected by UCSF copyright but is available free of charge for non-commercial use.

**Fig 4:**
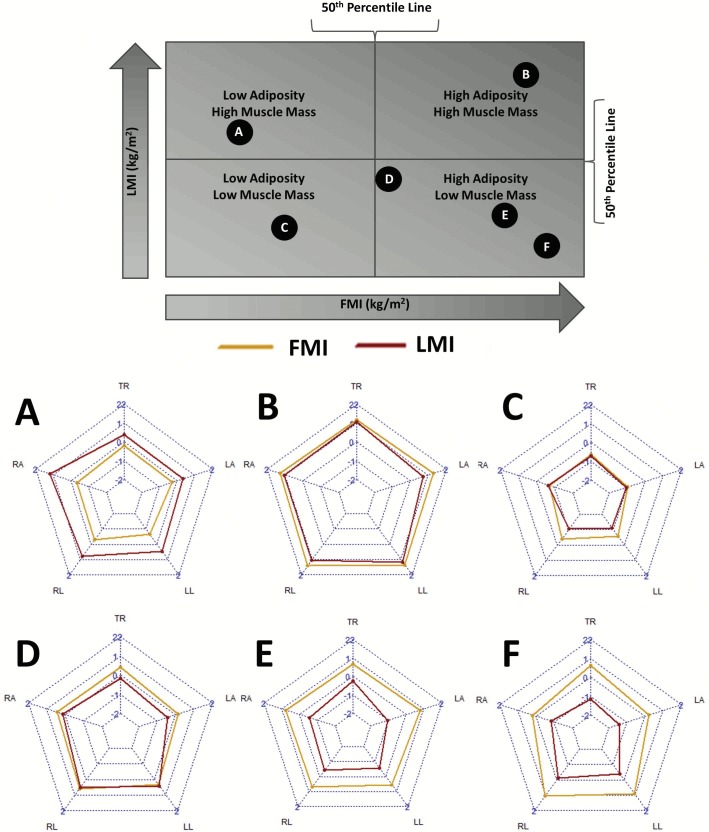
Sample radar charts of individuals in different quartiles of lean and fat mass indices. Radar charts of individuals as they fit into quadrants of adiposity and muscle mass. Each labeled circle in the above chart corresponds to an individual radar composition chart below. In the radar charts, each spoke represents: TR = Trunk, LA = Left Arm, LL = Left Leg, RL = Right Leg, RA = Right Arm.

Fig 4 shows several of the generated radar charts (plots A-F) for 6 individuals and charts those same individuals on a scale of percentile total LMI vs. percentile total FMI (top chart) to show what the generated radar charts look like for individuals of varying overall levels of lean mass and fat mass. Below average LMI individuals are at the bottom half of this chart and low FMI individuals are at the left half of this chart. This top chart, inspired the chart produced in work from Prado et al. to identify compositional categories of individuals [18], shows that the 6 individuals chosen represent a wide variation of overall LMI and FMI.

Radar charts A-F in Fig 4 shows the radar charts that display the FMI and LMI Z-scores of subjects A-F that were plotted in the above chart. The top spoke represents the trunk, the lower spokes represent the legs, and the middle spokes represent the arms. An individual with median regional FMI and LMI values (Z-scores of zero) would have two regular pentagons with every spoke at zero. Subject A in Fig 4 shows a high lean mass-low adiposity individual with more lean mass in the right half of their body. Subject B in Fig 4 shows an individual with high lean mass and high adiposity, and their radar chart reflects this with all values regional FMI and LMI Z-scores being above zero. Subject C in Fig 4 shows a sarcopenic individual in the low muscle mass low adiposity category. The second row of radar charts in Fig 4 depicts three levels of severity in the high adiposity low muscle mass category similar to those defined by Prado et al [18]. This high FMI and low LMI quadrant of the top chart contains many high risk groups including those with sarcopenic obesity. Subject D in Fig 4 shows someone with slightly higher than normal adiposity and slightly lower than normal muscle mass. Subject E shows an individual deeper in this high-risk quadrant of the top chart with above average adiposity and below average muscle mass. Subject F shows an individual severely in this high-risk quadrant of the top chart with much higher than normal adiposity and very low muscle mass relative to that.

We discovered a wide variety of different compositional shapes. We saw more asymmetry in the LMI Z-score distributions across regions than in the FMI Z-score distributions. Further, we found some individuals with distinct distributions, such as individuals who had relatively normal compositions in most regions but their legs, trunk, or arms contained more mass leading to a ‘spike’ in those regions in the radar chart.

## Discussion

This study is the first to produce regional LMI and FMI curves and LMS tables representative of the US population, which will be useful in many body composition studies [29]. This development of standard FMI and LMI LMS curves for each appendage and a method such as radar charts to visualize body symmetry will prove useful for doctors, researchers, therapists, athletes, and trainers.

These reference curves will help researchers that aim to investigate why differences exist between certain groups or groups that identify and monitor abnormal regional body composition patterns that arise in childhood and adulthood including sarcopenia, cachexia, anorexia nervosa, female athlete triad, growth hormone deficiencies, cancers, endocrine disturbances, and many others [20,26]. It has been shown that several regional compositional values are linked with different health outcomes. Sood et al. showed that trunk lean mass could be predictive of asthma in females [34]. Another study showed that two weeks of inactivity specifically reduced the lean mass of the legs in older adults [10]. Leg lean mass has been shown to be a predictor of femur BMD [35]. Studies of cardiovascular health have shown that trunk fat mass is a risk factor of cardiovascular disease and leg fat mass had a protective effect [14,16]. Studies have also shown that regional fat distribution affects the regional rate of lipolysis in obesity [15]. It is clear that regional body composition can affect various health outcomes and is worthy of studying, and this research will help to perform studies on height-normalized regional FMI and LMI values to better understand the role composition plays in these conditions.

This work also enables identification and monitoring of the relative symmetry and asymmetry of the lean and fat mass of individuals, as well as research on the effects of symmetry on the body. We noticed several cases of handedness, where a dominant leg or arm had more lean mass than the other, as observed in other studies [36,37]. Research has already shown limb and body symmetry plays a role in sports performance and injury prevention [38,39], and these tables and this software enables further research in the role regional symmetry plays in health and performance.

Analyzing the regional FMI and LMI median values highlights several trends that provide insight or warrant further investigation. We can see the clear effect of puberty in all regional LMI values, where males and females start out at similar values until adult development occurs. Once adult development occurs, we can see males have larger LMI values in every region while females have larger FMI values in every region. More research would have to be done to explain specifically why these differences occur, but these results align with previous comparisons of total body composition by gender and could partially be explained by endocrine differences [26,40,41]. It is interesting to note the differences are most pronounced in the limbs.

Further, we can see in some cases certain ethnicities have a different trend from other ethnicities of the same sex. Black males had lower trunk FMI and higher leg LMI than their white or Hispanic counterparts. Hispanic females averaged a noticeably higher trunk FMI than black or white females, and white females had lower arm FMI values than black or Hispanic females. These differences in regional composition by sex and ethnicity could serve as avenues of future research for some investigators and highlight the importance in accounting for sex and ethnicity in future body composition studies.

The creation of the software to create radar charts that visualize regional composition will be useful for researchers to intuitively interpret these data and any future studies of regional composition. These charts could aid in interpreting regional composition and in tracking changes over time through interventions such as diet, exercise, or other means. While the radar charts provide a mostly qualitative sense of composition, they provide an excellent structure to start visualizing these data and examining abnormalities, asymmetries, and changes over time.

This paper has several strengths that contribute to the power of the study. First, the large sample size from the NHANES data set provides a wide and comprehensive variety of data that describes the U.S. population by sex and ethnicity. Next, we have used established methods in producing these regional FMI and LMI values and LMS curves and our total body FMI/LMI LMS measurements matched up well with previous studies. Lastly, providing the software to create radar charts will make studies by other researchers much more accessible.

While there are several strengths to this study there are several limitations that, if avoided, would improve the study. A larger sample size especially in the black and Hispanic groups would have allowed for even more accurate reference curves especially at the ends of the age spectrum. Further segmentation of our population into separate LMS curves for youth and adults may have provided slightly improved curves, but this would have caused a sharp transition in Z scores during this transition. Further, our large sample size in this transition period produced LMS curves and de-trended Q-Q plots with enough smoothness to warrant calculating curves for all ages combined. Further, it should be noted that the values reports are only valid to directly compare in new measurements that use the same procedure and same machines as the NHANES dataset. Comparisons of these values to those derived on machines from different manufacturers could only be done after a cross-calibration process, as previously described for other NHANES DXA data [42,43]. Further studies will need to be performed in order to elucidate the usefulness of these regional values and how to best use them in conjunction with full body composition measures for risk assessment.

From this study, we can conclude that these regional measures follow expected curves and already provide insight about compositional phenotypes by sex and ethnicity. Additionally, these data could be useful for stronger descriptions of risk of mortality and metabolic conditions. Implementing radar charts to visualize regional composition may enable patients to track their regional composition to avoid unhealthy or undesirable compositional shapes (e.g. larger fat mass centile than lean mass centile, larger trunk FMI centile than leg/arm FMI centile). In the future, we plan studies to further investigate the role that regional body composition plays in health outcomes.

## Supporting information

S1 Fig Centiles for Trunk Fat Mass/Height^2^ (kg/m^2^) vs. Age in individuals 8–85. In order from bottom to top, each line represents the 10^th^/25^th^/50^th^/75^th^/90^th^ percentile.
(TIFF)

S2 Fig Centiles for Trunk Lean Mass/Height^2^ (kg/m^2^) vs. Age in individuals 8–85. In order from bottom to top, each line represents the 10^th^/25^th^/50^th^/75^th^/90^th^ percentile.
(TIFF)

S3 Fig Centiles for Average Arm Fat Mass/Height^2^ (kg/m^2^) vs. Age in individuals 8–85. In order from bottom to top, each line represents the 10^th^/25^th^/50^th^/75^th^/90^th^ percentile.
(TIFF)

S4 Fig Centiles for Average Arm Lean Mass/Height^2^ (kg/m^2^) vs. Age in individuals 8–85. In order from bottom to top, each line represents the 10^th^/25^th^/50^th^/75^th^/90^th^ percentile.
(TIFF)

S5 Fig Centiles for Average Leg Fat Mass/Height^2^ (kg/m^2^) vs. Age in individuals 8–85. In order from bottom to top, each line represents the 10^th^/25^th^/50^th^/75^th^/90^th^ percentile.
(TIFF)

S6 Fig Centiles for Average Leg Lean Mass/Height^2^ (kg/m^2^) vs. Age in individuals 8–85. In order from bottom to top, each line represents the 10^th^/25^th^/50^th^/75^th^/90^th^ percentile.
(TIFF)

S7 Fig Centiles for Average Total Body Fat Mass/Height^2^ (kg/m^2^) vs. Age in individuals 8–85. In order from bottom to top, each line represents the 10^th^/25^th^/50^th^/75^th^/90^th^ percentile.
(TIFF)

S8 Fig Centiles for Average Total Body Lean Mass/Height^2^ (kg/m^2^) vs. Age in individuals 8–85. In order from bottom to top, each line represents the 10^th^/25^th^/50^th^/75^th^/90^th^ percentile.
(TIFF)

S1 Table LMS values for average arm FMI in black females for ages 8–85. This table provides L, M, and S values to derive average arm FMI Z-scores for 3^rd^ through 97^th^ percentiles for black females ages 8–85.
(DOCX)

S2 Table LMS values for average arm FMI in black males for ages 8–85. This table provides L, M, and S values to derive average arm FMI Z-scores for 3rd through 97th percentiles for black males ages 8–85.
(DOCX)

S3 Table LMS values for average arm LMI in black females for ages 8–85. This table provides L, M, and S values to derive average arm LMI Z-scores for 3rd through 97th percentiles for black females ages 8–85.
(DOCX)

S4 Table LMS values for average arm LMI in black males for ages 8–85. This table provides L, M, and S values to derive average arm LMI Z-scores for 3rd through 97th percentiles for black males ages 8–85.
(DOCX)

S5 Table LMS values for average leg FMI in black females for ages 8–85. This table provides L, M, and S values to derive average leg FMI Z-scores for 3rd through 97th percentiles for black females ages 8–85.
(DOCX)

S6 Table LMS values for average leg FMI in black males for ages 8–85. This table provides L, M, and S values to derive average leg FMI Z-scores for 3rd through 97th percentiles for black males ages 8–85.
(DOCX)

S7 Table LMS values for average leg LMI in black females for ages 8–85. This table provides L, M, and S values to derive average leg LMI Z-scores for 3rd through 97th percentiles for black males ages 8–85.
(DOCX)

S8 Table LMS values for average leg LMI in black males for ages 8–85. This table provides L, M, and S values to derive average leg LMI Z-scores for 3rd through 97th percentiles for black males ages 8–85.
(DOCX)

S9 Table LMS values for trunk FMI in black females for ages 8–85. This table provides L, M, and S values to derive trunk FMI Z-scores for 3rd through 97th percentiles for black females ages 8–85.
(DOCX)

S10 Table LMS values for trunk FMI in black males for ages 8–85. This table provides L, M, and S values to derive trunk FMI Z-scores for 3rd through 97th percentiles for black males ages 8–85.
(DOCX)

S11 Table LMS values for trunk LMI in black females for ages 8–85. This table provides L, M, and S values to derive trunk LMI Z-scores for 3rd through 97th percentiles for black females ages 8–85.
(DOCX)

S12 Table LMS values for trunk LMI in black males for ages 8–85. This table provides L, M, and S values to derive trunk LMI Z-scores for 3rd through 97th percentiles for black males ages 8–85.
(DOCX)

S13 Table LMS values for total body FMI in black females for ages 8–85. This table provides L, M, and S values to derive total body FMI Z-scores for 3rd through 97th percentiles for black females ages 8–85.
(DOCX)

S14 Table LMS values for total body FMI in black males for ages 8–85. This table provides L, M, and S values to derive total body FMI Z-scores for 3rd through 97th percentiles for black males ages 8–85.
(DOCX)

S15 Table LMS values for total body LMI in black females for ages 8–85. This table provides L, M, and S values to derive total body LMI Z-scores for 3rd through 97th percentiles for black females ages 8–85.
(DOCX)

S16 Table LMS values for total body LMI in black males for ages 8–85. This table provides L, M, and S values to derive total body LMI Z-scores for 3rd through 97th percentiles for black males ages 8–85.
(DOCX)

S17 Table LMS values for average arm FMI in Hispanic females for ages 8–85. This table provides L, M, and S values to derive average arm FMI Z-scores for 3^rd^ through 97^th^ percentiles for Hispanic females ages 8–85.
(DOCX)

S18 Table LMS values for average arm FMI in Hispanic males for ages 8–85. This table provides L, M, and S values to derive average arm FMI Z-scores for 3rd through 97th percentiles for Hispanic males ages 8–85.
(DOCX)

S19 Table LMS values for average arm LMI in Hispanic females for ages 8–85. This table provides L, M, and S values to derive average arm LMI Z-scores for 3rd through 97th percentiles for Hispanic females ages 8–85.
(DOCX)

S20 Table LMS values for average arm LMI in Hispanic males for ages 8–85. This table provides L, M, and S values to derive average arm LMI Z-scores for 3rd through 97th percentiles for Hispanic males ages 8–85.
(DOCX)

S21 Table LMS values for average leg FMI in Hispanic females for ages 8–85. This table provides L, M, and S values to derive average leg FMI Z-scores for 3rd through 97th percentiles for Hispanic females ages 8–85.
(DOCX)

S22 Table LMS values for average leg FMI in Hispanic males for ages 8–85. This table provides L, M, and S values to derive average leg FMI Z-scores for 3rd through 97th percentiles for Hispanic males ages 8–85.
(DOCX)

S23 Table LMS values for average leg LMI in Hispanic females for ages 8–85. This table provides L, M, and S values to derive average leg LMI Z-scores for 3rd through 97th percentiles for Hispanic males ages 8–85.
(DOCX)

S24 Table LMS values for average leg LMI in Hispanic males for ages 8–85. This table provides L, M, and S values to derive average leg LMI Z-scores for 3rd through 97th percentiles for Hispanic males ages 8–85.
(DOCX)

S25 Table LMS values for trunk FMI in Hispanic females for ages 8–85. This table provides L, M, and S values to derive trunk FMI Z-scores for 3rd through 97th percentiles for Hispanic females ages 8–85.
(DOCX)

S26 Table LMS values for trunk FMI in Hispanic males for ages 8–85. This table provides L, M, and S values to derive trunk FMI Z-scores for 3rd through 97th percentiles for Hispanic males ages 8–85.
(DOCX)

S27 Table LMS values for trunk LMI in Hispanic females for ages 8–85. This table provides L, M, and S values to derive trunk LMI Z-scores for 3rd through 97th percentiles for Hispanic females ages 8–85.
(DOCX)

S28 Table LMS values for trunk LMI in Hispanic males for ages 8–85. This table provides L, M, and S values to derive trunk LMI Z-scores for 3rd through 97th percentiles for Hispanic males ages 8–85.
(DOCX)

S29 Table LMS values for total body FMI in Hispanic females for ages 8–85. This table provides L, M, and S values to derive total body FMI Z-scores for 3rd through 97th percentiles for Hispanic females ages 8–85.
(DOCX)

S30 Table LMS values for total body FMI in Hispanic males for ages 8–85. This table provides L, M, and S values to derive total body FMI Z-scores for 3rd through 97th percentiles for Hispanic males ages 8–85.
(DOCX)

S31 Table LMS values for total body LMI in Hispanic females for ages 8–85. This table provides L, M, and S values to derive total body LMI Z-scores for 3rd through 97th percentiles for Hispanic females ages 8–85.
(DOCX)

S32 Table LMS values for total body LMI in Hispanic males for ages 8–85. This table provides L, M, and S values to derive total body LMI Z-scores for 3rd through 97th percentiles for Hispanic males ages 8–85.
(DOCX)

S33 Table LMS values for average arm FMI in white females for ages 8–85. This table provides L, M, and S values to derive average arm FMI Z-scores for 3^rd^ through 97^th^ percentiles for white females ages 8–85.
(DOCX)

S34 Table LMS values for average arm FMI in white males for ages 8–85. This table provides L, M, and S values to derive average arm FMI Z-scores for 3rd through 97th percentiles for white males ages 8–85.
(DOCX)

S35 Table LMS values for average arm LMI in white females for ages 8–85. This table provides L, M, and S values to derive average arm LMI Z-scores for 3rd through 97th percentiles for white females ages 8–85.
(DOCX)

S36 Table LMS values for average arm LMI in white males for ages 8–85. This table provides L, M, and S values to derive average arm LMI Z-scores for 3rd through 97th percentiles for white males ages 8–85.
(DOCX)

S37 Table LMS values for average leg FMI in white females for ages 8–85. This table provides L, M, and S values to derive average leg FMI Z-scores for 3rd through 97th percentiles for white females ages 8–85.
(DOCX)

S38 Table LMS values for average leg FMI in white males for ages 8–85. This table provides L, M, and S values to derive average leg FMI Z-scores for 3rd through 97th percentiles for white males ages 8–85.
(DOCX)

S39 Table LMS values for average leg LMI in white females for ages 8–85. This table provides L, M, and S values to derive average leg LMI Z-scores for 3rd through 97th percentiles for white males ages 8–85.
(DOCX)

S40 Table LMS values for average leg LMI in white males for ages 8–85. This table provides L, M, and S values to derive average leg LMI Z-scores for 3rd through 97th percentiles for white males ages 8–85.
(DOCX)

S41 Table LMS values for trunk FMI in white females for ages 8–85. This table provides L, M, and S values to derive trunk FMI Z-scores for 3rd through 97th percentiles for white females ages 8–85.
(DOCX)

S42 Table LMS values for trunk FMI in white males for ages 8–85. This table provides L, M, and S values to derive trunk FMI Z-scores for 3rd through 97th percentiles for white males ages 8–85.
(DOCX)

S43 Table LMS values for trunk LMI in white females for ages 8–85. This table provides L, M, and S values to derive trunk LMI Z-scores for 3rd through 97th percentiles for white females ages 8–85.
(DOCX)

S44 Table LMS values for trunk LMI in white males for ages 8–85. This table provides L, M, and S values to derive trunk LMI Z-scores for 3rd through 97th percentiles for white males ages 8–85.
(DOCX)

S45 Table LMS values for total body FMI in white females for ages 8–85. This table provides L, M, and S values to derive total body FMI Z-scores for 3rd through 97th percentiles for white females ages 8–85.
(DOCX)

S46 Table LMS values for total body FMI in white males for ages 8–85. This table provides L, M, and S values to derive total body FMI Z-scores for 3rd through 97th percentiles for white males ages 8–85.
(DOCX)

S47 Table LMS values for total body LMI in white females for ages 8–85. This table provides L, M, and S values to derive total body LMI Z-scores for 3rd through 97th percentiles for white females ages 8–85.
(DOCX)

S48 Table LMS values for total body LMI in white males for ages 8–85. This table provides L, M, and S values to derive total body LMI Z-scores for 3rd through 97th percentiles for white males ages 8–85.
(DOCX)

S1 File PDF file of a sample output from the radar chart generation software. (PDF)
