# Supplementary figures and images for: Dual energy X-ray absorptiometry body composition reference values of limbs and trunk from NHANES 1999–2004 with additional visualization methods
Source: PLoS One. 2017 Mar 27;12(3):e0174180. doi: 10.1371/journal.pone.0174180 (PMC5367711; doi:10.1371/journal.pone.0174180)

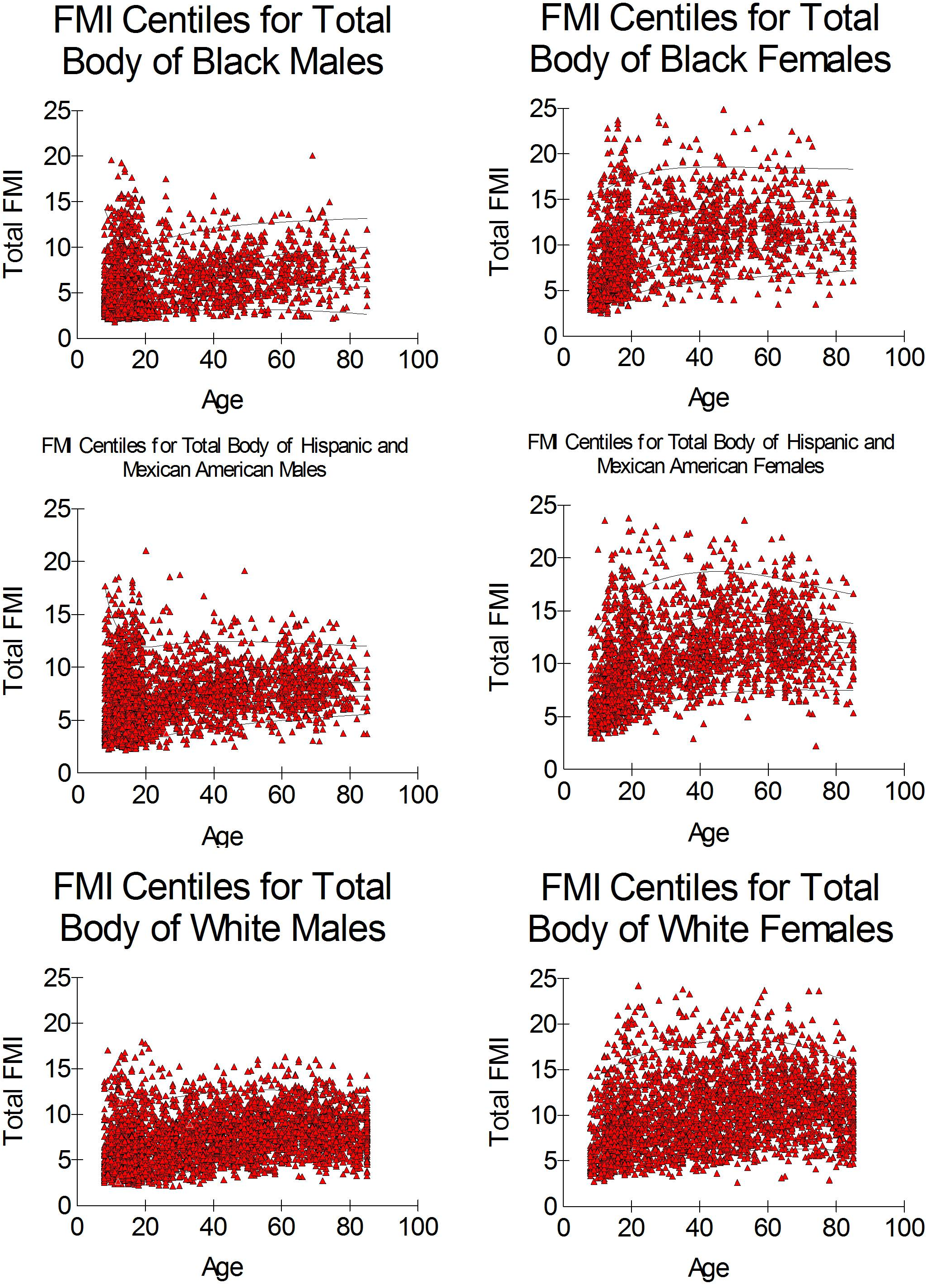

Supplement: S7 Fig — In order from bottom to top, each line represents the 10th/25th/50th/75th/90th percentile. (TIFF) [file pone.0174180.s007.tiff]
