# Supplementary material for: Dual energy X-ray absorptiometry body composition reference values of limbs and trunk from NHANES 1999–2004 with additional visualization methods
Source: PLoS One. 2017 Mar 27;12(3):e0174180. doi: 10.1371/journal.pone.0174180 (PMC5367711; doi:10.1371/journal.pone.0174180)
Supplement: S1 Table — This table provides L, M, and S values to derive average arm FMI Z-scores for 3rd through 97th percentiles for black females ages 8–85. (DOCX) [file pone.0174180.s009.docx]

Table S1: LMS Curve Fit Data providing L, M, and S values for 3^rd^ through 97^th^ percentiles for Black Females Ages 8-85 for Average Arm FMI.

|  | Females | | | | | | | | |
| --- | --- | --- | --- | --- | --- | --- | --- | --- | --- |
|  |  |  | M | | | | | | |
| Age | L | S | 3 | 5 | 25 | 50 | 75 | 95 | 97 |
| 8 | -0.449 | 0.567 | 0.125 | 0.138 | 0.211 | 0.300 | 0.455 | 1.002 | 1.277 |
| 10 | -0.346 | 0.544 | 0.146 | 0.161 | 0.248 | 0.351 | 0.519 | 1.023 | 1.239 |
| 12 | -0.263 | 0.525 | 0.163 | 0.180 | 0.280 | 0.393 | 0.569 | 1.045 | 1.230 |
| 14 | -0.192 | 0.509 | 0.178 | 0.197 | 0.307 | 0.428 | 0.610 | 1.065 | 1.232 |
| 16 | -0.130 | 0.495 | 0.190 | 0.211 | 0.331 | 0.458 | 0.645 | 1.084 | 1.237 |
| 18 | -0.076 | 0.483 | 0.202 | 0.224 | 0.352 | 0.485 | 0.675 | 1.102 | 1.244 |
| 20 | -0.028 | 0.472 | 0.212 | 0.236 | 0.371 | 0.510 | 0.701 | 1.117 | 1.252 |
| 25 | 0.075 | 0.449 | 0.234 | 0.262 | 0.413 | 0.561 | 0.756 | 1.151 | 1.272 |
| 30 | 0.159 | 0.430 | 0.253 | 0.284 | 0.448 | 0.602 | 0.800 | 1.179 | 1.290 |
| 35 | 0.230 | 0.415 | 0.270 | 0.304 | 0.478 | 0.638 | 0.836 | 1.202 | 1.307 |
| 40 | 0.291 | 0.401 | 0.285 | 0.321 | 0.504 | 0.668 | 0.867 | 1.222 | 1.321 |
| 45 | 0.345 | 0.389 | 0.299 | 0.338 | 0.528 | 0.695 | 0.894 | 1.239 | 1.335 |
| 50 | 0.394 | 0.378 | 0.313 | 0.353 | 0.550 | 0.720 | 0.917 | 1.255 | 1.346 |
| 55 | 0.438 | 0.368 | 0.325 | 0.367 | 0.570 | 0.741 | 0.938 | 1.268 | 1.357 |
| 60 | 0.478 | 0.359 | 0.337 | 0.380 | 0.589 | 0.761 | 0.957 | 1.281 | 1.367 |
| 65 | 0.515 | 0.351 | 0.348 | 0.393 | 0.606 | 0.780 | 0.975 | 1.292 | 1.376 |
| 70 | 0.549 | 0.343 | 0.359 | 0.405 | 0.622 | 0.797 | 0.990 | 1.303 | 1.384 |
| 75 | 0.580 | 0.336 | 0.370 | 0.417 | 0.637 | 0.812 | 1.005 | 1.312 | 1.392 |
| 80 | 0.610 | 0.329 | 0.380 | 0.428 | 0.652 | 0.827 | 1.019 | 1.321 | 1.399 |
| 85 | 0.638 | 0.323 | 0.390 | 0.439 | 0.665 | 0.841 | 1.031 | 1.329 | 1.406 |
|  |  |  |  |  |  |  |  |  |  |
