# Supplementary material for: Dual energy X-ray absorptiometry body composition reference values of limbs and trunk from NHANES 1999–2004 with additional visualization methods
Source: PLoS One. 2017 Mar 27;12(3):e0174180. doi: 10.1371/journal.pone.0174180 (PMC5367711; doi:10.1371/journal.pone.0174180)
Supplement: S2 Table — This table provides L, M, and S values to derive average arm FMI Z-scores for 3rd through 97th percentiles for black males ages 8–85. (DOCX) [file pone.0174180.s010.docx]

Table S2: LMS Curve Fit Data providing L, M, and S values for 3^rd^ through 97^th^ percentiles for Black Males Ages 8-85 for Average Arm FMI.

|  | Males | | | | | | | | |
| --- | --- | --- | --- | --- | --- | --- | --- | --- | --- |
|  |  |  | M | | | | | | |
| Age | L | S | 3 | 5 | 25 | 50 | 75 | 95 | 97 |
| 8 | -0.937 | 0.457 | 0.117 | 0.124 | 0.167 | 0.219 | 0.316 | 0.808 | 1.266 |
| 10 | -0.880 | 0.454 | 0.121 | 0.129 | 0.174 | 0.229 | 0.327 | 0.771 | 1.111 |
| 12 | -0.824 | 0.450 | 0.125 | 0.133 | 0.181 | 0.238 | 0.337 | 0.746 | 1.016 |
| 14 | -0.769 | 0.447 | 0.129 | 0.138 | 0.188 | 0.247 | 0.348 | 0.729 | 0.953 |
| 16 | -0.714 | 0.443 | 0.133 | 0.142 | 0.195 | 0.256 | 0.358 | 0.716 | 0.908 |
| 18 | -0.660 | 0.440 | 0.137 | 0.146 | 0.202 | 0.264 | 0.368 | 0.707 | 0.876 |
| 20 | -0.607 | 0.437 | 0.140 | 0.150 | 0.208 | 0.273 | 0.378 | 0.701 | 0.851 |
| 25 | -0.477 | 0.428 | 0.149 | 0.160 | 0.225 | 0.294 | 0.401 | 0.694 | 0.813 |
| 30 | -0.349 | 0.420 | 0.157 | 0.170 | 0.241 | 0.315 | 0.425 | 0.695 | 0.794 |
| 35 | -0.223 | 0.412 | 0.164 | 0.179 | 0.256 | 0.336 | 0.447 | 0.700 | 0.786 |
| 40 | -0.099 | 0.404 | 0.171 | 0.187 | 0.272 | 0.356 | 0.469 | 0.708 | 0.785 |
| 45 | 0.024 | 0.397 | 0.177 | 0.195 | 0.287 | 0.376 | 0.491 | 0.718 | 0.787 |
| 50 | 0.145 | 0.389 | 0.183 | 0.202 | 0.303 | 0.396 | 0.512 | 0.729 | 0.793 |
| 55 | 0.265 | 0.381 | 0.187 | 0.209 | 0.318 | 0.415 | 0.532 | 0.741 | 0.800 |
| 60 | 0.384 | 0.374 | 0.191 | 0.215 | 0.333 | 0.434 | 0.552 | 0.754 | 0.809 |
| 65 | 0.502 | 0.366 | 0.195 | 0.221 | 0.349 | 0.454 | 0.572 | 0.768 | 0.820 |
| 70 | 0.619 | 0.359 | 0.197 | 0.227 | 0.364 | 0.473 | 0.592 | 0.782 | 0.831 |
| 75 | 0.735 | 0.351 | 0.199 | 0.232 | 0.379 | 0.492 | 0.611 | 0.796 | 0.842 |
| 80 | 0.850 | 0.344 | 0.199 | 0.236 | 0.394 | 0.510 | 0.631 | 0.810 | 0.855 |
| 85 | 0.965 | 0.337 | 0.199 | 0.239 | 0.409 | 0.529 | 0.650 | 0.825 | 0.867 |
|  |  |  |  |  |  |  |  |  |  |
