# Supplementary material for: Dual energy X-ray absorptiometry body composition reference values of limbs and trunk from NHANES 1999–2004 with additional visualization methods
Source: PLoS One. 2017 Mar 27;12(3):e0174180. doi: 10.1371/journal.pone.0174180 (PMC5367711; doi:10.1371/journal.pone.0174180)
Supplement: S3 Table — This table provides L, M, and S values to derive average arm LMI Z-scores for 3rd through 97th percentiles for black females ages 8–85. (DOCX) [file pone.0174180.s011.docx]

Table S3: LMS Curve Fit Data providing L, M, and S values for 3^rd^ through 97^th^ percentiles for Black Females Ages 8-85 for Average Arm LMI.

|  | Females | | | | | | | | |
| --- | --- | --- | --- | --- | --- | --- | --- | --- | --- |
|  |  |  | M | | | | | | |
| Age | L | S | 3 | 5 | 25 | 50 | 75 | 95 | 97 |
| 8 | -0.483 | 0.163 | 0.541 | 0.559 | 0.647 | 0.720 | 0.806 | 0.959 | 1.003 |
| 10 | -0.483 | 0.163 | 0.589 | 0.609 | 0.704 | 0.784 | 0.878 | 1.045 | 1.092 |
| 12 | -0.483 | 0.163 | 0.627 | 0.648 | 0.750 | 0.834 | 0.934 | 1.112 | 1.162 |
| 14 | -0.483 | 0.163 | 0.655 | 0.678 | 0.784 | 0.872 | 0.976 | 1.162 | 1.215 |
| 16 | -0.483 | 0.163 | 0.676 | 0.699 | 0.808 | 0.900 | 1.007 | 1.199 | 1.253 |
| 18 | -0.483 | 0.163 | 0.691 | 0.715 | 0.827 | 0.920 | 1.030 | 1.226 | 1.282 |
| 20 | -0.483 | 0.163 | 0.703 | 0.727 | 0.841 | 0.936 | 1.048 | 1.247 | 1.304 |
| 25 | -0.483 | 0.163 | 0.722 | 0.747 | 0.863 | 0.961 | 1.076 | 1.280 | 1.339 |
| 30 | -0.483 | 0.163 | 0.730 | 0.755 | 0.873 | 0.972 | 1.088 | 1.295 | 1.354 |
| 35 | -0.483 | 0.163 | 0.732 | 0.757 | 0.876 | 0.974 | 1.091 | 1.298 | 1.358 |
| 40 | -0.483 | 0.163 | 0.731 | 0.756 | 0.874 | 0.972 | 1.088 | 1.296 | 1.355 |
| 45 | -0.483 | 0.163 | 0.727 | 0.752 | 0.869 | 0.968 | 1.083 | 1.289 | 1.348 |
| 50 | -0.483 | 0.163 | 0.722 | 0.747 | 0.864 | 0.961 | 1.076 | 1.281 | 1.339 |
| 55 | -0.483 | 0.163 | 0.717 | 0.742 | 0.858 | 0.954 | 1.068 | 1.272 | 1.330 |
| 60 | -0.483 | 0.163 | 0.712 | 0.736 | 0.851 | 0.947 | 1.061 | 1.262 | 1.320 |
| 65 | -0.483 | 0.163 | 0.706 | 0.731 | 0.845 | 0.940 | 1.052 | 1.253 | 1.310 |
| 70 | -0.483 | 0.163 | 0.701 | 0.725 | 0.838 | 0.933 | 1.044 | 1.243 | 1.300 |
| 75 | -0.483 | 0.163 | 0.695 | 0.719 | 0.832 | 0.926 | 1.036 | 1.233 | 1.290 |
| 80 | -0.483 | 0.163 | 0.690 | 0.714 | 0.825 | 0.919 | 1.028 | 1.224 | 1.280 |
| 85 | -0.483 | 0.163 | 0.685 | 0.709 | 0.819 | 0.912 | 1.021 | 1.215 | 1.271 |
|  |  |  |  |  |  |  |  |  |  |
