# Supplementary material for: Dual energy X-ray absorptiometry body composition reference values of limbs and trunk from NHANES 1999–2004 with additional visualization methods
Source: PLoS One. 2017 Mar 27;12(3):e0174180. doi: 10.1371/journal.pone.0174180 (PMC5367711; doi:10.1371/journal.pone.0174180)
Supplement: S4 Table — This table provides L, M, and S values to derive average arm LMI Z-scores for 3rd through 97th percentiles for black males ages 8–85. (DOCX) [file pone.0174180.s012.docx]

Table S4: LMS Curve Fit Data providing L, M, and S values for 3^rd^ through 97^th^ percentiles for Black Males Ages 8-85 for Average Arm LMI.

|  | Males | | | | | | | | |
| --- | --- | --- | --- | --- | --- | --- | --- | --- | --- |
|  |  |  | M | | | | | | |
| Age | L | S | 3 | 5 | 25 | 50 | 75 | 95 | 97 |
| 8 | -0.429 | 0.156 | 0.552 | 0.571 | 0.657 | 0.728 | 0.810 | 0.955 | 0.996 |
| 10 | -0.265 | 0.156 | 0.616 | 0.638 | 0.737 | 0.818 | 0.910 | 1.066 | 1.110 |
| 12 | -0.131 | 0.156 | 0.699 | 0.724 | 0.839 | 0.932 | 1.036 | 1.210 | 1.257 |
| 14 | -0.017 | 0.156 | 0.793 | 0.823 | 0.957 | 1.063 | 1.180 | 1.374 | 1.426 |
| 16 | 0.081 | 0.156 | 0.871 | 0.904 | 1.055 | 1.172 | 1.301 | 1.511 | 1.566 |
| 18 | 0.168 | 0.156 | 0.924 | 0.961 | 1.123 | 1.249 | 1.386 | 1.605 | 1.663 |
| 20 | 0.246 | 0.156 | 0.961 | 0.999 | 1.171 | 1.302 | 1.445 | 1.670 | 1.729 |
| 25 | 0.410 | 0.156 | 1.011 | 1.054 | 1.241 | 1.382 | 1.531 | 1.763 | 1.822 |
| 30 | 0.544 | 0.156 | 1.029 | 1.075 | 1.272 | 1.417 | 1.570 | 1.802 | 1.860 |
| 35 | 0.658 | 0.156 | 1.031 | 1.079 | 1.281 | 1.429 | 1.581 | 1.811 | 1.868 |
| 40 | 0.756 | 0.156 | 1.023 | 1.072 | 1.278 | 1.425 | 1.577 | 1.802 | 1.858 |
| 45 | 0.843 | 0.156 | 1.009 | 1.058 | 1.266 | 1.413 | 1.563 | 1.782 | 1.836 |
| 50 | 0.921 | 0.156 | 0.990 | 1.040 | 1.248 | 1.394 | 1.541 | 1.755 | 1.807 |
| 55 | 0.991 | 0.156 | 0.969 | 1.020 | 1.227 | 1.371 | 1.515 | 1.723 | 1.773 |
| 60 | 1.055 | 0.156 | 0.946 | 0.996 | 1.202 | 1.344 | 1.485 | 1.686 | 1.735 |
| 65 | 1.114 | 0.156 | 0.922 | 0.972 | 1.176 | 1.315 | 1.452 | 1.648 | 1.695 |
| 70 | 1.168 | 0.156 | 0.897 | 0.947 | 1.148 | 1.285 | 1.419 | 1.608 | 1.653 |
| 75 | 1.219 | 0.156 | 0.873 | 0.923 | 1.121 | 1.255 | 1.385 | 1.569 | 1.612 |
| 80 | 1.267 | 0.156 | 0.850 | 0.899 | 1.095 | 1.226 | 1.353 | 1.531 | 1.573 |
| 85 | 1.311 | 0.156 | 0.828 | 0.877 | 1.070 | 1.198 | 1.322 | 1.495 | 1.536 |
|  |  |  |  |  |  |  |  |  |  |
