# Supplementary material for: Dual energy X-ray absorptiometry body composition reference values of limbs and trunk from NHANES 1999–2004 with additional visualization methods
Source: PLoS One. 2017 Mar 27;12(3):e0174180. doi: 10.1371/journal.pone.0174180 (PMC5367711; doi:10.1371/journal.pone.0174180)
Supplement: S5 Table — This table provides L, M, and S values to derive average leg FMI Z-scores for 3rd through 97th percentiles for black females ages 8–85. (DOCX) [file pone.0174180.s013.docx]

Table S5: LMS Curve Fit Data providing L, M, and S values for 3^rd^ through 97^th^ percentiles for Black Females Ages 8-85 for Average Leg FMI.

|  | Females | | | | | | | | |
| --- | --- | --- | --- | --- | --- | --- | --- | --- | --- |
|  |  |  | M | | | | | | |
| Age | L | S | 3 | 5 | 25 | 50 | 75 | 95 | 97 |
| 8 | -0.273 | 0.426 | 0.596 | 0.648 | 0.933 | 1.230 | 1.660 | 2.680 | 3.041 |
| 10 | -0.190 | 0.416 | 0.679 | 0.740 | 1.071 | 1.407 | 1.876 | 2.927 | 3.280 |
| 12 | -0.123 | 0.407 | 0.745 | 0.814 | 1.183 | 1.549 | 2.048 | 3.116 | 3.462 |
| 14 | -0.066 | 0.400 | 0.801 | 0.876 | 1.277 | 1.668 | 2.189 | 3.266 | 3.606 |
| 16 | -0.017 | 0.393 | 0.847 | 0.928 | 1.356 | 1.766 | 2.304 | 3.385 | 3.719 |
| 18 | 0.027 | 0.388 | 0.885 | 0.972 | 1.422 | 1.849 | 2.399 | 3.479 | 3.806 |
| 20 | 0.066 | 0.383 | 0.917 | 1.008 | 1.478 | 1.918 | 2.477 | 3.553 | 3.874 |
| 25 | 0.148 | 0.372 | 0.977 | 1.077 | 1.585 | 2.046 | 2.617 | 3.676 | 3.982 |
| 30 | 0.216 | 0.363 | 1.017 | 1.123 | 1.656 | 2.130 | 2.704 | 3.737 | 4.029 |
| 35 | 0.273 | 0.356 | 1.043 | 1.154 | 1.704 | 2.184 | 2.756 | 3.760 | 4.040 |
| 40 | 0.322 | 0.350 | 1.060 | 1.175 | 1.737 | 2.220 | 2.786 | 3.762 | 4.030 |
| 45 | 0.365 | 0.344 | 1.072 | 1.190 | 1.761 | 2.243 | 2.803 | 3.752 | 4.010 |
| 50 | 0.404 | 0.339 | 1.081 | 1.201 | 1.778 | 2.259 | 2.811 | 3.736 | 3.984 |
| 55 | 0.440 | 0.334 | 1.087 | 1.210 | 1.791 | 2.271 | 2.815 | 3.716 | 3.957 |
| 60 | 0.472 | 0.330 | 1.093 | 1.216 | 1.801 | 2.279 | 2.816 | 3.696 | 3.929 |
| 65 | 0.501 | 0.326 | 1.097 | 1.222 | 1.810 | 2.285 | 2.815 | 3.676 | 3.902 |
| 70 | 0.529 | 0.323 | 1.101 | 1.228 | 1.817 | 2.290 | 2.814 | 3.657 | 3.878 |
| 75 | 0.554 | 0.320 | 1.104 | 1.232 | 1.824 | 2.294 | 2.812 | 3.639 | 3.855 |
| 80 | 0.578 | 0.317 | 1.108 | 1.237 | 1.830 | 2.298 | 2.810 | 3.623 | 3.833 |
| 85 | 0.600 | 0.314 | 1.111 | 1.241 | 1.836 | 2.302 | 2.809 | 3.608 | 3.814 |
|  |  |  |  |  |  |  |  |  |  |
