# Supplementary material for: Dual energy X-ray absorptiometry body composition reference values of limbs and trunk from NHANES 1999–2004 with additional visualization methods
Source: PLoS One. 2017 Mar 27;12(3):e0174180. doi: 10.1371/journal.pone.0174180 (PMC5367711; doi:10.1371/journal.pone.0174180)
Supplement: S6 Table — This table provides L, M, and S values to derive average leg FMI Z-scores for 3rd through 97th percentiles for black males ages 8–85. (DOCX) [file pone.0174180.s014.docx]

Table S6: LMS Curve Fit Data providing L, M, and S values for 3^rd^ through 97^th^ percentiles for Black Males Ages 8-85 for Average Leg FMI.

|  | Males | | | | | | | | |
| --- | --- | --- | --- | --- | --- | --- | --- | --- | --- |
|  |  |  | M | | | | | | |
| Age | L | S | 3 | 5 | 25 | 50 | 75 | 95 | 97 |
| 8 | -0.531 | 0.519 | 0.407 | 0.442 | 0.649 | 0.894 | 1.317 | 2.791 | 3.543 |
| 10 | -0.487 | 0.513 | 0.411 | 0.447 | 0.659 | 0.907 | 1.324 | 2.686 | 3.335 |
| 12 | -0.444 | 0.506 | 0.415 | 0.452 | 0.669 | 0.919 | 1.330 | 2.599 | 3.168 |
| 14 | -0.402 | 0.500 | 0.419 | 0.457 | 0.678 | 0.931 | 1.337 | 2.525 | 3.032 |
| 16 | -0.360 | 0.494 | 0.423 | 0.462 | 0.688 | 0.942 | 1.343 | 2.463 | 2.918 |
| 18 | -0.319 | 0.487 | 0.427 | 0.467 | 0.698 | 0.954 | 1.350 | 2.409 | 2.823 |
| 20 | -0.279 | 0.481 | 0.430 | 0.472 | 0.708 | 0.965 | 1.356 | 2.361 | 2.740 |
| 25 | -0.179 | 0.466 | 0.440 | 0.484 | 0.732 | 0.993 | 1.373 | 2.267 | 2.579 |
| 30 | -0.081 | 0.452 | 0.449 | 0.496 | 0.756 | 1.021 | 1.389 | 2.196 | 2.461 |
| 35 | 0.015 | 0.437 | 0.458 | 0.508 | 0.780 | 1.048 | 1.406 | 2.142 | 2.373 |
| 40 | 0.110 | 0.423 | 0.467 | 0.521 | 0.804 | 1.074 | 1.423 | 2.101 | 2.303 |
| 45 | 0.204 | 0.409 | 0.477 | 0.534 | 0.829 | 1.101 | 1.439 | 2.067 | 2.249 |
| 50 | 0.297 | 0.395 | 0.487 | 0.547 | 0.854 | 1.127 | 1.456 | 2.041 | 2.205 |
| 55 | 0.388 | 0.381 | 0.497 | 0.562 | 0.879 | 1.152 | 1.472 | 2.020 | 2.170 |
| 60 | 0.479 | 0.368 | 0.509 | 0.577 | 0.905 | 1.178 | 1.489 | 2.003 | 2.140 |
| 65 | 0.570 | 0.354 | 0.521 | 0.593 | 0.931 | 1.203 | 1.505 | 1.990 | 2.116 |
| 70 | 0.659 | 0.341 | 0.534 | 0.610 | 0.958 | 1.228 | 1.521 | 1.979 | 2.096 |
| 75 | 0.748 | 0.327 | 0.549 | 0.629 | 0.985 | 1.253 | 1.537 | 1.970 | 2.080 |
| 80 | 0.836 | 0.314 | 0.566 | 0.650 | 1.013 | 1.278 | 1.553 | 1.964 | 2.066 |
| 85 | 0.924 | 0.301 | 0.585 | 0.672 | 1.041 | 1.303 | 1.569 | 1.958 | 2.054 |
|  |  |  |  |  |  |  |  |  |  |
