# Supplementary material for: Dual energy X-ray absorptiometry body composition reference values of limbs and trunk from NHANES 1999–2004 with additional visualization methods
Source: PLoS One. 2017 Mar 27;12(3):e0174180. doi: 10.1371/journal.pone.0174180 (PMC5367711; doi:10.1371/journal.pone.0174180)
Supplement: S7 Table — This table provides L, M, and S values to derive average leg LMI Z-scores for 3rd through 97th percentiles for black males ages 8–85. (DOCX) [file pone.0174180.s015.docx]

Table S7: LMS Curve Fit Data providing L, M, and S values for 3^rd^ through 97^th^ percentiles for Black Females Ages 8-85 for Average Leg LMI.

|  | Females | | | | | | | | |
| --- | --- | --- | --- | --- | --- | --- | --- | --- | --- |
|  |  |  | M | | | | | | |
| Age | L | S | 3 | 5 | 25 | 50 | 75 | 95 | 97 |
| 8 | -0.438 | 0.163 | 1.764 | 1.825 | 2.113 | 2.353 | 2.633 | 3.130 | 3.271 |
| 10 | -0.438 | 0.163 | 1.915 | 1.981 | 2.293 | 2.554 | 2.858 | 3.398 | 3.551 |
| 12 | -0.438 | 0.163 | 2.028 | 2.099 | 2.429 | 2.705 | 3.028 | 3.599 | 3.761 |
| 14 | -0.438 | 0.163 | 2.109 | 2.182 | 2.526 | 2.813 | 3.148 | 3.742 | 3.911 |
| 16 | -0.438 | 0.163 | 2.165 | 2.240 | 2.593 | 2.888 | 3.232 | 3.842 | 4.015 |
| 18 | -0.438 | 0.163 | 2.205 | 2.281 | 2.641 | 2.940 | 3.291 | 3.912 | 4.088 |
| 20 | -0.438 | 0.163 | 2.234 | 2.311 | 2.676 | 2.979 | 3.334 | 3.964 | 4.142 |
| 25 | -0.438 | 0.163 | 2.275 | 2.354 | 2.725 | 3.034 | 3.396 | 4.037 | 4.219 |
| 30 | -0.438 | 0.163 | 2.283 | 2.363 | 2.735 | 3.045 | 3.409 | 4.052 | 4.235 |
| 35 | -0.438 | 0.163 | 2.271 | 2.350 | 2.720 | 3.028 | 3.390 | 4.029 | 4.211 |
| 40 | -0.438 | 0.163 | 2.247 | 2.325 | 2.692 | 2.997 | 3.355 | 3.988 | 4.168 |
| 45 | -0.438 | 0.163 | 2.219 | 2.296 | 2.658 | 2.960 | 3.313 | 3.938 | 4.116 |
| 50 | -0.438 | 0.163 | 2.190 | 2.266 | 2.623 | 2.921 | 3.269 | 3.886 | 4.061 |
| 55 | -0.438 | 0.163 | 2.161 | 2.236 | 2.589 | 2.882 | 3.226 | 3.835 | 4.008 |
| 60 | -0.438 | 0.163 | 2.134 | 2.208 | 2.556 | 2.846 | 3.186 | 3.787 | 3.957 |
| 65 | -0.438 | 0.163 | 2.108 | 2.182 | 2.526 | 2.812 | 3.147 | 3.741 | 3.910 |
| 70 | -0.438 | 0.163 | 2.084 | 2.157 | 2.497 | 2.780 | 3.111 | 3.699 | 3.865 |
| 75 | -0.438 | 0.163 | 2.061 | 2.133 | 2.469 | 2.749 | 3.077 | 3.658 | 3.823 |
| 80 | -0.438 | 0.163 | 2.040 | 2.111 | 2.444 | 2.721 | 3.045 | 3.620 | 3.783 |
| 85 | -0.438 | 0.163 | 2.019 | 2.090 | 2.419 | 2.694 | 3.015 | 3.584 | 3.745 |
|  |  |  |  |  |  |  |  |  |  |
