# Supplementary material for: Dual energy X-ray absorptiometry body composition reference values of limbs and trunk from NHANES 1999–2004 with additional visualization methods
Source: PLoS One. 2017 Mar 27;12(3):e0174180. doi: 10.1371/journal.pone.0174180 (PMC5367711; doi:10.1371/journal.pone.0174180)
Supplement: S8 Table — This table provides L, M, and S values to derive average leg LMI Z-scores for 3rd through 97th percentiles for black males ages 8–85. (DOCX) [file pone.0174180.s016.docx]

Table S8: LMS Curve Fit Data providing L, M, and S values for 3^rd^ through 97^th^ percentiles for Black Males Ages 8-85 for Average Leg LMI.

|  | Males | | | | | | | | |
| --- | --- | --- | --- | --- | --- | --- | --- | --- | --- |
|  |  |  | M | | | | | | |
| Age | L | S | 3 | 5 | 25 | 50 | 75 | 95 | 97 |
| 8 | 0.118 | 0.147 | 1.668 | 1.729 | 2.001 | 2.211 | 2.440 | 2.807 | 2.903 |
| 10 | 0.118 | 0.147 | 1.937 | 2.008 | 2.323 | 2.567 | 2.833 | 3.259 | 3.371 |
| 12 | 0.118 | 0.147 | 2.169 | 2.248 | 2.601 | 2.874 | 3.172 | 3.649 | 3.774 |
| 14 | 0.118 | 0.147 | 2.357 | 2.443 | 2.826 | 3.123 | 3.447 | 3.965 | 4.101 |
| 16 | 0.118 | 0.147 | 2.485 | 2.576 | 2.980 | 3.293 | 3.634 | 4.181 | 4.325 |
| 18 | 0.118 | 0.147 | 2.562 | 2.656 | 3.073 | 3.396 | 3.748 | 4.311 | 4.459 |
| 20 | 0.118 | 0.147 | 2.609 | 2.704 | 3.129 | 3.457 | 3.816 | 4.390 | 4.540 |
| 25 | 0.118 | 0.147 | 2.661 | 2.758 | 3.191 | 3.526 | 3.892 | 4.477 | 4.631 |
| 30 | 0.118 | 0.147 | 2.664 | 2.761 | 3.195 | 3.530 | 3.896 | 4.482 | 4.636 |
| 35 | 0.118 | 0.147 | 2.643 | 2.739 | 3.169 | 3.502 | 3.865 | 4.447 | 4.599 |
| 40 | 0.118 | 0.147 | 2.609 | 2.704 | 3.129 | 3.457 | 3.815 | 4.389 | 4.540 |
| 45 | 0.118 | 0.147 | 2.568 | 2.662 | 3.080 | 3.403 | 3.756 | 4.321 | 4.469 |
| 50 | 0.118 | 0.147 | 2.526 | 2.618 | 3.029 | 3.347 | 3.694 | 4.250 | 4.395 |
| 55 | 0.118 | 0.147 | 2.482 | 2.573 | 2.977 | 3.289 | 3.630 | 4.176 | 4.320 |
| 60 | 0.118 | 0.147 | 2.438 | 2.527 | 2.924 | 3.231 | 3.566 | 4.103 | 4.244 |
| 65 | 0.118 | 0.147 | 2.395 | 2.482 | 2.872 | 3.174 | 3.503 | 4.030 | 4.168 |
| 70 | 0.118 | 0.147 | 2.352 | 2.438 | 2.821 | 3.117 | 3.440 | 3.958 | 4.093 |
| 75 | 0.118 | 0.147 | 2.310 | 2.395 | 2.771 | 3.062 | 3.379 | 3.888 | 4.021 |
| 80 | 0.118 | 0.147 | 2.271 | 2.354 | 2.723 | 3.009 | 3.321 | 3.821 | 3.952 |
| 85 | 0.118 | 0.147 | 2.233 | 2.315 | 2.678 | 2.959 | 3.266 | 3.758 | 3.887 |
|  |  |  |  |  |  |  |  |  |  |
