# Supplementary material for: Dual energy X-ray absorptiometry body composition reference values of limbs and trunk from NHANES 1999–2004 with additional visualization methods
Source: PLoS One. 2017 Mar 27;12(3):e0174180. doi: 10.1371/journal.pone.0174180 (PMC5367711; doi:10.1371/journal.pone.0174180)
Supplement: S9 Table — This table provides L, M, and S values to derive trunk FMI Z-scores for 3rd through 97th percentiles for black females ages 8–85. (DOCX) [file pone.0174180.s017.docx]

Table S9: LMS Curve Fit Data providing L, M, and S values for 3^rd^ through 97^th^ percentiles for Black Females Ages 8-85 for Trunk FMI.

|  | Females | | | | | | | | |
| --- | --- | --- | --- | --- | --- | --- | --- | --- | --- |
|  |  |  | M | | | | | | |
| Age | L | S | 3 | 5 | 25 | 50 | 75 | 95 | 97 |
| 8 | -0.516 | 0.586 | 0.682 | 0.746 | 1.139 | 1.633 | 2.540 | 6.200 | 8.345 |
| 10 | -0.419 | 0.568 | 0.833 | 0.916 | 1.413 | 2.015 | 3.059 | 6.594 | 8.308 |
| 12 | -0.333 | 0.552 | 0.970 | 1.070 | 1.666 | 2.365 | 3.517 | 6.968 | 8.447 |
| 14 | -0.255 | 0.537 | 1.097 | 1.215 | 1.907 | 2.696 | 3.941 | 7.334 | 8.668 |
| 16 | -0.184 | 0.523 | 1.216 | 1.351 | 2.135 | 3.005 | 4.328 | 7.674 | 8.905 |
| 18 | -0.117 | 0.511 | 1.324 | 1.477 | 2.348 | 3.291 | 4.678 | 7.975 | 9.126 |
| 20 | -0.056 | 0.499 | 1.423 | 1.592 | 2.547 | 3.554 | 4.992 | 8.238 | 9.324 |
| 25 | 0.085 | 0.473 | 1.632 | 1.840 | 2.979 | 4.114 | 5.634 | 8.738 | 9.698 |
| 30 | 0.209 | 0.449 | 1.792 | 2.035 | 3.322 | 4.542 | 6.092 | 9.032 | 9.892 |
| 35 | 0.321 | 0.428 | 1.913 | 2.186 | 3.588 | 4.857 | 6.400 | 9.164 | 9.941 |
| 40 | 0.424 | 0.409 | 2.009 | 2.308 | 3.799 | 5.092 | 6.607 | 9.199 | 9.904 |
| 45 | 0.519 | 0.391 | 2.088 | 2.411 | 3.969 | 5.268 | 6.742 | 9.171 | 9.815 |
| 50 | 0.609 | 0.374 | 2.156 | 2.499 | 4.106 | 5.397 | 6.821 | 9.097 | 9.688 |
| 55 | 0.693 | 0.358 | 2.216 | 2.575 | 4.212 | 5.484 | 6.854 | 8.984 | 9.528 |
| 60 | 0.773 | 0.343 | 2.269 | 2.641 | 4.293 | 5.537 | 6.848 | 8.841 | 9.342 |
| 65 | 0.848 | 0.328 | 2.318 | 2.700 | 4.352 | 5.560 | 6.811 | 8.674 | 9.137 |
| 70 | 0.921 | 0.315 | 2.364 | 2.752 | 4.391 | 5.559 | 6.748 | 8.489 | 8.917 |
| 75 | 0.990 | 0.302 | 2.409 | 2.800 | 4.416 | 5.541 | 6.668 | 8.295 | 8.691 |
| 80 | 1.057 | 0.289 | 2.456 | 2.848 | 4.432 | 5.512 | 6.580 | 8.102 | 8.470 |
| 85 | 1.121 | 0.277 | 2.508 | 2.898 | 4.446 | 5.482 | 6.494 | 7.920 | 8.262 |
|  |  |  |  |  |  |  |  |  |  |
