# Supplementary material for: Dual energy X-ray absorptiometry body composition reference values of limbs and trunk from NHANES 1999–2004 with additional visualization methods
Source: PLoS One. 2017 Mar 27;12(3):e0174180. doi: 10.1371/journal.pone.0174180 (PMC5367711; doi:10.1371/journal.pone.0174180)
Supplement: S10 Table — This table provides L, M, and S values to derive trunk FMI Z-scores for 3rd through 97th percentiles for black males ages 8–85. (DOCX) [file pone.0174180.s018.docx]

Table S10: LMS Curve Fit Data providing L, M, and S values for 3^rd^ through 97^th^ percentiles for Black Males Ages 8-85 for Trunk FMI.

|  | Males | | | | | | | | |
| --- | --- | --- | --- | --- | --- | --- | --- | --- | --- |
|  |  |  | M | | | | | | |
| Age | L | S | 3 | 5 | 25 | 50 | 75 | 95 | 97 |
| 8 | -1.120 | 0.458 | 0.680 | 0.720 | 0.954 | 1.244 | 1.817 | 6.541 | 25.065 |
| 10 | -1.011 | 0.458 | 0.704 | 0.748 | 1.001 | 1.309 | 1.895 | 5.410 | 9.926 |
| 12 | -0.912 | 0.458 | 0.728 | 0.774 | 1.047 | 1.374 | 1.975 | 4.914 | 7.439 |
| 14 | -0.820 | 0.458 | 0.753 | 0.803 | 1.097 | 1.445 | 2.062 | 4.672 | 6.449 |
| 16 | -0.736 | 0.458 | 0.782 | 0.837 | 1.154 | 1.524 | 2.163 | 4.571 | 5.971 |
| 18 | -0.656 | 0.458 | 0.816 | 0.876 | 1.219 | 1.615 | 2.281 | 4.568 | 5.751 |
| 20 | -0.580 | 0.458 | 0.856 | 0.921 | 1.295 | 1.721 | 2.419 | 4.636 | 5.684 |
| 25 | -0.407 | 0.458 | 0.966 | 1.048 | 1.512 | 2.022 | 2.812 | 4.973 | 5.843 |
| 30 | -0.250 | 0.458 | 1.065 | 1.165 | 1.726 | 2.323 | 3.204 | 5.356 | 6.133 |
| 35 | -0.106 | 0.458 | 1.139 | 1.258 | 1.917 | 2.597 | 3.555 | 5.697 | 6.412 |
| 40 | 0.028 | 0.458 | 1.186 | 1.324 | 2.080 | 2.836 | 3.857 | 5.979 | 6.646 |
| 45 | 0.154 | 0.458 | 1.206 | 1.365 | 2.216 | 3.041 | 4.112 | 6.204 | 6.830 |
| 50 | 0.273 | 0.458 | 1.205 | 1.384 | 2.331 | 3.218 | 4.328 | 6.385 | 6.977 |
| 55 | 0.387 | 0.458 | 1.183 | 1.384 | 2.428 | 3.373 | 4.515 | 6.534 | 7.096 |
| 60 | 0.495 | 0.458 | 1.142 | 1.367 | 2.511 | 3.510 | 4.679 | 6.661 | 7.195 |
| 65 | 0.599 | 0.458 | 1.082 | 1.334 | 2.584 | 3.635 | 4.825 | 6.770 | 7.282 |
| 70 | 0.699 | 0.458 | 1.003 | 1.286 | 2.648 | 3.749 | 4.959 | 6.869 | 7.360 |
| 75 | 0.795 | 0.458 | 0.901 | 1.221 | 2.705 | 3.855 | 5.081 | 6.958 | 7.432 |
| 80 | 0.888 | 0.458 | 0.773 | 1.137 | 2.757 | 3.955 | 5.196 | 7.042 | 7.500 |
| 85 | 0.979 | 0.458 | 0.609 | 1.033 | 2.805 | 4.051 | 5.306 | 7.124 | 7.568 |
|  |  |  |  |  |  |  |  |  |  |
