# Supplementary material for: Dual energy X-ray absorptiometry body composition reference values of limbs and trunk from NHANES 1999–2004 with additional visualization methods
Source: PLoS One. 2017 Mar 27;12(3):e0174180. doi: 10.1371/journal.pone.0174180 (PMC5367711; doi:10.1371/journal.pone.0174180)
Supplement: S11 Table — This table provides L, M, and S values to derive trunk LMI Z-scores for 3rd through 97th percentiles for black females ages 8–85. (DOCX) [file pone.0174180.s019.docx]

Table S11: LMS Curve Fit Data providing L, M, and S values for 3^rd^ through 97^th^ percentiles for Black Females Ages 8-85 for Trunk LMI.

|  | Females | | | | | | | | |
| --- | --- | --- | --- | --- | --- | --- | --- | --- | --- |
|  |  |  | M | | | | | | |
| Age | L | S | 3 | 5 | 25 | 50 | 75 | 95 | 97 |
| 8 | -0.472 | 0.131 | 4.429 | 4.554 | 5.127 | 5.589 | 6.115 | 7.012 | 7.259 |
| 10 | -0.472 | 0.131 | 4.933 | 5.072 | 5.710 | 6.225 | 6.811 | 7.809 | 8.085 |
| 12 | -0.472 | 0.131 | 5.325 | 5.475 | 6.163 | 6.719 | 7.352 | 8.429 | 8.726 |
| 14 | -0.472 | 0.131 | 5.618 | 5.776 | 6.503 | 7.089 | 7.757 | 8.894 | 9.207 |
| 16 | -0.472 | 0.131 | 5.832 | 5.996 | 6.751 | 7.359 | 8.052 | 9.232 | 9.558 |
| 18 | -0.472 | 0.131 | 5.990 | 6.159 | 6.934 | 7.559 | 8.271 | 9.483 | 9.817 |
| 20 | -0.472 | 0.131 | 6.110 | 6.283 | 7.073 | 7.711 | 8.437 | 9.674 | 10.014 |
| 25 | -0.472 | 0.131 | 6.306 | 6.484 | 7.300 | 7.958 | 8.707 | 9.984 | 10.335 |
| 30 | -0.472 | 0.131 | 6.410 | 6.591 | 7.420 | 8.089 | 8.850 | 10.148 | 10.505 |
| 35 | -0.472 | 0.131 | 6.463 | 6.645 | 7.481 | 8.156 | 8.924 | 10.231 | 10.592 |
| 40 | -0.472 | 0.131 | 6.490 | 6.673 | 7.513 | 8.190 | 8.961 | 10.275 | 10.636 |
| 45 | -0.472 | 0.131 | 6.503 | 6.686 | 7.527 | 8.206 | 8.979 | 10.295 | 10.657 |
| 50 | -0.472 | 0.131 | 6.507 | 6.691 | 7.532 | 8.211 | 8.985 | 10.301 | 10.664 |
| 55 | -0.472 | 0.131 | 6.506 | 6.690 | 7.531 | 8.210 | 8.983 | 10.300 | 10.663 |
| 60 | -0.472 | 0.131 | 6.503 | 6.686 | 7.527 | 8.206 | 8.979 | 10.295 | 10.657 |
| 65 | -0.472 | 0.131 | 6.497 | 6.681 | 7.521 | 8.199 | 8.971 | 10.286 | 10.649 |
| 70 | -0.472 | 0.131 | 6.491 | 6.674 | 7.514 | 8.191 | 8.963 | 10.276 | 10.638 |
| 75 | -0.472 | 0.131 | 6.484 | 6.667 | 7.506 | 8.183 | 8.953 | 10.265 | 10.627 |
| 80 | -0.472 | 0.131 | 6.477 | 6.660 | 7.498 | 8.174 | 8.944 | 10.254 | 10.616 |
| 85 | -0.472 | 0.131 | 6.471 | 6.653 | 7.490 | 8.165 | 8.934 | 10.244 | 10.605 |
|  |  |  |  |  |  |  |  |  |  |
