# Supplementary material for: Dual energy X-ray absorptiometry body composition reference values of limbs and trunk from NHANES 1999–2004 with additional visualization methods
Source: PLoS One. 2017 Mar 27;12(3):e0174180. doi: 10.1371/journal.pone.0174180 (PMC5367711; doi:10.1371/journal.pone.0174180)
Supplement: S12 Table — This table provides L, M, and S values to derive trunk LMI Z-scores for 3rd through 97th percentiles for black males ages 8–85. (DOCX) [file pone.0174180.s020.docx]

Table S12: LMS Curve Fit Data providing L, M, and S values for 3^rd^ through 97^th^ percentiles for Black Males Ages 8-85 for Trunk LMI.

|  | Males | | | | | | | | |
| --- | --- | --- | --- | --- | --- | --- | --- | --- | --- |
|  |  |  | M | | | | | | |
| Age | L | S | 3 | 5 | 25 | 50 | 75 | 95 | 97 |
| 8 | 0.110 | 0.132 | 4.317 | 4.458 | 5.080 | 5.557 | 6.072 | 6.890 | 7.103 |
| 10 | 0.110 | 0.132 | 4.729 | 4.883 | 5.564 | 6.086 | 6.651 | 7.547 | 7.780 |
| 12 | 0.110 | 0.132 | 5.218 | 5.388 | 6.141 | 6.717 | 7.340 | 8.328 | 8.586 |
| 14 | 0.110 | 0.132 | 5.779 | 5.967 | 6.800 | 7.437 | 8.128 | 9.222 | 9.507 |
| 16 | 0.110 | 0.132 | 6.237 | 6.440 | 7.339 | 8.027 | 8.772 | 9.953 | 10.261 |
| 18 | 0.110 | 0.132 | 6.538 | 6.751 | 7.694 | 8.415 | 9.196 | 10.435 | 10.757 |
| 20 | 0.110 | 0.132 | 6.737 | 6.956 | 7.927 | 8.671 | 9.476 | 10.752 | 11.084 |
| 25 | 0.110 | 0.132 | 7.029 | 7.258 | 8.271 | 9.046 | 9.886 | 11.217 | 11.564 |
| 30 | 0.110 | 0.132 | 7.157 | 7.390 | 8.422 | 9.212 | 10.067 | 11.422 | 11.776 |
| 35 | 0.110 | 0.132 | 7.213 | 7.448 | 8.488 | 9.284 | 10.145 | 11.512 | 11.868 |
| 40 | 0.110 | 0.132 | 7.239 | 7.475 | 8.518 | 9.317 | 10.182 | 11.553 | 11.910 |
| 45 | 0.110 | 0.132 | 7.244 | 7.480 | 8.524 | 9.323 | 10.189 | 11.561 | 11.918 |
| 50 | 0.110 | 0.132 | 7.234 | 7.469 | 8.512 | 9.310 | 10.174 | 11.544 | 11.901 |
| 55 | 0.110 | 0.132 | 7.207 | 7.441 | 8.480 | 9.275 | 10.136 | 11.501 | 11.857 |
| 60 | 0.110 | 0.132 | 7.164 | 7.397 | 8.430 | 9.221 | 10.077 | 11.434 | 11.787 |
| 65 | 0.110 | 0.132 | 7.108 | 7.340 | 8.364 | 9.149 | 9.998 | 11.344 | 11.695 |
| 70 | 0.110 | 0.132 | 7.041 | 7.270 | 8.286 | 9.062 | 9.903 | 11.237 | 11.585 |
| 75 | 0.110 | 0.132 | 6.969 | 7.196 | 8.201 | 8.970 | 9.802 | 11.122 | 11.466 |
| 80 | 0.110 | 0.132 | 6.897 | 7.122 | 8.117 | 8.878 | 9.701 | 11.008 | 11.348 |
| 85 | 0.110 | 0.132 | 6.829 | 7.051 | 8.036 | 8.789 | 9.605 | 10.899 | 11.236 |
|  |  |  |  |  |  |  |  |  |  |
