# Supplementary material for: Dual energy X-ray absorptiometry body composition reference values of limbs and trunk from NHANES 1999–2004 with additional visualization methods
Source: PLoS One. 2017 Mar 27;12(3):e0174180. doi: 10.1371/journal.pone.0174180 (PMC5367711; doi:10.1371/journal.pone.0174180)
Supplement: S13 Table — This table provides L, M, and S values to derive total body FMI Z-scores for 3rd through 97th percentiles for black females ages 8–85. (DOCX) [file pone.0174180.s021.docx]

Table S13: LMS Curve Fit Data providing L, M, and S values for 3^rd^ through 97^th^ percentiles for Black Females Ages 8-85 for Total Body FMI.

|  | Females | | | | | | | | |
| --- | --- | --- | --- | --- | --- | --- | --- | --- | --- |
|  |  |  | M | | | | | | |
| Age | L | S | 3 | 5 | 25 | 50 | 75 | 95 | 97 |
| 8 | -0.602 | 0.493 | 2.445 | 2.639 | 3.773 | 5.108 | 7.399 | 15.529 | 19.823 |
| 10 | -0.458 | 0.472 | 2.862 | 3.102 | 4.478 | 6.025 | 8.495 | 15.710 | 18.805 |
| 12 | -0.340 | 0.454 | 3.205 | 3.487 | 5.071 | 6.786 | 9.377 | 16.072 | 18.623 |
| 14 | -0.241 | 0.440 | 3.501 | 3.821 | 5.590 | 7.442 | 10.121 | 16.472 | 18.712 |
| 16 | -0.154 | 0.427 | 3.759 | 4.114 | 6.047 | 8.013 | 10.755 | 16.850 | 18.884 |
| 18 | -0.078 | 0.416 | 3.984 | 4.371 | 6.449 | 8.509 | 11.295 | 17.183 | 19.068 |
| 20 | -0.011 | 0.406 | 4.182 | 4.599 | 6.806 | 8.942 | 11.758 | 17.469 | 19.238 |
| 25 | 0.133 | 0.384 | 4.584 | 5.065 | 7.534 | 9.807 | 12.651 | 17.996 | 19.557 |
| 30 | 0.251 | 0.367 | 4.889 | 5.422 | 8.083 | 10.435 | 13.266 | 18.309 | 19.726 |
| 35 | 0.350 | 0.352 | 5.126 | 5.701 | 8.506 | 10.899 | 13.692 | 18.475 | 19.781 |
| 40 | 0.437 | 0.340 | 5.320 | 5.930 | 8.841 | 11.253 | 13.996 | 18.552 | 19.769 |
| 45 | 0.513 | 0.328 | 5.485 | 6.124 | 9.115 | 11.530 | 14.220 | 18.577 | 19.721 |
| 50 | 0.581 | 0.318 | 5.630 | 6.294 | 9.346 | 11.753 | 14.388 | 18.569 | 19.652 |
| 55 | 0.642 | 0.309 | 5.761 | 6.447 | 9.544 | 11.938 | 14.519 | 18.543 | 19.574 |
| 60 | 0.698 | 0.301 | 5.883 | 6.587 | 9.718 | 12.096 | 14.623 | 18.508 | 19.493 |
| 65 | 0.750 | 0.293 | 5.999 | 6.718 | 9.875 | 12.232 | 14.710 | 18.469 | 19.414 |
| 70 | 0.798 | 0.286 | 6.110 | 6.842 | 10.018 | 12.354 | 14.784 | 18.429 | 19.339 |
| 75 | 0.842 | 0.280 | 6.218 | 6.962 | 10.150 | 12.464 | 14.849 | 18.392 | 19.270 |
| 80 | 0.884 | 0.274 | 6.323 | 7.078 | 10.274 | 12.566 | 14.908 | 18.357 | 19.207 |
| 85 | 0.923 | 0.268 | 6.427 | 7.190 | 10.391 | 12.661 | 14.962 | 18.326 | 19.151 |
|  |  |  |  |  |  |  |  |  |  |
