# Supplementary material for: Dual energy X-ray absorptiometry body composition reference values of limbs and trunk from NHANES 1999–2004 with additional visualization methods
Source: PLoS One. 2017 Mar 27;12(3):e0174180. doi: 10.1371/journal.pone.0174180 (PMC5367711; doi:10.1371/journal.pone.0174180)
Supplement: S14 Table — This table provides L, M, and S values to derive total body FMI Z-scores for 3rd through 97th percentiles for black males ages 8–85. (DOCX) [file pone.0174180.s022.docx]

Table S14: LMS Curve Fit Data providing L, M, and S values for 3^rd^ through 97^th^ percentiles for Black Males Ages 8-85 for Total Body FMI.

|  | Males | | | | | | | | |
| --- | --- | --- | --- | --- | --- | --- | --- | --- | --- |
|  |  |  | M | | | | | | |
| Age | L | S | 3 | 5 | 25 | 50 | 75 | 95 | 97 |
| 8 | -1.125 | 0.410 | 2.302 | 2.428 | 3.153 | 4.009 | 5.581 | 14.167 | 24.111 |
| 10 | -1.016 | 0.410 | 2.297 | 2.429 | 3.182 | 4.058 | 5.612 | 12.643 | 18.263 |
| 12 | -0.918 | 0.410 | 2.292 | 2.430 | 3.210 | 4.106 | 5.646 | 11.733 | 15.656 |
| 14 | -0.827 | 0.410 | 2.291 | 2.434 | 3.243 | 4.158 | 5.689 | 11.140 | 14.175 |
| 16 | -0.743 | 0.410 | 2.299 | 2.449 | 3.290 | 4.229 | 5.761 | 10.769 | 13.272 |
| 18 | -0.663 | 0.410 | 2.325 | 2.482 | 3.362 | 4.333 | 5.878 | 10.585 | 12.747 |
| 20 | -0.588 | 0.410 | 2.369 | 2.536 | 3.463 | 4.473 | 6.046 | 10.554 | 12.489 |
| 25 | -0.416 | 0.410 | 2.525 | 2.719 | 3.793 | 4.926 | 6.605 | 10.863 | 12.474 |
| 30 | -0.260 | 0.410 | 2.674 | 2.901 | 4.135 | 5.398 | 7.189 | 11.325 | 12.757 |
| 35 | -0.117 | 0.410 | 2.783 | 3.043 | 4.436 | 5.821 | 7.708 | 11.746 | 13.054 |
| 40 | 0.017 | 0.410 | 2.845 | 3.137 | 4.685 | 6.179 | 8.138 | 12.077 | 13.287 |
| 45 | 0.142 | 0.410 | 2.862 | 3.188 | 4.884 | 6.473 | 8.486 | 12.320 | 13.449 |
| 50 | 0.260 | 0.410 | 2.843 | 3.203 | 5.046 | 6.720 | 8.773 | 12.503 | 13.563 |
| 55 | 0.373 | 0.410 | 2.795 | 3.190 | 5.180 | 6.933 | 9.017 | 12.648 | 13.648 |
| 60 | 0.481 | 0.410 | 2.720 | 3.154 | 5.296 | 7.122 | 9.230 | 12.768 | 13.718 |
| 65 | 0.584 | 0.410 | 2.619 | 3.097 | 5.397 | 7.293 | 9.420 | 12.873 | 13.779 |
| 70 | 0.684 | 0.410 | 2.493 | 3.018 | 5.484 | 7.448 | 9.591 | 12.964 | 13.831 |
| 75 | 0.780 | 0.410 | 2.337 | 2.918 | 5.561 | 7.589 | 9.745 | 13.044 | 13.876 |
| 80 | 0.872 | 0.410 | 2.149 | 2.794 | 5.629 | 7.720 | 9.887 | 13.116 | 13.918 |
| 85 | 0.962 | 0.410 | 1.923 | 2.647 | 5.691 | 7.845 | 10.022 | 13.189 | 13.963 |
|  |  |  |  |  |  |  |  |  |  |
