# Supplementary material for: Dual energy X-ray absorptiometry body composition reference values of limbs and trunk from NHANES 1999–2004 with additional visualization methods
Source: PLoS One. 2017 Mar 27;12(3):e0174180. doi: 10.1371/journal.pone.0174180 (PMC5367711; doi:10.1371/journal.pone.0174180)
Supplement: S16 Table — This table provides L, M, and S values to derive total body LMI Z-scores for 3rd through 97th percentiles for black males ages 8–85. (DOCX) [file pone.0174180.s024.docx]

Table S16: LMS Curve Fit Data providing L, M, and S values for 3^rd^ through 97^th^ percentiles for Black Males Ages 8-85 for Total Body LMI.

|  | Males | | | | | | | | |
| --- | --- | --- | --- | --- | --- | --- | --- | --- | --- |
|  |  |  | M | | | | | | |
| Age | L | S | 3 | 5 | 25 | 50 | 75 | 95 | 97 |
| 8 | -0.898 | 0.126 | 10.556 | 10.821 | 12.062 | 13.095 | 14.309 | 16.485 | 17.112 |
| 10 | -0.681 | 0.126 | 11.434 | 11.734 | 13.120 | 14.254 | 15.562 | 17.838 | 18.476 |
| 12 | -0.503 | 0.126 | 12.453 | 12.792 | 14.342 | 15.590 | 17.009 | 19.421 | 20.085 |
| 14 | -0.353 | 0.126 | 13.561 | 13.942 | 15.668 | 17.041 | 18.581 | 21.150 | 21.846 |
| 16 | -0.223 | 0.126 | 14.431 | 14.848 | 16.722 | 18.196 | 19.831 | 22.514 | 23.233 |
| 18 | -0.108 | 0.126 | 14.978 | 15.421 | 17.403 | 18.943 | 20.637 | 23.379 | 24.105 |
| 20 | -0.005 | 0.126 | 15.322 | 15.786 | 17.846 | 19.434 | 21.163 | 23.930 | 24.656 |
| 25 | 0.212 | 0.126 | 15.785 | 16.288 | 18.488 | 20.149 | 21.925 | 24.697 | 25.411 |
| 30 | 0.390 | 0.126 | 15.916 | 16.445 | 18.732 | 20.429 | 22.215 | 24.952 | 25.646 |
| 35 | 0.540 | 0.126 | 15.881 | 16.430 | 18.774 | 20.486 | 22.266 | 24.950 | 25.623 |
| 40 | 0.670 | 0.126 | 15.771 | 16.334 | 18.719 | 20.436 | 22.202 | 24.829 | 25.482 |
| 45 | 0.785 | 0.126 | 15.620 | 16.195 | 18.608 | 20.324 | 22.072 | 24.643 | 25.277 |
| 50 | 0.888 | 0.126 | 15.450 | 16.035 | 18.470 | 20.181 | 21.909 | 24.426 | 25.042 |
| 55 | 0.981 | 0.126 | 15.257 | 15.851 | 18.300 | 20.003 | 21.710 | 24.172 | 24.771 |
| 60 | 1.065 | 0.126 | 15.043 | 15.644 | 18.100 | 19.791 | 21.473 | 23.882 | 24.465 |
| 65 | 1.143 | 0.126 | 14.811 | 15.416 | 17.872 | 19.549 | 21.205 | 23.560 | 24.127 |
| 70 | 1.216 | 0.126 | 14.562 | 15.171 | 17.623 | 19.282 | 20.911 | 23.211 | 23.763 |
| 75 | 1.283 | 0.126 | 14.310 | 14.921 | 17.366 | 19.006 | 20.607 | 22.854 | 23.391 |
| 80 | 1.346 | 0.126 | 14.066 | 14.679 | 17.114 | 18.735 | 20.310 | 22.507 | 23.029 |
| 85 | 1.405 | 0.126 | 13.833 | 14.447 | 16.874 | 18.477 | 20.026 | 22.176 | 22.686 |
|  |  |  |  |  |  |  |  |  |  |
