# Supplementary material for: Dual energy X-ray absorptiometry body composition reference values of limbs and trunk from NHANES 1999–2004 with additional visualization methods
Source: PLoS One. 2017 Mar 27;12(3):e0174180. doi: 10.1371/journal.pone.0174180 (PMC5367711; doi:10.1371/journal.pone.0174180)
Supplement: S17 Table — This table provides L, M, and S values to derive average arm FMI Z-scores for 3rd through 97th percentiles for Hispanic females ages 8–85. (DOCX) [file pone.0174180.s025.docx]

Table S17: LMS Curve Fit Data providing L, M, and S values for 3^rd^ through 97^th^ percentiles for Hispanic Females Ages 8-85 for Average Arm FMI.

|  | Females | | | | | | | | |
| --- | --- | --- | --- | --- | --- | --- | --- | --- | --- |
|  |  |  | M | | | | | | |
| Age | M | S | 3 | 5 | 25 | 50 | 75 | 95 | 97 |
| 8 | 0.314 | 0.454 | 0.151 | 0.164 | 0.235 | 0.314 | 0.435 | 0.769 | 0.904 |
| 10 | 0.366 | 0.440 | 0.176 | 0.192 | 0.276 | 0.366 | 0.500 | 0.837 | 0.963 |
| 12 | 0.410 | 0.428 | 0.197 | 0.215 | 0.310 | 0.410 | 0.553 | 0.892 | 1.012 |
| 14 | 0.447 | 0.418 | 0.216 | 0.235 | 0.340 | 0.447 | 0.598 | 0.937 | 1.053 |
| 16 | 0.480 | 0.409 | 0.232 | 0.253 | 0.367 | 0.480 | 0.636 | 0.977 | 1.089 |
| 18 | 0.510 | 0.401 | 0.247 | 0.270 | 0.391 | 0.510 | 0.671 | 1.011 | 1.120 |
| 20 | 0.536 | 0.394 | 0.260 | 0.284 | 0.412 | 0.536 | 0.701 | 1.040 | 1.147 |
| 25 | 0.590 | 0.380 | 0.288 | 0.315 | 0.457 | 0.590 | 0.762 | 1.099 | 1.200 |
| 30 | 0.633 | 0.368 | 0.310 | 0.340 | 0.493 | 0.633 | 0.809 | 1.141 | 1.238 |
| 35 | 0.667 | 0.358 | 0.328 | 0.360 | 0.522 | 0.667 | 0.845 | 1.172 | 1.266 |
| 40 | 0.694 | 0.349 | 0.344 | 0.378 | 0.546 | 0.694 | 0.874 | 1.196 | 1.287 |
| 45 | 0.717 | 0.341 | 0.357 | 0.392 | 0.566 | 0.717 | 0.897 | 1.214 | 1.302 |
| 50 | 0.736 | 0.334 | 0.369 | 0.405 | 0.584 | 0.736 | 0.916 | 1.228 | 1.313 |
| 55 | 0.753 | 0.328 | 0.379 | 0.416 | 0.598 | 0.753 | 0.932 | 1.238 | 1.322 |
| 60 | 0.767 | 0.322 | 0.388 | 0.426 | 0.612 | 0.767 | 0.945 | 1.246 | 1.328 |
| 65 | 0.779 | 0.317 | 0.396 | 0.435 | 0.623 | 0.779 | 0.957 | 1.253 | 1.333 |
| 70 | 0.790 | 0.312 | 0.403 | 0.444 | 0.634 | 0.790 | 0.967 | 1.259 | 1.337 |
| 75 | 0.800 | 0.308 | 0.411 | 0.452 | 0.644 | 0.800 | 0.976 | 1.264 | 1.341 |
| 80 | 0.810 | 0.303 | 0.417 | 0.459 | 0.653 | 0.810 | 0.985 | 1.269 | 1.344 |
| 85 | 0.819 | 0.299 | 0.424 | 0.466 | 0.662 | 0.819 | 0.993 | 1.273 | 1.347 |
|  |  |  |  |  |  |  |  |  |  |
