# Supplementary material for: Dual energy X-ray absorptiometry body composition reference values of limbs and trunk from NHANES 1999–2004 with additional visualization methods
Source: PLoS One. 2017 Mar 27;12(3):e0174180. doi: 10.1371/journal.pone.0174180 (PMC5367711; doi:10.1371/journal.pone.0174180)
Supplement: S18 Table — This table provides L, M, and S values to derive average arm FMI Z-scores for 3rd through 97th percentiles for Hispanic males ages 8–85. (DOCX) [file pone.0174180.s026.docx]

Table S18: LMS Curve Fit Data providing L, M, and S values for 3^rd^ through 97^th^ percentiles for Hispanic Males Ages 8-85 for Average Arm FMI.

|  | Males | | | | | | | | |
| --- | --- | --- | --- | --- | --- | --- | --- | --- | --- |
|  |  |  | M | | | | | | |
| Age | L | S | 3 | 5 | 25 | 50 | 75 | 95 | 97 |
| 8 | -0.174 | 0.579 | 0.119 | 0.134 | 0.221 | 0.323 | 0.484 | 0.915 | 1.081 |
| 10 | -0.174 | 0.544 | 0.126 | 0.141 | 0.226 | 0.323 | 0.472 | 0.855 | 0.998 |
| 12 | -0.174 | 0.515 | 0.132 | 0.146 | 0.230 | 0.322 | 0.461 | 0.806 | 0.931 |
| 14 | -0.174 | 0.489 | 0.137 | 0.152 | 0.234 | 0.323 | 0.453 | 0.767 | 0.878 |
| 16 | -0.174 | 0.465 | 0.144 | 0.159 | 0.240 | 0.326 | 0.450 | 0.741 | 0.843 |
| 18 | -0.174 | 0.444 | 0.153 | 0.167 | 0.249 | 0.333 | 0.453 | 0.727 | 0.821 |
| 20 | -0.174 | 0.425 | 0.162 | 0.177 | 0.259 | 0.342 | 0.459 | 0.722 | 0.810 |
| 25 | -0.174 | 0.386 | 0.186 | 0.202 | 0.286 | 0.369 | 0.482 | 0.723 | 0.802 |
| 30 | -0.174 | 0.357 | 0.208 | 0.224 | 0.310 | 0.393 | 0.502 | 0.730 | 0.802 |
| 35 | -0.174 | 0.336 | 0.226 | 0.242 | 0.329 | 0.411 | 0.518 | 0.735 | 0.803 |
| 40 | -0.174 | 0.321 | 0.240 | 0.257 | 0.344 | 0.425 | 0.530 | 0.739 | 0.804 |
| 45 | -0.174 | 0.309 | 0.251 | 0.268 | 0.355 | 0.436 | 0.539 | 0.742 | 0.804 |
| 50 | -0.174 | 0.299 | 0.260 | 0.277 | 0.365 | 0.445 | 0.546 | 0.744 | 0.804 |
| 55 | -0.174 | 0.291 | 0.268 | 0.285 | 0.372 | 0.452 | 0.552 | 0.745 | 0.804 |
| 60 | -0.174 | 0.285 | 0.274 | 0.291 | 0.379 | 0.457 | 0.556 | 0.746 | 0.803 |
| 65 | -0.174 | 0.280 | 0.279 | 0.297 | 0.384 | 0.462 | 0.560 | 0.746 | 0.802 |
| 70 | -0.174 | 0.275 | 0.284 | 0.302 | 0.388 | 0.466 | 0.562 | 0.746 | 0.801 |
| 75 | -0.174 | 0.270 | 0.288 | 0.306 | 0.392 | 0.469 | 0.565 | 0.746 | 0.799 |
| 80 | -0.174 | 0.266 | 0.292 | 0.310 | 0.396 | 0.473 | 0.567 | 0.745 | 0.798 |
| 85 | -0.174 | 0.262 | 0.296 | 0.314 | 0.399 | 0.475 | 0.569 | 0.744 | 0.796 |
|  |  |  |  |  |  |  |  |  |  |
