# Supplementary material for: Dual energy X-ray absorptiometry body composition reference values of limbs and trunk from NHANES 1999–2004 with additional visualization methods
Source: PLoS One. 2017 Mar 27;12(3):e0174180. doi: 10.1371/journal.pone.0174180 (PMC5367711; doi:10.1371/journal.pone.0174180)
Supplement: S19 Table — This table provides L, M, and S values to derive average arm LMI Z-scores for 3rd through 97th percentiles for Hispanic females ages 8–85. (DOCX) [file pone.0174180.s027.docx]

Table S19: LMS Curve Fit Data providing L, M, and S values for 3^rd^ through 97^th^ percentiles for Hispanic Females Ages 8-85 for Average Arm LMI.

|  | Females | | | | | | | | |
| --- | --- | --- | --- | --- | --- | --- | --- | --- | --- |
|  |  |  | M | | | | | | |
| Age | L | S | 3 | 5 | 25 | 50 | 75 | 95 | 97 |
| 8 | -1.204 | 0.162 | 0.487 | 0.501 | 0.570 | 0.632 | 0.710 | 0.871 | 0.924 |
| 10 | -1.073 | 0.162 | 0.525 | 0.541 | 0.617 | 0.684 | 0.768 | 0.936 | 0.989 |
| 12 | -0.962 | 0.162 | 0.556 | 0.573 | 0.655 | 0.726 | 0.815 | 0.988 | 1.042 |
| 14 | -0.865 | 0.162 | 0.580 | 0.598 | 0.684 | 0.760 | 0.852 | 1.028 | 1.082 |
| 16 | -0.779 | 0.162 | 0.598 | 0.617 | 0.707 | 0.786 | 0.881 | 1.059 | 1.113 |
| 18 | -0.701 | 0.162 | 0.612 | 0.632 | 0.726 | 0.807 | 0.904 | 1.084 | 1.137 |
| 20 | -0.629 | 0.162 | 0.624 | 0.644 | 0.742 | 0.824 | 0.923 | 1.104 | 1.156 |
| 25 | -0.473 | 0.162 | 0.645 | 0.667 | 0.771 | 0.858 | 0.959 | 1.140 | 1.192 |
| 30 | -0.339 | 0.162 | 0.658 | 0.682 | 0.790 | 0.880 | 0.983 | 1.163 | 1.214 |
| 35 | -0.223 | 0.162 | 0.666 | 0.690 | 0.802 | 0.894 | 0.998 | 1.177 | 1.226 |
| 40 | -0.119 | 0.162 | 0.668 | 0.693 | 0.809 | 0.901 | 1.006 | 1.182 | 1.230 |
| 45 | -0.025 | 0.162 | 0.667 | 0.693 | 0.810 | 0.903 | 1.008 | 1.181 | 1.227 |
| 50 | 0.061 | 0.162 | 0.662 | 0.689 | 0.807 | 0.901 | 1.004 | 1.173 | 1.218 |
| 55 | 0.140 | 0.162 | 0.655 | 0.682 | 0.801 | 0.894 | 0.997 | 1.162 | 1.206 |
| 60 | 0.214 | 0.162 | 0.646 | 0.673 | 0.793 | 0.886 | 0.987 | 1.148 | 1.190 |
| 65 | 0.283 | 0.162 | 0.636 | 0.663 | 0.783 | 0.875 | 0.974 | 1.132 | 1.172 |
| 70 | 0.348 | 0.162 | 0.626 | 0.653 | 0.773 | 0.864 | 0.961 | 1.114 | 1.154 |
| 75 | 0.409 | 0.162 | 0.615 | 0.642 | 0.762 | 0.852 | 0.948 | 1.097 | 1.135 |
| 80 | 0.467 | 0.162 | 0.605 | 0.632 | 0.751 | 0.840 | 0.935 | 1.080 | 1.117 |
| 85 | 0.522 | 0.162 | 0.595 | 0.622 | 0.741 | 0.829 | 0.922 | 1.064 | 1.100 |
|  |  |  |  |  |  |  |  |  |  |
