# Supplementary material for: Dual energy X-ray absorptiometry body composition reference values of limbs and trunk from NHANES 1999–2004 with additional visualization methods
Source: PLoS One. 2017 Mar 27;12(3):e0174180. doi: 10.1371/journal.pone.0174180 (PMC5367711; doi:10.1371/journal.pone.0174180)
Supplement: S20 Table — This table provides L, M, and S values to derive average arm LMI Z-scores for 3rd through 97th percentiles for Hispanic males ages 8–85. (DOCX) [file pone.0174180.s028.docx]

Table S20: LMS Curve Fit Data providing L, M, and S values for 3^rd^ through 97^th^ percentiles for Hispanic Males Ages 8-85 for Average Arm LMI.

|  | Males | | | | | | | | |
| --- | --- | --- | --- | --- | --- | --- | --- | --- | --- |
|  |  |  | M | | | | | | |
| Age | L | S | 3 | 5 | 25 | 50 | 75 | 95 | 97 |
| 8 | 0.175 | 0.162 | 0.498 | 0.519 | 0.610 | 0.681 | 0.759 | 0.883 | 0.916 |
| 10 | 0.175 | 0.158 | 0.553 | 0.575 | 0.674 | 0.750 | 0.834 | 0.967 | 1.002 |
| 12 | 0.175 | 0.155 | 0.637 | 0.662 | 0.773 | 0.858 | 0.952 | 1.101 | 1.140 |
| 14 | 0.175 | 0.152 | 0.740 | 0.768 | 0.894 | 0.991 | 1.097 | 1.266 | 1.310 |
| 16 | 0.175 | 0.150 | 0.826 | 0.857 | 0.996 | 1.102 | 1.218 | 1.403 | 1.451 |
| 18 | 0.175 | 0.147 | 0.883 | 0.916 | 1.062 | 1.174 | 1.296 | 1.489 | 1.539 |
| 20 | 0.175 | 0.146 | 0.921 | 0.955 | 1.105 | 1.220 | 1.344 | 1.542 | 1.594 |
| 25 | 0.175 | 0.142 | 0.978 | 1.013 | 1.167 | 1.285 | 1.413 | 1.615 | 1.667 |
| 30 | 0.175 | 0.139 | 1.007 | 1.042 | 1.197 | 1.315 | 1.443 | 1.644 | 1.697 |
| 35 | 0.175 | 0.136 | 1.023 | 1.058 | 1.212 | 1.329 | 1.455 | 1.654 | 1.706 |
| 40 | 0.175 | 0.133 | 1.030 | 1.064 | 1.216 | 1.331 | 1.455 | 1.651 | 1.702 |
| 45 | 0.175 | 0.131 | 1.029 | 1.063 | 1.211 | 1.325 | 1.446 | 1.638 | 1.687 |
| 50 | 0.175 | 0.130 | 1.021 | 1.054 | 1.200 | 1.310 | 1.429 | 1.615 | 1.663 |
| 55 | 0.175 | 0.128 | 1.008 | 1.040 | 1.181 | 1.288 | 1.403 | 1.584 | 1.631 |
| 60 | 0.175 | 0.126 | 0.989 | 1.020 | 1.157 | 1.261 | 1.372 | 1.547 | 1.592 |
| 65 | 0.175 | 0.125 | 0.966 | 0.997 | 1.129 | 1.229 | 1.336 | 1.503 | 1.547 |
| 70 | 0.175 | 0.124 | 0.938 | 0.967 | 1.094 | 1.190 | 1.293 | 1.453 | 1.495 |
| 75 | 0.175 | 0.122 | 0.907 | 0.935 | 1.056 | 1.148 | 1.246 | 1.399 | 1.438 |
| 80 | 0.175 | 0.121 | 0.875 | 0.901 | 1.017 | 1.104 | 1.198 | 1.344 | 1.381 |
| 85 | 0.175 | 0.120 | 0.844 | 0.869 | 0.979 | 1.063 | 1.152 | 1.291 | 1.326 |
|  |  |  |  |  |  |  |  |  |  |
