# Supplementary material for: Dual energy X-ray absorptiometry body composition reference values of limbs and trunk from NHANES 1999–2004 with additional visualization methods
Source: PLoS One. 2017 Mar 27;12(3):e0174180. doi: 10.1371/journal.pone.0174180 (PMC5367711; doi:10.1371/journal.pone.0174180)
Supplement: S21 Table — This table provides L, M, and S values to derive average leg FMI Z-scores for 3rd through 97th percentiles for Hispanic females ages 8–85. (DOCX) [file pone.0174180.s029.docx]

Table S21: LMS Curve Fit Data providing L, M, and S values for 3^rd^ through 97^th^ percentiles for Hispanic Females Ages 8-85 for Average Leg FMI.

|  | Females | | | | | | | | |
| --- | --- | --- | --- | --- | --- | --- | --- | --- | --- |
|  |  |  | M | | | | | | |
| Age | L | S | 3 | 5 | 25 | 50 | 75 | 95 | 97 |
| 8 | -0.710 | 0.309 | 0.734 | 0.774 | 0.983 | 1.195 | 1.497 | 2.247 | 2.532 |
| 10 | -0.590 | 0.309 | 0.813 | 0.859 | 1.101 | 1.341 | 1.675 | 2.456 | 2.736 |
| 12 | -0.492 | 0.309 | 0.875 | 0.926 | 1.196 | 1.459 | 1.818 | 2.621 | 2.897 |
| 14 | -0.409 | 0.309 | 0.923 | 0.980 | 1.274 | 1.556 | 1.935 | 2.754 | 3.026 |
| 16 | -0.338 | 0.309 | 0.962 | 1.023 | 1.337 | 1.636 | 2.031 | 2.860 | 3.128 |
| 18 | -0.274 | 0.309 | 0.992 | 1.057 | 1.389 | 1.702 | 2.109 | 2.944 | 3.209 |
| 20 | -0.218 | 0.309 | 1.015 | 1.083 | 1.432 | 1.756 | 2.173 | 3.011 | 3.271 |
| 25 | -0.098 | 0.309 | 1.052 | 1.127 | 1.507 | 1.852 | 2.287 | 3.122 | 3.373 |
| 30 | 0.000 | 0.309 | 1.069 | 1.150 | 1.553 | 1.913 | 2.357 | 3.183 | 3.424 |
| 35 | 0.083 | 0.309 | 1.075 | 1.160 | 1.581 | 1.952 | 2.400 | 3.213 | 3.446 |
| 40 | 0.155 | 0.309 | 1.074 | 1.163 | 1.599 | 1.977 | 2.427 | 3.226 | 3.451 |
| 45 | 0.218 | 0.309 | 1.069 | 1.162 | 1.609 | 1.992 | 2.443 | 3.228 | 3.446 |
| 50 | 0.275 | 0.309 | 1.062 | 1.157 | 1.615 | 2.002 | 2.452 | 3.224 | 3.435 |
| 55 | 0.326 | 0.309 | 1.053 | 1.150 | 1.617 | 2.007 | 2.456 | 3.215 | 3.420 |
| 60 | 0.373 | 0.309 | 1.043 | 1.143 | 1.618 | 2.010 | 2.458 | 3.204 | 3.404 |
| 65 | 0.416 | 0.309 | 1.033 | 1.136 | 1.618 | 2.012 | 2.457 | 3.193 | 3.388 |
| 70 | 0.456 | 0.309 | 1.023 | 1.128 | 1.617 | 2.013 | 2.456 | 3.181 | 3.373 |
| 75 | 0.493 | 0.309 | 1.014 | 1.120 | 1.615 | 2.013 | 2.455 | 3.171 | 3.358 |
| 80 | 0.527 | 0.309 | 1.005 | 1.113 | 1.614 | 2.013 | 2.454 | 3.161 | 3.345 |
| 85 | 0.560 | 0.309 | 0.996 | 1.106 | 1.613 | 2.014 | 2.453 | 3.151 | 3.333 |
|  |  |  |  |  |  |  |  |  |  |
