# Supplementary material for: Dual energy X-ray absorptiometry body composition reference values of limbs and trunk from NHANES 1999–2004 with additional visualization methods
Source: PLoS One. 2017 Mar 27;12(3):e0174180. doi: 10.1371/journal.pone.0174180 (PMC5367711; doi:10.1371/journal.pone.0174180)
Supplement: S22 Table — This table provides L, M, and S values to derive average leg FMI Z-scores for 3rd through 97th percentiles for Hispanic males ages 8–85. (DOCX) [file pone.0174180.s030.docx]

Table S22: LMS Curve Fit Data providing L, M, and S values for 3^rd^ through 97^th^ percentiles for Hispanic Males Ages 8-85 for Average Leg FMI.

|  | Males | | | | | | | | |
| --- | --- | --- | --- | --- | --- | --- | --- | --- | --- |
|  |  |  | M | | | | | | |
| Age | L | S | 3 | 5 | 25 | 50 | 75 | 95 | 97 |
| 8 | -0.051 | 0.502 | 0.446 | 0.499 | 0.802 | 1.121 | 1.578 | 2.608 | 2.953 |
| 10 | -0.051 | 0.478 | 0.465 | 0.519 | 0.815 | 1.121 | 1.552 | 2.502 | 2.815 |
| 12 | -0.051 | 0.458 | 0.482 | 0.535 | 0.825 | 1.121 | 1.531 | 2.418 | 2.707 |
| 14 | -0.051 | 0.441 | 0.497 | 0.550 | 0.835 | 1.121 | 1.513 | 2.350 | 2.619 |
| 16 | -0.051 | 0.427 | 0.510 | 0.562 | 0.843 | 1.121 | 1.498 | 2.292 | 2.546 |
| 18 | -0.051 | 0.414 | 0.522 | 0.574 | 0.850 | 1.121 | 1.485 | 2.243 | 2.482 |
| 20 | -0.051 | 0.403 | 0.533 | 0.585 | 0.857 | 1.121 | 1.474 | 2.200 | 2.427 |
| 25 | -0.051 | 0.378 | 0.557 | 0.608 | 0.870 | 1.121 | 1.450 | 2.111 | 2.315 |
| 30 | -0.051 | 0.358 | 0.578 | 0.627 | 0.882 | 1.121 | 1.430 | 2.041 | 2.227 |
| 35 | -0.051 | 0.342 | 0.596 | 0.644 | 0.892 | 1.121 | 1.414 | 1.984 | 2.156 |
| 40 | -0.051 | 0.327 | 0.612 | 0.659 | 0.901 | 1.121 | 1.400 | 1.935 | 2.096 |
| 45 | -0.051 | 0.314 | 0.626 | 0.673 | 0.908 | 1.121 | 1.388 | 1.894 | 2.044 |
| 50 | -0.051 | 0.303 | 0.639 | 0.686 | 0.915 | 1.121 | 1.377 | 1.858 | 1.999 |
| 55 | -0.051 | 0.293 | 0.652 | 0.697 | 0.922 | 1.121 | 1.367 | 1.825 | 1.960 |
| 60 | -0.051 | 0.283 | 0.663 | 0.708 | 0.927 | 1.121 | 1.358 | 1.797 | 1.924 |
| 65 | -0.051 | 0.274 | 0.674 | 0.718 | 0.933 | 1.121 | 1.350 | 1.770 | 1.892 |
| 70 | -0.051 | 0.266 | 0.684 | 0.727 | 0.938 | 1.121 | 1.343 | 1.747 | 1.863 |
| 75 | -0.051 | 0.259 | 0.693 | 0.736 | 0.943 | 1.121 | 1.336 | 1.725 | 1.836 |
| 80 | -0.051 | 0.252 | 0.702 | 0.744 | 0.947 | 1.121 | 1.330 | 1.704 | 1.811 |
| 85 | -0.051 | 0.245 | 0.711 | 0.752 | 0.951 | 1.121 | 1.324 | 1.686 | 1.788 |
|  |  |  |  |  |  |  |  |  |  |
