# Supplementary material for: Dual energy X-ray absorptiometry body composition reference values of limbs and trunk from NHANES 1999–2004 with additional visualization methods
Source: PLoS One. 2017 Mar 27;12(3):e0174180. doi: 10.1371/journal.pone.0174180 (PMC5367711; doi:10.1371/journal.pone.0174180)
Supplement: S23 Table — This table provides L, M, and S values to derive average leg LMI Z-scores for 3rd through 97th percentiles for Hispanic males ages 8–85. (DOCX) [file pone.0174180.s031.docx]

Table S23: LMS Curve Fit Data providing L, M, and S values for 3^rd^ through 97^th^ percentiles for Hispanic Females Ages 8-85 for Average Leg LMI.

|  | Females | | | | | | | | |
| --- | --- | --- | --- | --- | --- | --- | --- | --- | --- |
|  |  |  | M | | | | | | |
|  |  |  | 3 | 5 | 25 | 50 | 75 | 95 | 97 |
| Age | L | S | -1.881 | -1.645 | -0.674 | 0 | 0.674 | 1.645 | 1.881 |
| 8 | -1.357 | 0.156 | 1.526 | 1.568 | 1.772 | 1.955 | 2.191 | 2.683 | 2.847 |
| 10 | -1.197 | 0.156 | 1.661 | 1.708 | 1.936 | 2.138 | 2.393 | 2.908 | 3.073 |
| 12 | -1.067 | 0.156 | 1.761 | 1.812 | 2.059 | 2.275 | 2.545 | 3.074 | 3.239 |
| 14 | -0.956 | 0.156 | 1.831 | 1.885 | 2.146 | 2.373 | 2.652 | 3.189 | 3.353 |
| 16 | -0.861 | 0.156 | 1.879 | 1.936 | 2.208 | 2.443 | 2.728 | 3.267 | 3.430 |
| 18 | -0.776 | 0.156 | 1.913 | 1.972 | 2.253 | 2.493 | 2.783 | 3.323 | 3.483 |
| 20 | -0.701 | 0.156 | 1.937 | 1.998 | 2.287 | 2.531 | 2.825 | 3.362 | 3.520 |
| 25 | -0.541 | 0.156 | 1.970 | 2.035 | 2.337 | 2.589 | 2.886 | 3.416 | 3.568 |
| 30 | -0.411 | 0.156 | 1.980 | 2.047 | 2.358 | 2.614 | 2.912 | 3.431 | 3.577 |
| 35 | -0.301 | 0.156 | 1.975 | 2.043 | 2.360 | 2.618 | 2.915 | 3.423 | 3.564 |
| 40 | -0.205 | 0.156 | 1.960 | 2.030 | 2.350 | 2.608 | 2.902 | 3.398 | 3.534 |
| 45 | -0.121 | 0.156 | 1.937 | 2.008 | 2.330 | 2.587 | 2.877 | 3.360 | 3.491 |
| 50 | -0.046 | 0.156 | 1.909 | 1.980 | 2.302 | 2.557 | 2.843 | 3.313 | 3.440 |
| 55 | 0.023 | 0.156 | 1.877 | 1.949 | 2.270 | 2.522 | 2.803 | 3.260 | 3.382 |
| 60 | 0.085 | 0.156 | 1.844 | 1.916 | 2.235 | 2.485 | 2.760 | 3.206 | 3.323 |
| 65 | 0.142 | 0.156 | 1.812 | 1.883 | 2.200 | 2.447 | 2.717 | 3.151 | 3.265 |
| 70 | 0.195 | 0.156 | 1.780 | 1.851 | 2.166 | 2.410 | 2.675 | 3.098 | 3.209 |
| 75 | 0.245 | 0.156 | 1.750 | 1.820 | 2.134 | 2.375 | 2.635 | 3.048 | 3.155 |
| 80 | 0.291 | 0.156 | 1.721 | 1.791 | 2.103 | 2.341 | 2.597 | 3.000 | 3.105 |
| 85 | 0.334 | 0.156 | 1.694 | 1.764 | 2.074 | 2.309 | 2.561 | 2.956 | 3.058 |
