# Supplementary material for: Dual energy X-ray absorptiometry body composition reference values of limbs and trunk from NHANES 1999–2004 with additional visualization methods
Source: PLoS One. 2017 Mar 27;12(3):e0174180. doi: 10.1371/journal.pone.0174180 (PMC5367711; doi:10.1371/journal.pone.0174180)
Supplement: S24 Table — This table provides L, M, and S values to derive average leg LMI Z-scores for 3rd through 97th percentiles for Hispanic males ages 8–85. (DOCX) [file pone.0174180.s032.docx]

Table S24: LMS Curve Fit Data providing L, M, and S values for 3^rd^ through 97^th^ percentiles for Hispanic Males Ages 8-85 for Average Leg LMI.

|  | Males | | | | | | | | |
| --- | --- | --- | --- | --- | --- | --- | --- | --- | --- |
|  |  |  | M | | | | | | |
|  |  |  | 3 | 5 | 25 | 50 | 75 | 95 | 97 |
| Age | L | S | -1.881 | -1.645 | -0.674 | 0 | 0.674 | 1.645 | 1.881 |
| 8 | 0.120 | 0.155 | 1.502 | 1.560 | 1.819 | 2.020 | 2.241 | 2.596 | 2.689 |
| 10 | 0.120 | 0.150 | 1.752 | 1.817 | 2.109 | 2.334 | 2.581 | 2.977 | 3.081 |
| 12 | 0.120 | 0.146 | 1.976 | 2.048 | 2.368 | 2.615 | 2.884 | 3.315 | 3.427 |
| 14 | 0.120 | 0.143 | 2.163 | 2.240 | 2.581 | 2.844 | 3.130 | 3.587 | 3.706 |
| 16 | 0.120 | 0.140 | 2.288 | 2.367 | 2.720 | 2.992 | 3.287 | 3.757 | 3.879 |
| 18 | 0.120 | 0.138 | 2.358 | 2.438 | 2.794 | 3.068 | 3.365 | 3.837 | 3.961 |
| 20 | 0.120 | 0.136 | 2.397 | 2.478 | 2.834 | 3.107 | 3.402 | 3.872 | 3.994 |
| 25 | 0.120 | 0.131 | 2.453 | 2.532 | 2.882 | 3.150 | 3.439 | 3.897 | 4.016 |
| 30 | 0.120 | 0.127 | 2.487 | 2.565 | 2.909 | 3.171 | 3.453 | 3.899 | 4.015 |
| 35 | 0.120 | 0.124 | 2.510 | 2.587 | 2.924 | 3.181 | 3.457 | 3.891 | 4.004 |
| 40 | 0.120 | 0.121 | 2.520 | 2.595 | 2.925 | 3.176 | 3.445 | 3.868 | 3.978 |
| 45 | 0.120 | 0.119 | 2.516 | 2.589 | 2.912 | 3.156 | 3.418 | 3.828 | 3.934 |
| 50 | 0.120 | 0.117 | 2.500 | 2.571 | 2.885 | 3.122 | 3.376 | 3.774 | 3.877 |
| 55 | 0.120 | 0.115 | 2.473 | 2.543 | 2.848 | 3.077 | 3.324 | 3.708 | 3.808 |
| 60 | 0.120 | 0.113 | 2.439 | 2.507 | 2.802 | 3.025 | 3.263 | 3.634 | 3.730 |
| 65 | 0.120 | 0.111 | 2.400 | 2.465 | 2.751 | 2.966 | 3.196 | 3.554 | 3.646 |
| 70 | 0.120 | 0.110 | 2.355 | 2.418 | 2.694 | 2.902 | 3.123 | 3.469 | 3.557 |
| 75 | 0.120 | 0.108 | 2.307 | 2.368 | 2.635 | 2.835 | 3.049 | 3.382 | 3.467 |
| 80 | 0.120 | 0.107 | 2.260 | 2.319 | 2.576 | 2.770 | 2.976 | 3.296 | 3.378 |
| 85 | 0.120 | 0.106 | 2.214 | 2.271 | 2.520 | 2.707 | 2.906 | 3.215 | 3.294 |
