# Supplementary material for: Dual energy X-ray absorptiometry body composition reference values of limbs and trunk from NHANES 1999–2004 with additional visualization methods
Source: PLoS One. 2017 Mar 27;12(3):e0174180. doi: 10.1371/journal.pone.0174180 (PMC5367711; doi:10.1371/journal.pone.0174180)
Supplement: S25 Table — This table provides L, M, and S values to derive trunk FMI Z-scores for 3rd through 97th percentiles for Hispanic females ages 8–85. (DOCX) [file pone.0174180.s033.docx]

Table S25: LMS Curve Fit Data providing L, M, and S values for 3^rd^ through 97^th^ percentiles for Hispanic Females Ages 8-85 for Trunk FMI.

|  | Females | | | | | | | | |
| --- | --- | --- | --- | --- | --- | --- | --- | --- | --- |
|  |  |  | M | | | | | | |
|  |  |  | 3 | 5 | 25 | 50 | 75 | 95 | 97 |
| Age | L | S | -1.881 | -1.645 | -0.674 | 0 | 0.674 | 1.645 | 1.881 |
| 8 | -0.432 | 0.518 | 0.841 | 0.919 | 1.371 | 1.899 | 2.773 | 5.498 | 6.729 |
| 10 | -0.343 | 0.500 | 1.040 | 1.138 | 1.708 | 2.349 | 3.360 | 6.172 | 7.309 |
| 12 | -0.266 | 0.484 | 1.218 | 1.337 | 2.013 | 2.752 | 3.871 | 6.732 | 7.804 |
| 14 | -0.198 | 0.470 | 1.385 | 1.524 | 2.300 | 3.127 | 4.336 | 7.231 | 8.256 |
| 16 | -0.136 | 0.457 | 1.542 | 1.700 | 2.571 | 3.476 | 4.760 | 7.677 | 8.665 |
| 18 | -0.079 | 0.445 | 1.689 | 1.864 | 2.823 | 3.797 | 5.143 | 8.069 | 9.025 |
| 20 | -0.027 | 0.434 | 1.823 | 2.015 | 3.054 | 4.087 | 5.481 | 8.402 | 9.329 |
| 25 | 0.090 | 0.410 | 2.107 | 2.337 | 3.541 | 4.683 | 6.151 | 9.009 | 9.866 |
| 30 | 0.191 | 0.389 | 2.339 | 2.600 | 3.929 | 5.140 | 6.636 | 9.394 | 10.188 |
| 35 | 0.281 | 0.370 | 2.536 | 2.821 | 4.246 | 5.498 | 6.995 | 9.640 | 10.378 |
| 40 | 0.362 | 0.353 | 2.705 | 3.012 | 4.506 | 5.778 | 7.259 | 9.785 | 10.473 |
| 45 | 0.437 | 0.337 | 2.850 | 3.174 | 4.715 | 5.991 | 7.441 | 9.846 | 10.488 |
| 50 | 0.506 | 0.323 | 2.971 | 3.307 | 4.874 | 6.138 | 7.546 | 9.826 | 10.425 |
| 55 | 0.570 | 0.309 | 3.070 | 3.414 | 4.988 | 6.228 | 7.585 | 9.739 | 10.297 |
| 60 | 0.630 | 0.297 | 3.152 | 3.500 | 5.066 | 6.274 | 7.576 | 9.605 | 10.125 |
| 65 | 0.687 | 0.285 | 3.221 | 3.570 | 5.116 | 6.287 | 7.531 | 9.440 | 9.924 |
| 70 | 0.741 | 0.274 | 3.281 | 3.628 | 5.147 | 6.277 | 7.462 | 9.258 | 9.709 |
| 75 | 0.792 | 0.263 | 3.335 | 3.680 | 5.164 | 6.252 | 7.380 | 9.071 | 9.492 |
| 80 | 0.841 | 0.253 | 3.388 | 3.728 | 5.175 | 6.220 | 7.295 | 8.887 | 9.281 |
| 85 | 0.888 | 0.243 | 3.441 | 3.775 | 5.183 | 6.188 | 7.211 | 8.713 | 9.083 |
