# Supplementary material for: Dual energy X-ray absorptiometry body composition reference values of limbs and trunk from NHANES 1999–2004 with additional visualization methods
Source: PLoS One. 2017 Mar 27;12(3):e0174180. doi: 10.1371/journal.pone.0174180 (PMC5367711; doi:10.1371/journal.pone.0174180)
Supplement: S26 Table — This table provides L, M, and S values to derive trunk FMI Z-scores for 3rd through 97th percentiles for Hispanic males ages 8–85. (DOCX) [file pone.0174180.s034.docx]

Table S26: LMS Curve Fit Data providing L, M, and S values for 3^rd^ through 97^th^ percentiles for Hispanic Males Ages 8-85 for Trunk FMI.

|  | Males | | | | | | | | |
| --- | --- | --- | --- | --- | --- | --- | --- | --- | --- |
|  |  |  | M | | | | | | |
|  |  |  | 3 | 5 | 25 | 50 | 75 | 95 | 97 |
| Age | L | S | -1.881 | -1.645 | -0.674 | 0 | 0.674 | 1.645 | 1.881 |
| 8 | -0.672 | 0.617 | 0.781 | 0.850 | 1.276 | 1.842 | 2.999 | 10.132 | 17.520 |
| 10 | -0.525 | 0.583 | 0.829 | 0.907 | 1.379 | 1.970 | 3.059 | 7.465 | 10.065 |
| 12 | -0.404 | 0.555 | 0.858 | 0.943 | 1.447 | 2.050 | 3.074 | 6.397 | 7.943 |
| 14 | -0.302 | 0.531 | 0.890 | 0.981 | 1.517 | 2.131 | 3.112 | 5.874 | 6.995 |
| 16 | -0.214 | 0.511 | 0.945 | 1.045 | 1.623 | 2.263 | 3.235 | 5.710 | 6.628 |
| 18 | -0.136 | 0.493 | 1.020 | 1.131 | 1.764 | 2.441 | 3.428 | 5.757 | 6.568 |
| 20 | -0.066 | 0.476 | 1.107 | 1.232 | 1.925 | 2.645 | 3.659 | 5.913 | 6.660 |
| 25 | 0.082 | 0.442 | 1.336 | 1.494 | 2.339 | 3.162 | 4.245 | 6.409 | 7.070 |
| 30 | 0.203 | 0.414 | 1.534 | 1.720 | 2.686 | 3.581 | 4.698 | 6.780 | 7.382 |
| 35 | 0.305 | 0.391 | 1.694 | 1.903 | 2.957 | 3.891 | 5.012 | 6.997 | 7.551 |
| 40 | 0.393 | 0.370 | 1.827 | 2.055 | 3.170 | 4.122 | 5.229 | 7.115 | 7.627 |
| 45 | 0.471 | 0.352 | 1.942 | 2.185 | 3.340 | 4.296 | 5.380 | 7.170 | 7.646 |
| 50 | 0.541 | 0.336 | 2.045 | 2.298 | 3.480 | 4.431 | 5.487 | 7.187 | 7.632 |
| 55 | 0.604 | 0.321 | 2.137 | 2.399 | 3.595 | 4.534 | 5.558 | 7.174 | 7.591 |
| 60 | 0.661 | 0.308 | 2.221 | 2.488 | 3.688 | 4.611 | 5.602 | 7.139 | 7.531 |
| 65 | 0.714 | 0.296 | 2.296 | 2.567 | 3.764 | 4.667 | 5.623 | 7.086 | 7.455 |
| 70 | 0.764 | 0.284 | 2.366 | 2.638 | 3.826 | 4.707 | 5.629 | 7.022 | 7.371 |
| 75 | 0.809 | 0.274 | 2.433 | 2.705 | 3.879 | 4.737 | 5.626 | 6.955 | 7.286 |
| 80 | 0.852 | 0.264 | 2.498 | 2.769 | 3.927 | 4.762 | 5.620 | 6.891 | 7.205 |
| 85 | 0.892 | 0.255 | 2.562 | 2.832 | 3.972 | 4.785 | 5.614 | 6.831 | 7.130 |
