# Supplementary material for: Dual energy X-ray absorptiometry body composition reference values of limbs and trunk from NHANES 1999–2004 with additional visualization methods
Source: PLoS One. 2017 Mar 27;12(3):e0174180. doi: 10.1371/journal.pone.0174180 (PMC5367711; doi:10.1371/journal.pone.0174180)
Supplement: S27 Table — This table provides L, M, and S values to derive trunk LMI Z-scores for 3rd through 97th percentiles for Hispanic females ages 8–85. (DOCX) [file pone.0174180.s035.docx]

Table S27: LMS Curve Fit Data providing L, M, and S values for 3^rd^ through 97^th^ percentiles for Hispanic Females Ages 8-85 for Trunk LMI.

|  | Females | | | | | | | | |
| --- | --- | --- | --- | --- | --- | --- | --- | --- | --- |
|  |  |  | M | | | | | | |
|  |  |  | 3 | 5 | 25 | 50 | 75 | 95 | 97 |
| Age | L | S | -1.881 | -1.645 | -0.674 | 0 | 0.674 | 1.645 | 1.881 |
| 8 | -1.970 | 0.129 | 4.444 | 4.539 | 5.001 | 5.419 | 5.960 | 7.131 | 7.535 |
| 10 | -1.769 | 0.129 | 4.952 | 5.061 | 5.589 | 6.060 | 6.659 | 7.905 | 8.318 |
| 12 | -1.604 | 0.129 | 5.355 | 5.476 | 6.059 | 6.573 | 7.218 | 8.518 | 8.936 |
| 14 | -1.465 | 0.129 | 5.666 | 5.797 | 6.425 | 6.972 | 7.651 | 8.988 | 9.408 |
| 16 | -1.345 | 0.129 | 5.900 | 6.039 | 6.704 | 7.278 | 7.983 | 9.343 | 9.761 |
| 18 | -1.239 | 0.129 | 6.080 | 6.226 | 6.921 | 7.517 | 8.240 | 9.613 | 10.029 |
| 20 | -1.144 | 0.129 | 6.221 | 6.373 | 7.093 | 7.705 | 8.444 | 9.824 | 10.236 |
| 25 | -0.942 | 0.129 | 6.456 | 6.619 | 7.388 | 8.031 | 8.794 | 10.175 | 10.577 |
| 30 | -0.778 | 0.129 | 6.590 | 6.763 | 7.566 | 8.229 | 9.004 | 10.376 | 10.767 |
| 35 | -0.639 | 0.129 | 6.666 | 6.846 | 7.675 | 8.352 | 9.133 | 10.490 | 10.870 |
| 40 | -0.518 | 0.129 | 6.704 | 6.889 | 7.738 | 8.424 | 9.208 | 10.547 | 10.917 |
| 45 | -0.412 | 0.129 | 6.712 | 6.902 | 7.766 | 8.458 | 9.241 | 10.560 | 10.920 |
| 50 | -0.317 | 0.129 | 6.697 | 6.890 | 7.765 | 8.460 | 9.239 | 10.537 | 10.888 |
| 55 | -0.231 | 0.129 | 6.665 | 6.861 | 7.743 | 8.439 | 9.214 | 10.489 | 10.832 |
| 60 | -0.153 | 0.129 | 6.624 | 6.821 | 7.710 | 8.405 | 9.173 | 10.427 | 10.761 |
| 65 | -0.081 | 0.129 | 6.577 | 6.777 | 7.669 | 8.363 | 9.125 | 10.357 | 10.683 |
| 70 | -0.014 | 0.129 | 6.529 | 6.730 | 7.625 | 8.317 | 9.073 | 10.285 | 10.604 |
| 75 | 0.048 | 0.129 | 6.481 | 6.683 | 7.581 | 8.271 | 9.020 | 10.214 | 10.526 |
| 80 | 0.107 | 0.129 | 6.434 | 6.638 | 7.539 | 8.227 | 8.970 | 10.146 | 10.452 |
| 85 | 0.161 | 0.129 | 6.391 | 6.596 | 7.499 | 8.184 | 8.922 | 10.082 | 10.382 |
