# Supplementary material for: Dual energy X-ray absorptiometry body composition reference values of limbs and trunk from NHANES 1999–2004 with additional visualization methods
Source: PLoS One. 2017 Mar 27;12(3):e0174180. doi: 10.1371/journal.pone.0174180 (PMC5367711; doi:10.1371/journal.pone.0174180)
Supplement: S28 Table — This table provides L, M, and S values to derive trunk LMI Z-scores for 3rd through 97th percentiles for Hispanic males ages 8–85. (DOCX) [file pone.0174180.s036.docx]

Table S28: LMS Curve Fit Data providing L, M, and S values for 3^rd^ through 97^th^ percentiles for Hispanic Males Ages 8-85 for Trunk LMI.

|  | Males | | | | | | | | |
| --- | --- | --- | --- | --- | --- | --- | --- | --- | --- |
|  |  |  | M | | | | | | |
|  |  |  | 3 | 5 | 25 | 50 | 75 | 95 | 97 |
| Age | L | S | -1.881 | -1.645 | -0.674 | 0 | 0.674 | 1.645 | 1.881 |
| 8 | -0.144 | 0.150 | 4.392 | 4.544 | 5.239 | 5.793 | 6.414 | 7.448 | 7.728 |
| 10 | -0.144 | 0.145 | 4.803 | 4.964 | 5.696 | 6.277 | 6.926 | 8.001 | 8.291 |
| 12 | -0.144 | 0.141 | 5.327 | 5.501 | 6.287 | 6.909 | 7.602 | 8.746 | 9.052 |
| 14 | -0.144 | 0.137 | 5.945 | 6.135 | 6.989 | 7.662 | 8.411 | 9.641 | 9.971 |
| 16 | -0.144 | 0.134 | 6.470 | 6.671 | 7.579 | 8.292 | 9.083 | 10.380 | 10.726 |
| 18 | -0.144 | 0.132 | 6.814 | 7.022 | 7.957 | 8.691 | 9.503 | 10.830 | 11.183 |
| 20 | -0.144 | 0.129 | 7.040 | 7.251 | 8.198 | 8.940 | 9.759 | 11.094 | 11.450 |
| 25 | -0.144 | 0.124 | 7.394 | 7.607 | 8.560 | 9.303 | 10.120 | 11.446 | 11.798 |
| 30 | -0.144 | 0.120 | 7.606 | 7.818 | 8.764 | 9.498 | 10.303 | 11.604 | 11.948 |
| 35 | -0.144 | 0.116 | 7.762 | 7.972 | 8.907 | 9.631 | 10.422 | 11.697 | 12.033 |
| 40 | -0.144 | 0.113 | 7.879 | 8.087 | 9.010 | 9.722 | 10.499 | 11.747 | 12.076 |
| 45 | -0.144 | 0.111 | 7.963 | 8.168 | 9.078 | 9.778 | 10.540 | 11.761 | 12.081 |
| 50 | -0.144 | 0.108 | 8.020 | 8.222 | 9.118 | 9.805 | 10.552 | 11.745 | 12.058 |
| 55 | -0.144 | 0.106 | 8.047 | 8.246 | 9.125 | 9.799 | 10.530 | 11.695 | 12.001 |
| 60 | -0.144 | 0.104 | 8.041 | 8.236 | 9.097 | 9.756 | 10.469 | 11.605 | 11.902 |
| 65 | -0.144 | 0.102 | 7.996 | 8.186 | 9.027 | 9.669 | 10.363 | 11.466 | 11.754 |
| 70 | -0.144 | 0.101 | 7.912 | 8.097 | 8.915 | 9.538 | 10.211 | 11.279 | 11.557 |
| 75 | -0.144 | 0.099 | 7.801 | 7.981 | 8.774 | 9.377 | 10.028 | 11.060 | 11.328 |
| 80 | -0.144 | 0.098 | 7.679 | 7.853 | 8.621 | 9.205 | 9.835 | 10.830 | 11.089 |
| 85 | -0.144 | 0.096 | 7.556 | 7.725 | 8.470 | 9.035 | 9.644 | 10.605 | 10.855 |
