# Supplementary material for: Dual energy X-ray absorptiometry body composition reference values of limbs and trunk from NHANES 1999–2004 with additional visualization methods
Source: PLoS One. 2017 Mar 27;12(3):e0174180. doi: 10.1371/journal.pone.0174180 (PMC5367711; doi:10.1371/journal.pone.0174180)
Supplement: S29 Table — This table provides L, M, and S values to derive total body FMI Z-scores for 3rd through 97th percentiles for Hispanic females ages 8–85. (DOCX) [file pone.0174180.s037.docx]

Table S29: LMS Curve Fit Data providing L, M, and S values for 3^rd^ through 97^th^ percentiles for Hispanic Females Ages 8-85 for Total Body FMI.

|  | Females | | | | | | | | |
| --- | --- | --- | --- | --- | --- | --- | --- | --- | --- |
|  |  |  | M | | | | | | |
| Age | L | S | 3 | 5 | 25 | 50 | 75 | 95 | 97 |
| 8 | -0.613 | 0.391 | 2.983 | 3.183 | 4.286 | 5.471 | 7.292 | 12.386 | 14.531 |
| 10 | -0.523 | 0.381 | 3.394 | 3.628 | 4.903 | 6.240 | 8.222 | 13.342 | 15.329 |
| 12 | -0.443 | 0.373 | 3.762 | 4.028 | 5.460 | 6.929 | 9.045 | 14.176 | 16.050 |
| 14 | -0.371 | 0.365 | 4.099 | 4.395 | 5.974 | 7.561 | 9.788 | 14.922 | 16.712 |
| 16 | -0.305 | 0.358 | 4.408 | 4.732 | 6.446 | 8.138 | 10.459 | 15.585 | 17.306 |
| 18 | -0.243 | 0.352 | 4.688 | 5.039 | 6.877 | 8.661 | 11.058 | 16.165 | 17.826 |
| 20 | -0.186 | 0.346 | 4.938 | 5.314 | 7.265 | 9.128 | 11.585 | 16.656 | 18.262 |
| 25 | -0.055 | 0.332 | 5.449 | 5.879 | 8.063 | 10.074 | 12.621 | 17.551 | 19.032 |
| 30 | 0.060 | 0.320 | 5.837 | 6.312 | 8.676 | 10.781 | 13.360 | 18.111 | 19.485 |
| 35 | 0.164 | 0.309 | 6.141 | 6.654 | 9.157 | 11.321 | 13.897 | 18.457 | 19.736 |
| 40 | 0.260 | 0.299 | 6.385 | 6.929 | 9.540 | 11.736 | 14.287 | 18.655 | 19.851 |
| 45 | 0.349 | 0.290 | 6.575 | 7.146 | 9.836 | 12.043 | 14.551 | 18.727 | 19.847 |
| 50 | 0.431 | 0.281 | 6.711 | 7.303 | 10.042 | 12.240 | 14.688 | 18.667 | 19.717 |
| 55 | 0.510 | 0.273 | 6.798 | 7.405 | 10.168 | 12.337 | 14.712 | 18.492 | 19.475 |
| 60 | 0.584 | 0.266 | 6.849 | 7.466 | 10.231 | 12.360 | 14.654 | 18.238 | 19.158 |
| 65 | 0.654 | 0.258 | 6.872 | 7.495 | 10.247 | 12.326 | 14.534 | 17.930 | 18.792 |
| 70 | 0.721 | 0.251 | 6.876 | 7.502 | 10.228 | 12.251 | 14.373 | 17.588 | 18.396 |
| 75 | 0.786 | 0.244 | 6.870 | 7.496 | 10.187 | 12.153 | 14.189 | 17.235 | 17.994 |
| 80 | 0.847 | 0.238 | 6.861 | 7.486 | 10.137 | 12.044 | 13.998 | 16.887 | 17.601 |
| 85 | 0.907 | 0.232 | 6.853 | 7.476 | 10.084 | 11.933 | 13.810 | 16.555 | 17.229 |
|  |  |  |  |  |  |  |  |  |  |
