# Supplementary material for: Dual energy X-ray absorptiometry body composition reference values of limbs and trunk from NHANES 1999–2004 with additional visualization methods
Source: PLoS One. 2017 Mar 27;12(3):e0174180. doi: 10.1371/journal.pone.0174180 (PMC5367711; doi:10.1371/journal.pone.0174180)
Supplement: S30 Table — This table provides L, M, and S values to derive total body FMI Z-scores for 3rd through 97th percentiles for Hispanic males ages 8–85. (DOCX) [file pone.0174180.s038.docx]

Table S30: LMS Curve Fit Data providing L, M, and S values for 3^rd^ through 97^th^ percentiles for Hispanic Males Ages 8-85 for Total Body FMI.

|  | Males | | | | | | | | |
| --- | --- | --- | --- | --- | --- | --- | --- | --- | --- |
|  |  |  | M | | | | | | |
| Age | L | S | 3 | 5 | 25 | 50 | 75 | 95 | 97 |
| 8 | -0.611 | 0.514 | 2.477 | 2.679 | 3.869 | 5.299 | 7.822 | 17.426 | 22.885 |
| 10 | -0.487 | 0.487 | 2.524 | 2.738 | 3.972 | 5.388 | 7.706 | 14.886 | 18.142 |
| 12 | -0.385 | 0.465 | 2.562 | 2.785 | 4.051 | 5.447 | 7.610 | 13.495 | 15.847 |
| 14 | -0.300 | 0.447 | 2.606 | 2.839 | 4.134 | 5.516 | 7.563 | 12.652 | 14.525 |
| 16 | -0.225 | 0.431 | 2.676 | 2.919 | 4.251 | 5.632 | 7.604 | 12.188 | 13.778 |
| 18 | -0.160 | 0.417 | 2.771 | 3.026 | 4.405 | 5.797 | 7.726 | 11.979 | 13.390 |
| 20 | -0.101 | 0.404 | 2.884 | 3.153 | 4.584 | 5.996 | 7.902 | 11.926 | 13.217 |
| 25 | 0.023 | 0.377 | 3.192 | 3.494 | 5.056 | 6.524 | 8.404 | 12.075 | 13.181 |
| 30 | 0.124 | 0.355 | 3.477 | 3.807 | 5.474 | 6.979 | 8.834 | 12.262 | 13.253 |
| 35 | 0.210 | 0.336 | 3.721 | 4.074 | 5.815 | 7.335 | 9.153 | 12.380 | 13.285 |
| 40 | 0.284 | 0.320 | 3.931 | 4.302 | 6.091 | 7.610 | 9.384 | 12.433 | 13.270 |
| 45 | 0.349 | 0.306 | 4.116 | 4.499 | 6.318 | 7.826 | 9.550 | 12.442 | 13.222 |
| 50 | 0.408 | 0.293 | 4.281 | 4.673 | 6.508 | 7.997 | 9.672 | 12.423 | 13.154 |
| 55 | 0.461 | 0.282 | 4.430 | 4.829 | 6.668 | 8.135 | 9.759 | 12.382 | 13.071 |
| 60 | 0.509 | 0.271 | 4.567 | 4.970 | 6.806 | 8.246 | 9.821 | 12.327 | 12.979 |
| 65 | 0.553 | 0.262 | 4.693 | 5.098 | 6.924 | 8.336 | 9.863 | 12.263 | 12.881 |
| 70 | 0.595 | 0.253 | 4.811 | 5.217 | 7.028 | 8.410 | 9.892 | 12.194 | 12.783 |
| 75 | 0.633 | 0.244 | 4.923 | 5.329 | 7.122 | 8.475 | 9.913 | 12.125 | 12.687 |
| 80 | 0.669 | 0.237 | 5.032 | 5.437 | 7.209 | 8.534 | 9.930 | 12.060 | 12.598 |
| 85 | 0.703 | 0.229 | 5.137 | 5.541 | 7.292 | 8.588 | 9.945 | 12.000 | 12.516 |
|  |  |  |  |  |  |  |  |  |  |
