# Supplementary material for: Dual energy X-ray absorptiometry body composition reference values of limbs and trunk from NHANES 1999–2004 with additional visualization methods
Source: PLoS One. 2017 Mar 27;12(3):e0174180. doi: 10.1371/journal.pone.0174180 (PMC5367711; doi:10.1371/journal.pone.0174180)
Supplement: S31 Table — This table provides L, M, and S values to derive total body LMI Z-scores for 3rd through 97th percentiles for Hispanic females ages 8–85. (DOCX) [file pone.0174180.s039.docx]

Table S31: LMS Curve Fit Data providing L, M, and S values for 3^rd^ through 97^th^ percentiles for Hispanic Females Ages 8-85 for Total Body LMI.

|  | Females | | | | | | | | |
| --- | --- | --- | --- | --- | --- | --- | --- | --- | --- |
|  |  |  | M | | | | | | |
| Age | L | S | 3 | 5 | 25 | 50 | 75 | 95 | 97 |
| 8 | -2.075 | 0.126 | 9.866 | 10.069 | 11.062 | 11.962 | 13.131 | 15.678 | 16.566 |
| 10 | -1.876 | 0.126 | 10.726 | 10.954 | 12.060 | 13.047 | 14.310 | 16.957 | 17.843 |
| 12 | -1.707 | 0.126 | 11.431 | 11.680 | 12.884 | 13.946 | 15.284 | 18.004 | 18.886 |
| 14 | -1.559 | 0.126 | 11.940 | 12.207 | 13.487 | 14.606 | 15.997 | 18.754 | 19.625 |
| 16 | -1.427 | 0.126 | 12.289 | 12.569 | 13.910 | 15.070 | 16.496 | 19.261 | 20.117 |
| 18 | -1.307 | 0.126 | 12.552 | 12.844 | 14.235 | 15.427 | 16.879 | 19.640 | 20.479 |
| 20 | -1.198 | 0.126 | 12.759 | 13.062 | 14.495 | 15.715 | 17.186 | 19.937 | 20.760 |
| 25 | -0.959 | 0.126 | 13.087 | 13.411 | 14.931 | 16.199 | 17.698 | 20.405 | 21.190 |
| 30 | -0.756 | 0.126 | 13.272 | 13.614 | 15.199 | 16.502 | 18.014 | 20.669 | 21.420 |
| 35 | -0.578 | 0.126 | 13.401 | 13.758 | 15.401 | 16.730 | 18.251 | 20.858 | 21.581 |
| 40 | -0.419 | 0.126 | 13.488 | 13.860 | 15.552 | 16.904 | 18.429 | 20.992 | 21.691 |
| 45 | -0.276 | 0.126 | 13.517 | 13.901 | 15.634 | 17.001 | 18.525 | 21.041 | 21.717 |
| 50 | -0.145 | 0.126 | 13.449 | 13.842 | 15.601 | 16.974 | 18.486 | 20.945 | 21.598 |
| 55 | -0.024 | 0.126 | 13.297 | 13.696 | 15.469 | 16.837 | 18.330 | 20.721 | 21.349 |
| 60 | 0.089 | 0.126 | 13.096 | 13.499 | 15.278 | 16.636 | 18.103 | 20.424 | 21.028 |
| 65 | 0.194 | 0.126 | 12.863 | 13.268 | 15.046 | 16.390 | 17.829 | 20.079 | 20.659 |
| 70 | 0.293 | 0.126 | 12.608 | 13.015 | 14.787 | 16.114 | 17.522 | 19.701 | 20.258 |
| 75 | 0.386 | 0.126 | 12.345 | 12.752 | 14.516 | 15.824 | 17.201 | 19.311 | 19.847 |
| 80 | 0.475 | 0.126 | 12.085 | 12.493 | 14.246 | 15.535 | 16.882 | 18.927 | 19.442 |
| 85 | 0.559 | 0.126 | 11.834 | 12.242 | 13.986 | 15.255 | 16.574 | 18.557 | 19.054 |
|  |  |  |  |  |  |  |  |  |  |
