# Supplementary material for: Dual energy X-ray absorptiometry body composition reference values of limbs and trunk from NHANES 1999–2004 with additional visualization methods
Source: PLoS One. 2017 Mar 27;12(3):e0174180. doi: 10.1371/journal.pone.0174180 (PMC5367711; doi:10.1371/journal.pone.0174180)
Supplement: S32 Table — This table provides L, M, and S values to derive total body LMI Z-scores for 3rd through 97th percentiles for Hispanic males ages 8–85. (DOCX) [file pone.0174180.s040.docx]

Table S32: LMS Curve Fit Data providing L, M, and S values for 3^rd^ through 97^th^ percentiles for Hispanic Males Ages 8-85 for Total Body LMI.

|  | Males | | | | | | | | |
| --- | --- | --- | --- | --- | --- | --- | --- | --- | --- |
|  |  |  | M | | | | | | |
| Age | L | S | 3 | 5 | 25 | 50 | 75 | 95 | 97 |
| 8 | -0.093 | 0.138 | 9.955 | 10.276 | 11.722 | 12.855 | 14.109 | 16.158 | 16.703 |
| 10 | -0.093 | 0.133 | 10.864 | 11.203 | 12.725 | 13.914 | 15.225 | 17.357 | 17.924 |
| 12 | -0.093 | 0.129 | 11.953 | 12.316 | 13.941 | 15.206 | 16.597 | 18.853 | 19.451 |
| 14 | -0.093 | 0.126 | 13.152 | 13.542 | 15.284 | 16.637 | 18.121 | 20.521 | 21.155 |
| 16 | -0.093 | 0.124 | 14.115 | 14.524 | 16.351 | 17.766 | 19.316 | 21.815 | 22.475 |
| 18 | -0.093 | 0.121 | 14.715 | 15.134 | 17.000 | 18.442 | 20.019 | 22.555 | 23.224 |
| 20 | -0.093 | 0.119 | 15.100 | 15.522 | 17.400 | 18.850 | 20.432 | 22.973 | 23.641 |
| 25 | -0.093 | 0.115 | 15.717 | 16.140 | 18.017 | 19.459 | 21.029 | 23.539 | 24.197 |
| 30 | -0.093 | 0.111 | 16.092 | 16.512 | 18.368 | 19.790 | 21.333 | 23.792 | 24.436 |
| 35 | -0.093 | 0.108 | 16.348 | 16.762 | 18.592 | 19.990 | 21.503 | 23.908 | 24.536 |
| 40 | -0.093 | 0.105 | 16.507 | 16.915 | 18.714 | 20.085 | 21.566 | 23.915 | 24.527 |
| 45 | -0.093 | 0.103 | 16.584 | 16.985 | 18.749 | 20.090 | 21.538 | 23.827 | 24.423 |
| 50 | -0.093 | 0.101 | 16.597 | 16.990 | 18.717 | 20.028 | 21.440 | 23.669 | 24.249 |
| 55 | -0.093 | 0.099 | 16.546 | 16.930 | 18.617 | 19.895 | 21.270 | 23.438 | 24.000 |
| 60 | -0.093 | 0.097 | 16.434 | 16.809 | 18.453 | 19.697 | 21.033 | 23.136 | 23.682 |
| 65 | -0.093 | 0.096 | 16.256 | 16.621 | 18.219 | 19.426 | 20.722 | 22.757 | 23.284 |
| 70 | -0.093 | 0.094 | 16.008 | 16.362 | 17.909 | 19.077 | 20.329 | 22.293 | 22.801 |
| 75 | -0.093 | 0.093 | 15.713 | 16.055 | 17.550 | 18.678 | 19.885 | 21.776 | 22.265 |
| 80 | -0.093 | 0.091 | 15.399 | 15.729 | 17.173 | 18.261 | 19.424 | 21.244 | 21.714 |
| 85 | -0.093 | 0.090 | 15.089 | 15.408 | 16.803 | 17.852 | 18.974 | 20.727 | 21.179 |
|  |  |  |  |  |  |  |  |  |  |
