# Supplementary material for: Dual energy X-ray absorptiometry body composition reference values of limbs and trunk from NHANES 1999–2004 with additional visualization methods
Source: PLoS One. 2017 Mar 27;12(3):e0174180. doi: 10.1371/journal.pone.0174180 (PMC5367711; doi:10.1371/journal.pone.0174180)
Supplement: S33 Table — This table provides L, M, and S values to derive average arm FMI Z-scores for 3rd through 97th percentiles for white females ages 8–85. (DOCX) [file pone.0174180.s041.docx]

Table S33: LMS Curve Fit Data providing L, M, and S values for 3^rd^ through 97^th^ percentiles for White Females Ages 8-85 for Average Arm FMI.

|  | Females | | | | | | | | |
| --- | --- | --- | --- | --- | --- | --- | --- | --- | --- |
|  |  |  | M | | | | | | |
|  |  |  | 3 | 5 | 25 | 50 | 75 | 95 | 97 |
| Age | L | S | -1.881 | -1.645 | -0.674 | 0.000 | 0.674 | 1.645 | 1.881 |
| 8 | -0.468 | 0.491 | 0.154 | 0.167 | 0.243 | 0.331 | 0.474 | 0.913 | 1.109 |
| 10 | -0.380 | 0.478 | 0.169 | 0.185 | 0.271 | 0.367 | 0.518 | 0.935 | 1.104 |
| 12 | -0.308 | 0.468 | 0.182 | 0.199 | 0.294 | 0.397 | 0.553 | 0.956 | 1.108 |
| 14 | -0.248 | 0.459 | 0.193 | 0.211 | 0.313 | 0.422 | 0.582 | 0.974 | 1.115 |
| 16 | -0.195 | 0.452 | 0.202 | 0.222 | 0.330 | 0.443 | 0.607 | 0.990 | 1.123 |
| 18 | -0.149 | 0.445 | 0.210 | 0.231 | 0.345 | 0.463 | 0.629 | 1.004 | 1.131 |
| 20 | -0.108 | 0.439 | 0.217 | 0.239 | 0.358 | 0.480 | 0.648 | 1.017 | 1.139 |
| 25 | -0.020 | 0.426 | 0.233 | 0.257 | 0.387 | 0.516 | 0.688 | 1.045 | 1.158 |
| 30 | 0.051 | 0.416 | 0.245 | 0.272 | 0.411 | 0.545 | 0.720 | 1.068 | 1.175 |
| 35 | 0.112 | 0.407 | 0.256 | 0.284 | 0.431 | 0.570 | 0.747 | 1.088 | 1.189 |
| 40 | 0.164 | 0.400 | 0.265 | 0.295 | 0.449 | 0.592 | 0.770 | 1.105 | 1.203 |
| 45 | 0.210 | 0.393 | 0.274 | 0.305 | 0.465 | 0.611 | 0.791 | 1.120 | 1.215 |
| 50 | 0.252 | 0.387 | 0.281 | 0.314 | 0.479 | 0.628 | 0.809 | 1.134 | 1.226 |
| 55 | 0.289 | 0.382 | 0.288 | 0.322 | 0.493 | 0.643 | 0.825 | 1.146 | 1.236 |
| 60 | 0.323 | 0.377 | 0.294 | 0.329 | 0.504 | 0.658 | 0.839 | 1.157 | 1.245 |
| 65 | 0.355 | 0.372 | 0.300 | 0.336 | 0.516 | 0.671 | 0.853 | 1.167 | 1.253 |
| 70 | 0.384 | 0.368 | 0.305 | 0.343 | 0.526 | 0.683 | 0.865 | 1.176 | 1.261 |
| 75 | 0.411 | 0.364 | 0.310 | 0.349 | 0.536 | 0.694 | 0.876 | 1.185 | 1.269 |
| 80 | 0.436 | 0.361 | 0.315 | 0.355 | 0.545 | 0.704 | 0.887 | 1.193 | 1.276 |
| 85 | 0.460 | 0.357 | 0.320 | 0.360 | 0.553 | 0.714 | 0.897 | 1.201 | 1.282 |
