# Supplementary material for: Dual energy X-ray absorptiometry body composition reference values of limbs and trunk from NHANES 1999–2004 with additional visualization methods
Source: PLoS One. 2017 Mar 27;12(3):e0174180. doi: 10.1371/journal.pone.0174180 (PMC5367711; doi:10.1371/journal.pone.0174180)
Supplement: S34 Table — This table provides L, M, and S values to derive average arm FMI Z-scores for 3rd through 97th percentiles for white males ages 8–85. (DOCX) [file pone.0174180.s042.docx]

Table S34: LMS Curve Fit Data providing L, M, and S values for 3^rd^ through 97^th^ percentiles for White Males Ages 8-85 for Average Arm FMI.

|  | Males | | | | | | | | |
| --- | --- | --- | --- | --- | --- | --- | --- | --- | --- |
|  |  |  | M | | | | | | |
|  |  |  | 3 | 5 | 25 | 50 | 75 | 95 | 97 |
| Age | L | S | -1.881 | -1.645 | -0.674 | 0.000 | 0.674 | 1.645 | 1.881 |
| 8 | -0.703 | 0.505 | 0.130 | 0.140 | 0.199 | 0.270 | 0.398 | 0.940 | 1.295 |
| 10 | -0.595 | 0.484 | 0.133 | 0.144 | 0.205 | 0.276 | 0.397 | 0.812 | 1.025 |
| 12 | -0.507 | 0.466 | 0.136 | 0.147 | 0.210 | 0.282 | 0.396 | 0.744 | 0.898 |
| 14 | -0.432 | 0.452 | 0.139 | 0.151 | 0.216 | 0.287 | 0.398 | 0.704 | 0.828 |
| 16 | -0.367 | 0.439 | 0.143 | 0.155 | 0.223 | 0.295 | 0.403 | 0.683 | 0.789 |
| 18 | -0.310 | 0.428 | 0.149 | 0.161 | 0.231 | 0.305 | 0.412 | 0.674 | 0.769 |
| 20 | -0.259 | 0.418 | 0.155 | 0.168 | 0.241 | 0.316 | 0.423 | 0.674 | 0.761 |
| 25 | -0.152 | 0.396 | 0.170 | 0.185 | 0.266 | 0.345 | 0.453 | 0.686 | 0.761 |
| 30 | -0.063 | 0.379 | 0.185 | 0.202 | 0.288 | 0.371 | 0.481 | 0.702 | 0.770 |
| 35 | 0.011 | 0.364 | 0.198 | 0.216 | 0.308 | 0.394 | 0.504 | 0.716 | 0.780 |
| 40 | 0.076 | 0.352 | 0.210 | 0.229 | 0.325 | 0.413 | 0.523 | 0.728 | 0.788 |
| 45 | 0.133 | 0.340 | 0.220 | 0.240 | 0.340 | 0.430 | 0.539 | 0.737 | 0.794 |
| 50 | 0.184 | 0.330 | 0.229 | 0.250 | 0.353 | 0.443 | 0.551 | 0.744 | 0.798 |
| 55 | 0.230 | 0.321 | 0.237 | 0.259 | 0.364 | 0.455 | 0.562 | 0.748 | 0.800 |
| 60 | 0.272 | 0.313 | 0.244 | 0.267 | 0.373 | 0.464 | 0.570 | 0.751 | 0.801 |
| 65 | 0.311 | 0.305 | 0.251 | 0.273 | 0.381 | 0.472 | 0.576 | 0.752 | 0.800 |
| 70 | 0.347 | 0.298 | 0.256 | 0.279 | 0.388 | 0.478 | 0.581 | 0.752 | 0.798 |
| 75 | 0.380 | 0.292 | 0.261 | 0.285 | 0.394 | 0.484 | 0.584 | 0.751 | 0.796 |
| 80 | 0.411 | 0.285 | 0.266 | 0.290 | 0.400 | 0.488 | 0.588 | 0.750 | 0.793 |
| 85 | 0.440 | 0.280 | 0.271 | 0.295 | 0.405 | 0.493 | 0.590 | 0.749 | 0.791 |
