# Supplementary material for: Dual energy X-ray absorptiometry body composition reference values of limbs and trunk from NHANES 1999–2004 with additional visualization methods
Source: PLoS One. 2017 Mar 27;12(3):e0174180. doi: 10.1371/journal.pone.0174180 (PMC5367711; doi:10.1371/journal.pone.0174180)
Supplement: S35 Table — This table provides L, M, and S values to derive average arm LMI Z-scores for 3rd through 97th percentiles for white females ages 8–85. (DOCX) [file pone.0174180.s043.docx]

Table S35: LMS Curve Fit Data providing L, M, and S values for 3^rd^ through 97^th^ percentiles for White Females Ages 8-85 for Average Arm LMI.

|  | Females | | | | | | | | |
| --- | --- | --- | --- | --- | --- | --- | --- | --- | --- |
|  |  |  | M | | | | | | |
|  |  |  | 3 | 5 | 25 | 50 | 75 | 95 | 97 |
| Age | L | S | -1.881 | -1.645 | -0.674 | 0 | 0.674 | 1.645 | 1.881 |
| 8 | -0.383 | 0.157 | 0.498 | 0.515 | 0.594 | 0.659 | 0.734 | 0.865 | 0.902 |
| 10 | -0.383 | 0.157 | 0.532 | 0.550 | 0.634 | 0.704 | 0.784 | 0.924 | 0.963 |
| 12 | -0.383 | 0.157 | 0.558 | 0.577 | 0.665 | 0.738 | 0.823 | 0.970 | 1.011 |
| 14 | -0.383 | 0.157 | 0.578 | 0.597 | 0.689 | 0.764 | 0.852 | 1.004 | 1.046 |
| 16 | -0.383 | 0.157 | 0.592 | 0.613 | 0.707 | 0.784 | 0.874 | 1.029 | 1.073 |
| 18 | -0.383 | 0.157 | 0.603 | 0.624 | 0.720 | 0.799 | 0.890 | 1.049 | 1.093 |
| 20 | -0.383 | 0.157 | 0.612 | 0.632 | 0.730 | 0.809 | 0.902 | 1.063 | 1.108 |
| 25 | -0.383 | 0.157 | 0.624 | 0.645 | 0.744 | 0.826 | 0.920 | 1.084 | 1.130 |
| 30 | -0.383 | 0.157 | 0.629 | 0.651 | 0.751 | 0.833 | 0.928 | 1.094 | 1.140 |
| 35 | -0.383 | 0.157 | 0.631 | 0.652 | 0.752 | 0.835 | 0.930 | 1.096 | 1.142 |
| 40 | -0.383 | 0.157 | 0.629 | 0.651 | 0.751 | 0.833 | 0.928 | 1.094 | 1.140 |
| 45 | -0.383 | 0.157 | 0.626 | 0.647 | 0.747 | 0.829 | 0.923 | 1.088 | 1.134 |
| 50 | -0.383 | 0.157 | 0.621 | 0.642 | 0.741 | 0.822 | 0.916 | 1.079 | 1.125 |
| 55 | -0.383 | 0.157 | 0.615 | 0.636 | 0.733 | 0.814 | 0.907 | 1.068 | 1.114 |
| 60 | -0.383 | 0.157 | 0.608 | 0.628 | 0.725 | 0.804 | 0.896 | 1.056 | 1.101 |
| 65 | -0.383 | 0.157 | 0.600 | 0.620 | 0.716 | 0.794 | 0.885 | 1.043 | 1.087 |
| 70 | -0.383 | 0.157 | 0.592 | 0.612 | 0.706 | 0.784 | 0.873 | 1.029 | 1.073 |
| 75 | -0.383 | 0.157 | 0.585 | 0.605 | 0.697 | 0.774 | 0.862 | 1.016 | 1.059 |
| 80 | -0.383 | 0.157 | 0.577 | 0.597 | 0.688 | 0.764 | 0.851 | 1.003 | 1.046 |
| 85 | -0.383 | 0.157 | 0.570 | 0.590 | 0.680 | 0.755 | 0.841 | 0.991 | 1.033 |
