# Supplementary material for: Dual energy X-ray absorptiometry body composition reference values of limbs and trunk from NHANES 1999–2004 with additional visualization methods
Source: PLoS One. 2017 Mar 27;12(3):e0174180. doi: 10.1371/journal.pone.0174180 (PMC5367711; doi:10.1371/journal.pone.0174180)
Supplement: S36 Table — This table provides L, M, and S values to derive average arm LMI Z-scores for 3rd through 97th percentiles for white males ages 8–85. (DOCX) [file pone.0174180.s044.docx]

Table S36: LMS Curve Fit Data providing L, M, and S values for 3^rd^ through 97^th^ percentiles for White Males Ages 8-85 for Average Arm LMI.

|  | Males | | | | | | | | |
| --- | --- | --- | --- | --- | --- | --- | --- | --- | --- |
|  |  |  | M | | | | | | |
|  |  |  | 3 | 5 | 25 | 50 | 75 | 95 | 97 |
| Age | L | S | -1.881 | -1.645 | -0.674 | 0 | 0.674 | 1.645 | 1.881 |
| 8 | 0.512 | 0.141 | 0.514 | 0.534 | 0.621 | 0.684 | 0.751 | 0.852 | 0.878 |
| 10 | 0.512 | 0.141 | 0.554 | 0.575 | 0.668 | 0.737 | 0.808 | 0.918 | 0.945 |
| 12 | 0.512 | 0.141 | 0.634 | 0.659 | 0.766 | 0.844 | 0.927 | 1.052 | 1.083 |
| 14 | 0.512 | 0.141 | 0.740 | 0.769 | 0.894 | 0.985 | 1.081 | 1.227 | 1.264 |
| 16 | 0.512 | 0.141 | 0.830 | 0.862 | 1.001 | 1.104 | 1.212 | 1.375 | 1.416 |
| 18 | 0.512 | 0.141 | 0.889 | 0.924 | 1.073 | 1.183 | 1.299 | 1.474 | 1.518 |
| 20 | 0.512 | 0.141 | 0.925 | 0.961 | 1.116 | 1.230 | 1.350 | 1.533 | 1.579 |
| 25 | 0.512 | 0.141 | 0.960 | 0.998 | 1.159 | 1.278 | 1.403 | 1.592 | 1.640 |
| 30 | 0.512 | 0.141 | 0.973 | 1.011 | 1.174 | 1.295 | 1.421 | 1.613 | 1.661 |
| 35 | 0.512 | 0.141 | 0.982 | 1.020 | 1.185 | 1.307 | 1.434 | 1.628 | 1.677 |
| 40 | 0.512 | 0.141 | 0.985 | 1.024 | 1.189 | 1.311 | 1.439 | 1.633 | 1.682 |
| 45 | 0.512 | 0.141 | 0.981 | 1.019 | 1.184 | 1.306 | 1.433 | 1.626 | 1.675 |
| 50 | 0.512 | 0.141 | 0.970 | 1.008 | 1.170 | 1.291 | 1.416 | 1.608 | 1.656 |
| 55 | 0.512 | 0.141 | 0.953 | 0.991 | 1.151 | 1.269 | 1.392 | 1.580 | 1.628 |
| 60 | 0.512 | 0.141 | 0.933 | 0.969 | 1.126 | 1.242 | 1.363 | 1.547 | 1.593 |
| 65 | 0.512 | 0.141 | 0.908 | 0.943 | 1.096 | 1.208 | 1.326 | 1.505 | 1.550 |
| 70 | 0.512 | 0.141 | 0.879 | 0.913 | 1.061 | 1.170 | 1.284 | 1.457 | 1.501 |
| 75 | 0.512 | 0.141 | 0.847 | 0.880 | 1.023 | 1.127 | 1.237 | 1.404 | 1.447 |
| 80 | 0.512 | 0.141 | 0.815 | 0.847 | 0.983 | 1.084 | 1.190 | 1.351 | 1.391 |
| 85 | 0.512 | 0.141 | 0.783 | 0.814 | 0.946 | 1.043 | 1.144 | 1.299 | 1.338 |
