# Supplementary material for: Dual energy X-ray absorptiometry body composition reference values of limbs and trunk from NHANES 1999–2004 with additional visualization methods
Source: PLoS One. 2017 Mar 27;12(3):e0174180. doi: 10.1371/journal.pone.0174180 (PMC5367711; doi:10.1371/journal.pone.0174180)
Supplement: S37 Table — This table provides L, M, and S values to derive average leg FMI Z-scores for 3rd through 97th percentiles for white females ages 8–85. (DOCX) [file pone.0174180.s045.docx]

Table S37: LMS Curve Fit Data providing L, M, and S values for 3^rd^ through 97^th^ percentiles for White Females Ages 8-85 for Average Leg FMI.

|  | Females | | | | | | | | |
| --- | --- | --- | --- | --- | --- | --- | --- | --- | --- |
|  |  |  | M | | | | | | |
|  |  |  | 3 | 5 | 25 | 50 | 75 | 95 | 97 |
| Age | L | S | -1.881 | -1.645 | -0.674 | 0 | 0.674 | 1.645 | 1.881 |
| 8 | -0.458 | 0.331 | 0.707 | 0.752 | 0.988 | 1.222 | 1.546 | 2.283 | 2.540 |
| 10 | -0.386 | 0.331 | 0.769 | 0.820 | 1.085 | 1.343 | 1.695 | 2.472 | 2.734 |
| 12 | -0.328 | 0.331 | 0.818 | 0.874 | 1.162 | 1.441 | 1.816 | 2.623 | 2.888 |
| 14 | -0.279 | 0.331 | 0.858 | 0.918 | 1.227 | 1.523 | 1.916 | 2.745 | 3.013 |
| 16 | -0.236 | 0.331 | 0.891 | 0.954 | 1.280 | 1.591 | 2.000 | 2.846 | 3.116 |
| 18 | -0.198 | 0.331 | 0.917 | 0.983 | 1.325 | 1.648 | 2.069 | 2.929 | 3.199 |
| 20 | -0.164 | 0.331 | 0.938 | 1.007 | 1.362 | 1.695 | 2.127 | 2.996 | 3.266 |
| 25 | -0.093 | 0.331 | 0.974 | 1.049 | 1.430 | 1.782 | 2.232 | 3.114 | 3.381 |
| 30 | -0.035 | 0.331 | 0.995 | 1.075 | 1.475 | 1.841 | 2.303 | 3.188 | 3.452 |
| 35 | 0.015 | 0.331 | 1.009 | 1.091 | 1.507 | 1.884 | 2.353 | 3.237 | 3.498 |
| 40 | 0.057 | 0.331 | 1.017 | 1.102 | 1.531 | 1.915 | 2.390 | 3.271 | 3.528 |
| 45 | 0.095 | 0.331 | 1.022 | 1.110 | 1.548 | 1.939 | 2.418 | 3.295 | 3.548 |
| 50 | 0.129 | 0.331 | 1.024 | 1.114 | 1.562 | 1.958 | 2.439 | 3.312 | 3.561 |
| 55 | 0.159 | 0.331 | 1.025 | 1.117 | 1.573 | 1.973 | 2.456 | 3.323 | 3.569 |
| 60 | 0.187 | 0.331 | 1.025 | 1.119 | 1.581 | 1.985 | 2.469 | 3.331 | 3.574 |
| 65 | 0.213 | 0.331 | 1.024 | 1.119 | 1.587 | 1.994 | 2.479 | 3.336 | 3.575 |
| 70 | 0.237 | 0.331 | 1.022 | 1.119 | 1.592 | 2.002 | 2.487 | 3.338 | 3.575 |
| 75 | 0.259 | 0.331 | 1.020 | 1.118 | 1.597 | 2.008 | 2.494 | 3.340 | 3.574 |
| 80 | 0.279 | 0.331 | 1.017 | 1.117 | 1.600 | 2.014 | 2.500 | 3.341 | 3.573 |
| 85 | 0.299 | 0.331 | 1.015 | 1.116 | 1.603 | 2.019 | 2.505 | 3.342 | 3.571 |
