# Supplementary material for: Dual energy X-ray absorptiometry body composition reference values of limbs and trunk from NHANES 1999–2004 with additional visualization methods
Source: PLoS One. 2017 Mar 27;12(3):e0174180. doi: 10.1371/journal.pone.0174180 (PMC5367711; doi:10.1371/journal.pone.0174180)
Supplement: S38 Table — This table provides L, M, and S values to derive average leg FMI Z-scores for 3rd through 97th percentiles for white males ages 8–85. (DOCX) [file pone.0174180.s046.docx]

Table S38: LMS Curve Fit Data providing L, M, and S values for 3^rd^ through 97^th^ percentiles for White Females Ages 8-85 for Average Leg FMI.

|  | Males | | | | | | | | |
| --- | --- | --- | --- | --- | --- | --- | --- | --- | --- |
|  |  |  | M | | | | | | |
|  |  |  | 3 | 5 | 25 | 50 | 75 | 95 | 97 |
| Age | L | S | -1.881 | -1.645 | -0.674 | 0 | 0.674 | 1.645 | 1.881 |
| 8 | -0.027 | 0.485 | 0.419 | 0.468 | 0.745 | 1.032 | 1.433 | 2.311 | 2.598 |
| 10 | -0.027 | 0.466 | 0.439 | 0.489 | 0.764 | 1.045 | 1.433 | 2.270 | 2.540 |
| 12 | -0.027 | 0.451 | 0.457 | 0.507 | 0.781 | 1.057 | 1.434 | 2.235 | 2.492 |
| 14 | -0.027 | 0.437 | 0.473 | 0.523 | 0.795 | 1.067 | 1.434 | 2.206 | 2.451 |
| 16 | -0.027 | 0.426 | 0.487 | 0.537 | 0.808 | 1.075 | 1.434 | 2.180 | 2.416 |
| 18 | -0.027 | 0.415 | 0.500 | 0.551 | 0.820 | 1.083 | 1.434 | 2.158 | 2.384 |
| 20 | -0.027 | 0.406 | 0.512 | 0.563 | 0.830 | 1.090 | 1.434 | 2.137 | 2.356 |
| 25 | -0.027 | 0.385 | 0.539 | 0.590 | 0.853 | 1.105 | 1.434 | 2.094 | 2.297 |
| 30 | -0.027 | 0.368 | 0.563 | 0.613 | 0.873 | 1.118 | 1.434 | 2.058 | 2.248 |
| 35 | -0.027 | 0.353 | 0.584 | 0.634 | 0.890 | 1.129 | 1.433 | 2.027 | 2.207 |
| 40 | -0.027 | 0.340 | 0.603 | 0.653 | 0.905 | 1.138 | 1.433 | 2.001 | 2.171 |
| 45 | -0.027 | 0.329 | 0.621 | 0.670 | 0.919 | 1.147 | 1.432 | 1.977 | 2.140 |
| 50 | -0.027 | 0.318 | 0.637 | 0.686 | 0.932 | 1.154 | 1.432 | 1.956 | 2.111 |
| 55 | -0.027 | 0.309 | 0.652 | 0.701 | 0.944 | 1.161 | 1.431 | 1.937 | 2.086 |
| 60 | -0.027 | 0.300 | 0.667 | 0.715 | 0.954 | 1.168 | 1.430 | 1.920 | 2.063 |
| 65 | -0.027 | 0.292 | 0.680 | 0.728 | 0.965 | 1.174 | 1.430 | 1.903 | 2.041 |
| 70 | -0.027 | 0.284 | 0.693 | 0.741 | 0.974 | 1.179 | 1.429 | 1.888 | 2.021 |
| 75 | -0.027 | 0.277 | 0.706 | 0.753 | 0.983 | 1.185 | 1.429 | 1.874 | 2.003 |
| 80 | -0.027 | 0.271 | 0.718 | 0.764 | 0.992 | 1.190 | 1.428 | 1.861 | 1.986 |
| 85 | -0.027 | 0.264 | 0.729 | 0.775 | 1.000 | 1.194 | 1.428 | 1.849 | 1.969 |
