# Supplementary material for: Dual energy X-ray absorptiometry body composition reference values of limbs and trunk from NHANES 1999–2004 with additional visualization methods
Source: PLoS One. 2017 Mar 27;12(3):e0174180. doi: 10.1371/journal.pone.0174180 (PMC5367711; doi:10.1371/journal.pone.0174180)
Supplement: S39 Table — This table provides L, M, and S values to derive average leg LMI Z-scores for 3rd through 97th percentiles for white males ages 8–85. (DOCX) [file pone.0174180.s047.docx]

Table S39: LMS Curve Fit Data providing L, M, and S values for 3^rd^ through 97^th^ percentiles for White Females Ages 8-85 for Average Leg LMI.

|  | Females | | | | | | | | |
| --- | --- | --- | --- | --- | --- | --- | --- | --- | --- |
|  |  |  | M | | | | | | |
|  |  |  | 3 | 5 | 25 | 50 | 75 | 95 | 97 |
| Age | L | S | -1.881 | -1.645 | -0.674 | 0 | 0.674 | 1.645 | 1.881 |
| 8 | -0.402 | 0.153 | 1.485 | 1.534 | 1.762 | 1.950 | 2.166 | 2.542 | 2.647 |
| 10 | -0.402 | 0.153 | 1.661 | 1.716 | 1.971 | 2.181 | 2.423 | 2.843 | 2.961 |
| 12 | -0.402 | 0.153 | 1.785 | 1.844 | 2.118 | 2.344 | 2.604 | 3.056 | 3.182 |
| 14 | -0.402 | 0.153 | 1.866 | 1.927 | 2.214 | 2.450 | 2.722 | 3.194 | 3.326 |
| 16 | -0.402 | 0.153 | 1.915 | 1.978 | 2.273 | 2.514 | 2.794 | 3.278 | 3.414 |
| 18 | -0.402 | 0.153 | 1.946 | 2.011 | 2.310 | 2.555 | 2.839 | 3.332 | 3.470 |
| 20 | -0.402 | 0.153 | 1.966 | 2.031 | 2.334 | 2.582 | 2.868 | 3.366 | 3.505 |
| 25 | -0.402 | 0.153 | 1.983 | 2.049 | 2.354 | 2.604 | 2.893 | 3.395 | 3.536 |
| 30 | -0.402 | 0.153 | 1.981 | 2.047 | 2.352 | 2.602 | 2.891 | 3.392 | 3.532 |
| 35 | -0.402 | 0.153 | 1.975 | 2.041 | 2.345 | 2.594 | 2.882 | 3.382 | 3.522 |
| 40 | -0.402 | 0.153 | 1.969 | 2.034 | 2.337 | 2.586 | 2.873 | 3.371 | 3.510 |
| 45 | -0.402 | 0.153 | 1.959 | 2.024 | 2.325 | 2.572 | 2.858 | 3.353 | 3.492 |
| 50 | -0.402 | 0.153 | 1.942 | 2.007 | 2.305 | 2.550 | 2.834 | 3.325 | 3.463 |
| 55 | -0.402 | 0.153 | 1.921 | 1.985 | 2.280 | 2.522 | 2.802 | 3.289 | 3.424 |
| 60 | -0.402 | 0.153 | 1.897 | 1.959 | 2.251 | 2.490 | 2.767 | 3.247 | 3.381 |
| 65 | -0.402 | 0.153 | 1.870 | 1.932 | 2.220 | 2.456 | 2.729 | 3.202 | 3.334 |
| 70 | -0.402 | 0.153 | 1.843 | 1.904 | 2.187 | 2.420 | 2.688 | 3.155 | 3.285 |
| 75 | -0.402 | 0.153 | 1.815 | 1.875 | 2.154 | 2.383 | 2.647 | 3.107 | 3.235 |
| 80 | -0.402 | 0.153 | 1.787 | 1.846 | 2.120 | 2.346 | 2.606 | 3.059 | 3.185 |
| 85 | -0.402 | 0.153 | 1.759 | 1.818 | 2.088 | 2.310 | 2.567 | 3.012 | 3.137 |
