# Supplementary material for: Dual energy X-ray absorptiometry body composition reference values of limbs and trunk from NHANES 1999–2004 with additional visualization methods
Source: PLoS One. 2017 Mar 27;12(3):e0174180. doi: 10.1371/journal.pone.0174180 (PMC5367711; doi:10.1371/journal.pone.0174180)
Supplement: S40 Table — This table provides L, M, and S values to derive average leg LMI Z-scores for 3rd through 97th percentiles for white males ages 8–85. (DOCX) [file pone.0174180.s048.docx]

Table S40: LMS Curve Fit Data providing L, M, and S values for 3^rd^ through 97^th^ percentiles for White Males Ages 8-85 for Average Leg LMI.

|  | Males | | | | | | | | |
| --- | --- | --- | --- | --- | --- | --- | --- | --- | --- |
|  |  |  | M | | | | | | |
|  |  |  | 3 | 5 | 25 | 50 | 75 | 95 | 97 |
| Age | L | S | -1.881 | -1.645 | -0.674 | 0 | 0.674 | 1.645 | 1.881 |
| 8 | -0.392 | 0.146 | 1.525 | 1.573 | 1.797 | 1.980 | 2.189 | 2.549 | 2.649 |
| 10 | -0.243 | 0.143 | 1.731 | 1.788 | 2.043 | 2.248 | 2.479 | 2.866 | 2.971 |
| 12 | -0.122 | 0.141 | 1.953 | 2.017 | 2.307 | 2.535 | 2.789 | 3.207 | 3.319 |
| 14 | -0.020 | 0.139 | 2.160 | 2.232 | 2.553 | 2.803 | 3.079 | 3.525 | 3.643 |
| 16 | 0.069 | 0.137 | 2.309 | 2.386 | 2.730 | 2.995 | 3.284 | 3.746 | 3.867 |
| 18 | 0.148 | 0.136 | 2.395 | 2.476 | 2.833 | 3.106 | 3.401 | 3.868 | 3.989 |
| 20 | 0.218 | 0.134 | 2.440 | 2.523 | 2.887 | 3.163 | 3.460 | 3.924 | 4.044 |
| 25 | 0.366 | 0.131 | 2.470 | 2.555 | 2.924 | 3.199 | 3.490 | 3.938 | 4.052 |
| 30 | 0.488 | 0.129 | 2.470 | 2.556 | 2.926 | 3.197 | 3.481 | 3.911 | 4.020 |
| 35 | 0.590 | 0.127 | 2.474 | 2.561 | 2.931 | 3.199 | 3.477 | 3.894 | 3.999 |
| 40 | 0.679 | 0.125 | 2.475 | 2.563 | 2.932 | 3.198 | 3.471 | 3.877 | 3.978 |
| 45 | 0.758 | 0.123 | 2.467 | 2.555 | 2.923 | 3.185 | 3.453 | 3.847 | 3.945 |
| 50 | 0.828 | 0.122 | 2.448 | 2.536 | 2.901 | 3.159 | 3.420 | 3.803 | 3.897 |
| 55 | 0.891 | 0.121 | 2.422 | 2.509 | 2.869 | 3.122 | 3.377 | 3.749 | 3.840 |
| 60 | 0.949 | 0.120 | 2.389 | 2.475 | 2.830 | 3.078 | 3.326 | 3.686 | 3.774 |
| 65 | 1.002 | 0.119 | 2.349 | 2.434 | 2.782 | 3.024 | 3.266 | 3.614 | 3.699 |
| 70 | 1.052 | 0.118 | 2.303 | 2.386 | 2.727 | 2.962 | 3.197 | 3.533 | 3.614 |
| 75 | 1.098 | 0.117 | 2.254 | 2.336 | 2.669 | 2.897 | 3.125 | 3.449 | 3.527 |
| 80 | 1.140 | 0.116 | 2.205 | 2.285 | 2.610 | 2.833 | 3.053 | 3.366 | 3.441 |
| 85 | 1.181 | 0.115 | 2.158 | 2.236 | 2.554 | 2.770 | 2.984 | 3.286 | 3.359 |
