# Supplementary material for: Dual energy X-ray absorptiometry body composition reference values of limbs and trunk from NHANES 1999–2004 with additional visualization methods
Source: PLoS One. 2017 Mar 27;12(3):e0174180. doi: 10.1371/journal.pone.0174180 (PMC5367711; doi:10.1371/journal.pone.0174180)
Supplement: S41 Table — This table provides L, M, and S values to derive trunk FMI Z-scores for 3rd through 97th percentiles for white females ages 8–85. (DOCX) [file pone.0174180.s049.docx]

Table S41: LMS Curve Fit Data providing L, M, and S values for 3^rd^ through 97^th^ percentiles for White Females Ages 8-85 for Trunk FMI.

|  | Females | | | | | | | | |
| --- | --- | --- | --- | --- | --- | --- | --- | --- | --- |
|  |  |  | M | | | | | | |
|  |  |  | 3 | 5 | 25 | 50 | 75 | 95 | 97 |
| Age | L | S | -1.881 | -1.645 | -0.674 | 0 | 0.674 | 1.645 | 1.881 |
| 8 | -0.418 | 0.537 | 0.913 | 1.000 | 1.513 | 2.120 | 3.139 | 6.388 | 7.880 |
| 10 | -0.377 | 0.531 | 0.998 | 1.094 | 1.664 | 2.328 | 3.418 | 6.712 | 8.146 |
| 12 | -0.337 | 0.524 | 1.080 | 1.187 | 1.812 | 2.530 | 3.687 | 7.014 | 8.397 |
| 14 | -0.298 | 0.518 | 1.159 | 1.276 | 1.957 | 2.728 | 3.945 | 7.295 | 8.631 |
| 16 | -0.259 | 0.512 | 1.235 | 1.362 | 2.098 | 2.920 | 4.192 | 7.554 | 8.846 |
| 18 | -0.221 | 0.506 | 1.308 | 1.445 | 2.233 | 3.103 | 4.424 | 7.788 | 9.038 |
| 20 | -0.183 | 0.500 | 1.376 | 1.523 | 2.363 | 3.278 | 4.642 | 7.997 | 9.206 |
| 25 | -0.090 | 0.486 | 1.527 | 1.698 | 2.660 | 3.673 | 5.121 | 8.416 | 9.529 |
| 30 | 0.001 | 0.471 | 1.656 | 1.851 | 2.926 | 4.021 | 5.524 | 8.728 | 9.753 |
| 35 | 0.091 | 0.457 | 1.768 | 1.987 | 3.168 | 4.331 | 5.870 | 8.966 | 9.915 |
| 40 | 0.179 | 0.443 | 1.870 | 2.112 | 3.394 | 4.615 | 6.174 | 9.160 | 10.040 |
| 45 | 0.267 | 0.430 | 1.961 | 2.227 | 3.606 | 4.875 | 6.443 | 9.316 | 10.136 |
| 50 | 0.353 | 0.416 | 2.041 | 2.330 | 3.797 | 5.103 | 6.667 | 9.421 | 10.186 |
| 55 | 0.439 | 0.403 | 2.104 | 2.415 | 3.958 | 5.284 | 6.830 | 9.454 | 10.165 |
| 60 | 0.523 | 0.390 | 2.146 | 2.477 | 4.076 | 5.406 | 6.914 | 9.395 | 10.052 |
| 65 | 0.607 | 0.376 | 2.164 | 2.512 | 4.145 | 5.460 | 6.913 | 9.235 | 9.839 |
| 70 | 0.691 | 0.363 | 2.160 | 2.520 | 4.164 | 5.446 | 6.828 | 8.981 | 9.531 |
| 75 | 0.773 | 0.350 | 2.137 | 2.504 | 4.138 | 5.371 | 6.672 | 8.650 | 9.148 |
| 80 | 0.856 | 0.337 | 2.104 | 2.475 | 4.080 | 5.255 | 6.468 | 8.275 | 8.724 |
| 85 | 0.937 | 0.325 | 2.069 | 2.441 | 4.008 | 5.120 | 6.248 | 7.895 | 8.298 |
