# Supplementary material for: Dual energy X-ray absorptiometry body composition reference values of limbs and trunk from NHANES 1999–2004 with additional visualization methods
Source: PLoS One. 2017 Mar 27;12(3):e0174180. doi: 10.1371/journal.pone.0174180 (PMC5367711; doi:10.1371/journal.pone.0174180)
Supplement: S42 Table — This table provides L, M, and S values to derive trunk FMI Z-scores for 3rd through 97th percentiles for white males ages 8–85. (DOCX) [file pone.0174180.s050.docx]

Table S42: LMS Curve Fit Data providing L, M, and S values for 3^rd^ through 97^th^ percentiles for White Males Ages 8-85 for Trunk FMI.

|  | Males | | | | | | | | |
| --- | --- | --- | --- | --- | --- | --- | --- | --- | --- |
|  |  |  | M | | | | | | |
|  |  |  | 3 | 5 | 25 | 50 | 75 | 95 | 97 |
| Age | L | S | -1.881 | -1.645 | -0.674 | 0 | 0.674 | 1.645 | 1.881 |
| 8 | -0.749 | 0.524 | 0.735 | 0.791 | 1.124 | 1.537 | 2.316 | 6.137 | 9.193 |
| 10 | -0.663 | 0.513 | 0.778 | 0.839 | 1.202 | 1.640 | 2.429 | 5.649 | 7.653 |
| 12 | -0.584 | 0.503 | 0.821 | 0.887 | 1.279 | 1.743 | 2.542 | 5.395 | 6.904 |
| 14 | -0.511 | 0.493 | 0.868 | 0.941 | 1.364 | 1.855 | 2.670 | 5.289 | 6.523 |
| 16 | -0.441 | 0.484 | 0.924 | 1.004 | 1.464 | 1.987 | 2.826 | 5.302 | 6.370 |
| 18 | -0.375 | 0.476 | 0.988 | 1.076 | 1.579 | 2.138 | 3.009 | 5.400 | 6.362 |
| 20 | -0.311 | 0.468 | 1.058 | 1.155 | 1.704 | 2.302 | 3.208 | 5.548 | 6.436 |
| 25 | -0.163 | 0.449 | 1.233 | 1.354 | 2.024 | 2.719 | 3.708 | 5.971 | 6.743 |
| 30 | -0.027 | 0.431 | 1.389 | 1.535 | 2.320 | 3.099 | 4.149 | 6.342 | 7.037 |
| 35 | 0.101 | 0.415 | 1.521 | 1.691 | 2.582 | 3.429 | 4.518 | 6.633 | 7.266 |
| 40 | 0.222 | 0.399 | 1.634 | 1.827 | 2.816 | 3.716 | 4.827 | 6.861 | 7.444 |
| 45 | 0.337 | 0.385 | 1.731 | 1.947 | 3.024 | 3.966 | 5.085 | 7.037 | 7.577 |
| 50 | 0.447 | 0.370 | 1.814 | 2.052 | 3.207 | 4.179 | 5.296 | 7.165 | 7.667 |
| 55 | 0.552 | 0.357 | 1.883 | 2.143 | 3.366 | 4.357 | 5.461 | 7.246 | 7.713 |
| 60 | 0.654 | 0.344 | 1.937 | 2.215 | 3.492 | 4.490 | 5.571 | 7.266 | 7.700 |
| 65 | 0.752 | 0.331 | 1.970 | 2.265 | 3.577 | 4.567 | 5.614 | 7.210 | 7.613 |
| 70 | 0.848 | 0.319 | 1.984 | 2.291 | 3.618 | 4.588 | 5.590 | 7.082 | 7.453 |
| 75 | 0.940 | 0.307 | 1.985 | 2.301 | 3.627 | 4.566 | 5.517 | 6.904 | 7.244 |
| 80 | 1.031 | 0.296 | 1.980 | 2.303 | 3.617 | 4.521 | 5.418 | 6.704 | 7.015 |
| 85 | 1.119 | 0.284 | 1.978 | 2.305 | 3.601 | 4.468 | 5.315 | 6.508 | 6.793 |
