# Supplementary material for: Dual energy X-ray absorptiometry body composition reference values of limbs and trunk from NHANES 1999–2004 with additional visualization methods
Source: PLoS One. 2017 Mar 27;12(3):e0174180. doi: 10.1371/journal.pone.0174180 (PMC5367711; doi:10.1371/journal.pone.0174180)
Supplement: S43 Table — This table provides L, M, and S values to derive trunk LMI Z-scores for 3rd through 97th percentiles for white females ages 8–85. (DOCX) [file pone.0174180.s051.docx]

Table S43: LMS Curve Fit Data providing L, M, and S values for 3^rd^ through 97^th^ percentiles for White Females Ages 8-85 for Trunk LMI.

|  | Females | | | | | | | | |
| --- | --- | --- | --- | --- | --- | --- | --- | --- | --- |
|  |  |  | M | | | | | | |
|  |  |  | 3 | 5 | 25 | 50 | 75 | 95 | 97 |
| Age | L | S | -1.881 | -1.645 | -0.674 | 0 | 0.674 | 1.645 | 1.881 |
| 8 | -0.737 | 0.127 | 4.557 | 4.676 | 5.226 | 5.678 | 6.203 | 7.124 | 7.384 |
| 10 | -0.737 | 0.127 | 5.003 | 5.133 | 5.738 | 6.234 | 6.810 | 7.821 | 8.107 |
| 12 | -0.737 | 0.127 | 5.353 | 5.493 | 6.139 | 6.670 | 7.287 | 8.368 | 8.674 |
| 14 | -0.737 | 0.127 | 5.626 | 5.773 | 6.452 | 7.010 | 7.658 | 8.795 | 9.116 |
| 16 | -0.737 | 0.127 | 5.835 | 5.987 | 6.691 | 7.270 | 7.942 | 9.121 | 9.454 |
| 18 | -0.737 | 0.127 | 5.993 | 6.149 | 6.873 | 7.467 | 8.157 | 9.368 | 9.710 |
| 20 | -0.737 | 0.127 | 6.113 | 6.272 | 7.010 | 7.616 | 8.320 | 9.555 | 9.904 |
| 25 | -0.737 | 0.127 | 6.300 | 6.464 | 7.225 | 7.850 | 8.575 | 9.848 | 10.208 |
| 30 | -0.737 | 0.127 | 6.398 | 6.565 | 7.337 | 7.972 | 8.709 | 10.001 | 10.367 |
| 35 | -0.737 | 0.127 | 6.450 | 6.618 | 7.397 | 8.037 | 8.779 | 10.083 | 10.451 |
| 40 | -0.737 | 0.127 | 6.475 | 6.644 | 7.425 | 8.068 | 8.813 | 10.122 | 10.492 |
| 45 | -0.737 | 0.127 | 6.481 | 6.650 | 7.432 | 8.075 | 8.822 | 10.131 | 10.501 |
| 50 | -0.737 | 0.127 | 6.472 | 6.641 | 7.423 | 8.065 | 8.810 | 10.118 | 10.487 |
| 55 | -0.737 | 0.127 | 6.453 | 6.621 | 7.401 | 8.041 | 8.784 | 10.088 | 10.457 |
| 60 | -0.737 | 0.127 | 6.428 | 6.595 | 7.371 | 8.009 | 8.749 | 10.048 | 10.415 |
| 65 | -0.737 | 0.127 | 6.399 | 6.565 | 7.338 | 7.973 | 8.709 | 10.002 | 10.368 |
| 70 | -0.737 | 0.127 | 6.368 | 6.533 | 7.302 | 7.934 | 8.667 | 9.954 | 10.318 |
| 75 | -0.737 | 0.127 | 6.336 | 6.501 | 7.266 | 7.895 | 8.624 | 9.905 | 10.266 |
| 80 | -0.737 | 0.127 | 6.305 | 6.469 | 7.231 | 7.856 | 8.582 | 9.856 | 10.216 |
| 85 | -0.737 | 0.127 | 6.275 | 6.439 | 7.196 | 7.819 | 8.542 | 9.809 | 10.168 |
