# Supplementary material for: Dual energy X-ray absorptiometry body composition reference values of limbs and trunk from NHANES 1999–2004 with additional visualization methods
Source: PLoS One. 2017 Mar 27;12(3):e0174180. doi: 10.1371/journal.pone.0174180 (PMC5367711; doi:10.1371/journal.pone.0174180)
Supplement: S44 Table — This table provides L, M, and S values to derive trunk LMI Z-scores for 3rd through 97th percentiles for white males ages 8–85. (DOCX) [file pone.0174180.s052.docx]

Table S44: LMS Curve Fit Data providing L, M, and S values for 3^rd^ through 97^th^ percentiles for White Males Ages 8-85 for Trunk LMI.

|  | Males | | | | | | | | |
| --- | --- | --- | --- | --- | --- | --- | --- | --- | --- |
|  |  |  | M | | | | | | |
|  |  |  | 3 | 5 | 25 | 50 | 75 | 95 | 97 |
| Age | L | S | -1.881 | -1.645 | -0.674 | 0 | 0.674 | 1.645 | 1.881 |
| 8 | -0.652 | 0.135 | 4.508 | 4.634 | 5.222 | 5.705 | 6.268 | 7.257 | 7.536 |
| 10 | -0.494 | 0.133 | 4.857 | 4.996 | 5.631 | 6.146 | 6.734 | 7.742 | 8.021 |
| 12 | -0.366 | 0.130 | 5.351 | 5.505 | 6.208 | 6.769 | 7.402 | 8.464 | 8.754 |
| 14 | -0.257 | 0.129 | 5.958 | 6.132 | 6.915 | 7.534 | 8.225 | 9.365 | 9.672 |
| 16 | -0.163 | 0.127 | 6.499 | 6.690 | 7.546 | 8.215 | 8.955 | 10.161 | 10.482 |
| 18 | -0.080 | 0.126 | 6.878 | 7.081 | 7.988 | 8.691 | 9.462 | 10.704 | 11.032 |
| 20 | -0.006 | 0.124 | 7.111 | 7.323 | 8.262 | 8.984 | 9.769 | 11.024 | 11.352 |
| 25 | 0.152 | 0.122 | 7.348 | 7.570 | 8.541 | 9.276 | 10.063 | 11.297 | 11.615 |
| 30 | 0.280 | 0.119 | 7.442 | 7.668 | 8.652 | 9.386 | 10.164 | 11.365 | 11.671 |
| 35 | 0.389 | 0.118 | 7.531 | 7.761 | 8.756 | 9.491 | 10.261 | 11.437 | 11.734 |
| 40 | 0.483 | 0.116 | 7.618 | 7.853 | 8.859 | 9.594 | 10.359 | 11.515 | 11.806 |
| 45 | 0.566 | 0.115 | 7.688 | 7.926 | 8.940 | 9.675 | 10.435 | 11.573 | 11.857 |
| 50 | 0.641 | 0.113 | 7.727 | 7.968 | 8.986 | 9.718 | 10.470 | 11.589 | 11.867 |
| 55 | 0.708 | 0.112 | 7.738 | 7.980 | 8.998 | 9.725 | 10.469 | 11.567 | 11.839 |
| 60 | 0.769 | 0.111 | 7.720 | 7.962 | 8.976 | 9.696 | 10.428 | 11.504 | 11.770 |
| 65 | 0.826 | 0.110 | 7.665 | 7.906 | 8.911 | 9.621 | 10.340 | 11.392 | 11.650 |
| 70 | 0.878 | 0.109 | 7.577 | 7.816 | 8.808 | 9.505 | 10.209 | 11.232 | 11.483 |
| 75 | 0.927 | 0.108 | 7.467 | 7.703 | 8.679 | 9.362 | 10.048 | 11.042 | 11.285 |
| 80 | 0.972 | 0.108 | 7.347 | 7.580 | 8.539 | 9.207 | 9.875 | 10.841 | 11.076 |
| 85 | 1.015 | 0.107 | 7.230 | 7.460 | 8.402 | 9.055 | 9.707 | 10.646 | 10.874 |
