# Supplementary material for: Dual energy X-ray absorptiometry body composition reference values of limbs and trunk from NHANES 1999–2004 with additional visualization methods
Source: PLoS One. 2017 Mar 27;12(3):e0174180. doi: 10.1371/journal.pone.0174180 (PMC5367711; doi:10.1371/journal.pone.0174180)
Supplement: S45 Table — This table provides L, M, and S values to derive total body FMI Z-scores for 3rd through 97th percentiles for white females ages 8–85. (DOCX) [file pone.0174180.s053.docx]

Table S45: LMS Curve Fit Data providing L, M, and S values for 3^rd^ through 97^th^ percentiles for White Females Ages 8-85 for Total Body FMI.

|  | Females | | | | | | | | |
| --- | --- | --- | --- | --- | --- | --- | --- | --- | --- |
|  |  |  | M | | | | | | |
| Age | L | S | 3 | 5 | 25 | 50 | 75 | 95 | 97 |
| 8 | -0.471 | 0.422 | 3.048 | 3.281 | 4.581 | 5.981 | 8.116 | 13.856 | 16.150 |
| 10 | -0.432 | 0.417 | 3.227 | 3.478 | 4.865 | 6.345 | 8.564 | 14.327 | 16.554 |
| 12 | -0.394 | 0.413 | 3.402 | 3.670 | 5.145 | 6.700 | 8.998 | 14.776 | 16.941 |
| 14 | -0.357 | 0.409 | 3.572 | 3.856 | 5.418 | 7.046 | 9.417 | 15.202 | 17.311 |
| 16 | -0.320 | 0.405 | 3.735 | 4.037 | 5.682 | 7.380 | 9.817 | 15.600 | 17.656 |
| 18 | -0.284 | 0.400 | 3.890 | 4.208 | 5.935 | 7.698 | 10.194 | 15.963 | 17.966 |
| 20 | -0.248 | 0.396 | 4.035 | 4.370 | 6.174 | 7.997 | 10.543 | 16.285 | 18.236 |
| 25 | -0.160 | 0.386 | 4.353 | 4.726 | 6.707 | 8.656 | 11.294 | 16.918 | 18.740 |
| 30 | -0.073 | 0.376 | 4.619 | 5.027 | 7.164 | 9.212 | 11.900 | 17.360 | 19.059 |
| 35 | 0.012 | 0.367 | 4.848 | 5.289 | 7.566 | 9.691 | 12.404 | 17.678 | 19.263 |
| 40 | 0.096 | 0.357 | 5.054 | 5.528 | 7.933 | 10.121 | 12.840 | 17.925 | 19.408 |
| 45 | 0.179 | 0.348 | 5.243 | 5.750 | 8.274 | 10.513 | 13.226 | 18.124 | 19.515 |
| 50 | 0.261 | 0.338 | 5.413 | 5.950 | 8.582 | 10.857 | 13.550 | 18.259 | 19.565 |
| 55 | 0.342 | 0.329 | 5.552 | 6.117 | 8.838 | 11.132 | 13.786 | 18.294 | 19.520 |
| 60 | 0.422 | 0.320 | 5.648 | 6.237 | 9.023 | 11.312 | 13.906 | 18.196 | 19.341 |
| 65 | 0.502 | 0.311 | 5.695 | 6.302 | 9.120 | 11.381 | 13.890 | 17.943 | 19.006 |
| 70 | 0.581 | 0.302 | 5.690 | 6.308 | 9.127 | 11.334 | 13.738 | 17.534 | 18.515 |
| 75 | 0.660 | 0.293 | 5.638 | 6.262 | 9.051 | 11.184 | 13.464 | 16.995 | 17.894 |
| 80 | 0.738 | 0.284 | 5.556 | 6.179 | 8.916 | 10.960 | 13.109 | 16.374 | 17.196 |
| 85 | 0.815 | 0.275 | 5.463 | 6.083 | 8.753 | 10.704 | 12.722 | 15.736 | 16.486 |
|  |  |  |  |  |  |  |  |  |  |
