# Supplementary material for: Dual energy X-ray absorptiometry body composition reference values of limbs and trunk from NHANES 1999–2004 with additional visualization methods
Source: PLoS One. 2017 Mar 27;12(3):e0174180. doi: 10.1371/journal.pone.0174180 (PMC5367711; doi:10.1371/journal.pone.0174180)
Supplement: S46 Table — This table provides L, M, and S values to derive total body FMI Z-scores for 3rd through 97th percentiles for white males ages 8–85. (DOCX) [file pone.0174180.s054.docx]

Table S46: LMS Curve Fit Data providing L, M, and S values for 3^rd^ through 97^th^ percentiles for White Males Ages 8-85 for Total Body FMI.

|  | Males | | | | | | | | |
| --- | --- | --- | --- | --- | --- | --- | --- | --- | --- |
|  |  |  | M | | | | | | |
| Age | L | S | 3 | 5 | 25 | 50 | 75 | 95 | 97 |
| 8 | -0.683 | 0.452 | 2.340 | 2.507 | 3.469 | 4.576 | 6.440 | 12.928 | 16.347 |
| 10 | -0.602 | 0.442 | 2.411 | 2.589 | 3.597 | 4.729 | 6.566 | 12.299 | 14.970 |
| 12 | -0.530 | 0.432 | 2.478 | 2.665 | 3.716 | 4.872 | 6.685 | 11.888 | 14.108 |
| 14 | -0.463 | 0.423 | 2.546 | 2.742 | 3.835 | 5.014 | 6.810 | 11.625 | 13.544 |
| 16 | -0.401 | 0.415 | 2.620 | 2.826 | 3.965 | 5.169 | 6.955 | 11.483 | 13.192 |
| 18 | -0.343 | 0.408 | 2.703 | 2.920 | 4.107 | 5.339 | 7.125 | 11.438 | 12.995 |
| 20 | -0.288 | 0.400 | 2.794 | 3.022 | 4.259 | 5.523 | 7.314 | 11.462 | 12.905 |
| 25 | -0.161 | 0.384 | 3.032 | 3.290 | 4.659 | 6.002 | 7.816 | 11.676 | 12.924 |
| 30 | -0.046 | 0.369 | 3.264 | 3.552 | 5.045 | 6.459 | 8.292 | 11.946 | 13.067 |
| 35 | 0.059 | 0.355 | 3.478 | 3.794 | 5.400 | 6.870 | 8.711 | 12.193 | 13.218 |
| 40 | 0.158 | 0.342 | 3.671 | 4.013 | 5.718 | 7.231 | 9.067 | 12.393 | 13.340 |
| 45 | 0.250 | 0.330 | 3.843 | 4.210 | 5.999 | 7.539 | 9.360 | 12.538 | 13.420 |
| 50 | 0.337 | 0.318 | 3.996 | 4.383 | 6.241 | 7.797 | 9.591 | 12.627 | 13.451 |
| 55 | 0.420 | 0.307 | 4.129 | 4.535 | 6.446 | 8.006 | 9.765 | 12.663 | 13.434 |
| 60 | 0.499 | 0.297 | 4.243 | 4.665 | 6.613 | 8.166 | 9.882 | 12.642 | 13.364 |
| 65 | 0.575 | 0.287 | 4.336 | 4.771 | 6.741 | 8.274 | 9.940 | 12.563 | 13.240 |
| 70 | 0.648 | 0.277 | 4.411 | 4.854 | 6.831 | 8.337 | 9.946 | 12.433 | 13.066 |
| 75 | 0.719 | 0.268 | 4.473 | 4.923 | 6.895 | 8.367 | 9.916 | 12.272 | 12.865 |
| 80 | 0.787 | 0.259 | 4.531 | 4.985 | 6.943 | 8.378 | 9.867 | 12.098 | 12.655 |
| 85 | 0.853 | 0.250 | 4.589 | 5.046 | 6.985 | 8.381 | 9.812 | 11.929 | 12.452 |
|  |  |  |  |  |  |  |  |  |  |
