# Supplementary material for: Dual energy X-ray absorptiometry body composition reference values of limbs and trunk from NHANES 1999–2004 with additional visualization methods
Source: PLoS One. 2017 Mar 27;12(3):e0174180. doi: 10.1371/journal.pone.0174180 (PMC5367711; doi:10.1371/journal.pone.0174180)
Supplement: S47 Table — This table provides L, M, and S values to derive total body LMI Z-scores for 3rd through 97th percentiles for white females ages 8–85. (DOCX) [file pone.0174180.s055.docx]

Table S47: LMS Curve Fit Data providing L, M, and S values for 3^rd^ through 97^th^ percentiles for White Females Ages 8-85 for Total Body LMI.

|  | Females | | | | | | | | |
| --- | --- | --- | --- | --- | --- | --- | --- | --- | --- |
|  |  |  | M | | | | | | |
| Age | L | S | 3 | 5 | 25 | 50 | 75 | 95 | 97 |
| 8 | -0.820 | 0.124 | 10.121 | 10.376 | 11.560 | 12.535 | 13.672 | 15.679 | 16.250 |
| 10 | -0.820 | 0.124 | 10.838 | 11.111 | 12.378 | 13.422 | 14.639 | 16.789 | 17.400 |
| 12 | -0.820 | 0.124 | 11.395 | 11.682 | 13.014 | 14.113 | 15.392 | 17.652 | 18.295 |
| 14 | -0.820 | 0.124 | 11.823 | 12.121 | 13.503 | 14.643 | 15.971 | 18.316 | 18.983 |
| 16 | -0.820 | 0.124 | 12.147 | 12.453 | 13.873 | 15.043 | 16.407 | 18.817 | 19.502 |
| 18 | -0.820 | 0.124 | 12.390 | 12.702 | 14.150 | 15.344 | 16.736 | 19.193 | 19.892 |
| 20 | -0.820 | 0.124 | 12.571 | 12.887 | 14.357 | 15.569 | 16.980 | 19.474 | 20.183 |
| 25 | -0.820 | 0.124 | 12.842 | 13.166 | 14.667 | 15.905 | 17.347 | 19.894 | 20.619 |
| 30 | -0.820 | 0.124 | 12.966 | 13.293 | 14.809 | 16.059 | 17.515 | 20.086 | 20.818 |
| 35 | -0.820 | 0.124 | 13.014 | 13.341 | 14.863 | 16.117 | 17.579 | 20.160 | 20.894 |
| 40 | -0.820 | 0.124 | 13.016 | 13.344 | 14.866 | 16.120 | 17.582 | 20.164 | 20.898 |
| 45 | -0.820 | 0.124 | 12.986 | 13.313 | 14.831 | 16.083 | 17.541 | 20.117 | 20.850 |
| 50 | -0.820 | 0.124 | 12.931 | 13.257 | 14.769 | 16.015 | 17.467 | 20.032 | 20.761 |
| 55 | -0.820 | 0.124 | 12.859 | 13.182 | 14.686 | 15.925 | 17.369 | 19.919 | 20.645 |
| 60 | -0.820 | 0.124 | 12.775 | 13.097 | 14.590 | 15.822 | 17.256 | 19.790 | 20.511 |
| 65 | -0.820 | 0.124 | 12.686 | 13.006 | 14.489 | 15.712 | 17.136 | 19.652 | 20.368 |
| 70 | -0.820 | 0.124 | 12.595 | 12.912 | 14.385 | 15.599 | 17.013 | 19.512 | 20.222 |
| 75 | -0.820 | 0.124 | 12.505 | 12.820 | 14.282 | 15.488 | 16.892 | 19.372 | 20.078 |
| 80 | -0.820 | 0.124 | 12.418 | 12.730 | 14.182 | 15.379 | 16.773 | 19.236 | 19.937 |
| 85 | -0.820 | 0.124 | 12.334 | 12.645 | 14.087 | 15.276 | 16.661 | 19.107 | 19.803 |
|  |  |  |  |  |  |  |  |  |  |
