# Supplementary material for: Dual energy X-ray absorptiometry body composition reference values of limbs and trunk from NHANES 1999–2004 with additional visualization methods
Source: PLoS One. 2017 Mar 27;12(3):e0174180. doi: 10.1371/journal.pone.0174180 (PMC5367711; doi:10.1371/journal.pone.0174180)
Supplement: S48 Table — This table provides L, M, and S values to derive total body LMI Z-scores for 3rd through 97th percentiles for white males ages 8–85. (DOCX) [file pone.0174180.s056.docx]

Table S48: LMS Curve Fit Data providing L, M, and S values for 3^rd^ through 97^th^ percentiles for White Males Ages 8-85 for Total Body LMI.

|  | Males | | | | | | | | |
| --- | --- | --- | --- | --- | --- | --- | --- | --- | --- |
|  |  |  | M | | | | | | |
| Age | L | S | 3 | 5 | 25 | 50 | 75 | 95 | 97 |
| 8 | -0.738 | 0.127 | 10.119 | 10.383 | 11.604 | 12.608 | 13.773 | 15.817 | 16.394 |
| 10 | -0.540 | 0.124 | 10.904 | 11.194 | 12.523 | 13.593 | 14.811 | 16.886 | 17.458 |
| 12 | -0.378 | 0.122 | 11.931 | 12.254 | 13.718 | 14.879 | 16.180 | 18.346 | 18.932 |
| 14 | -0.241 | 0.121 | 13.105 | 13.465 | 15.082 | 16.347 | 17.747 | 20.036 | 20.647 |
| 16 | -0.123 | 0.119 | 14.104 | 14.496 | 16.244 | 17.596 | 19.076 | 21.460 | 22.089 |
| 18 | -0.018 | 0.118 | 14.783 | 15.199 | 17.038 | 18.446 | 19.973 | 22.402 | 23.037 |
| 20 | 0.075 | 0.117 | 15.193 | 15.625 | 17.521 | 18.959 | 20.507 | 22.942 | 23.573 |
| 25 | 0.274 | 0.114 | 15.593 | 16.044 | 18.002 | 19.460 | 21.001 | 23.375 | 23.980 |
| 30 | 0.435 | 0.112 | 15.727 | 16.190 | 18.173 | 19.626 | 21.143 | 23.441 | 24.019 |
| 35 | 0.572 | 0.111 | 15.842 | 16.314 | 18.319 | 19.768 | 21.264 | 23.501 | 24.059 |
| 40 | 0.691 | 0.109 | 15.936 | 16.416 | 18.437 | 19.882 | 21.359 | 23.545 | 24.086 |
| 45 | 0.795 | 0.108 | 15.978 | 16.463 | 18.493 | 19.929 | 21.387 | 23.524 | 24.049 |
| 50 | 0.889 | 0.107 | 15.953 | 16.442 | 18.471 | 19.894 | 21.329 | 23.415 | 23.925 |
| 55 | 0.974 | 0.106 | 15.872 | 16.362 | 18.383 | 19.789 | 21.197 | 23.231 | 23.726 |
| 60 | 1.051 | 0.105 | 15.738 | 16.227 | 18.232 | 19.617 | 20.997 | 22.977 | 23.457 |
| 65 | 1.122 | 0.104 | 15.541 | 16.027 | 18.008 | 19.367 | 20.714 | 22.637 | 23.102 |
| 70 | 1.188 | 0.103 | 15.289 | 15.769 | 17.718 | 19.047 | 20.359 | 22.222 | 22.670 |
| 75 | 1.249 | 0.102 | 15.004 | 15.477 | 17.390 | 18.687 | 19.962 | 21.765 | 22.197 |
| 80 | 1.306 | 0.101 | 14.709 | 15.175 | 17.050 | 18.315 | 19.554 | 21.297 | 21.714 |
| 85 | 1.360 | 0.101 | 14.423 | 14.882 | 16.720 | 17.954 | 19.158 | 20.847 | 21.250 |
|  |  |  |  |  |  |  |  |  |  |
