# Supplementary material for: Dual energy X-ray absorptiometry body composition reference values of limbs and trunk from NHANES 1999–2004 with additional visualization methods
Source: PLoS One. 2017 Mar 27;12(3):e0174180. doi: 10.1371/journal.pone.0174180 (PMC5367711; doi:10.1371/journal.pone.0174180)
Supplement: S1 File — (PDF) [file pone.0174180.s057.pdf]

Individual FMI/LMI Chart

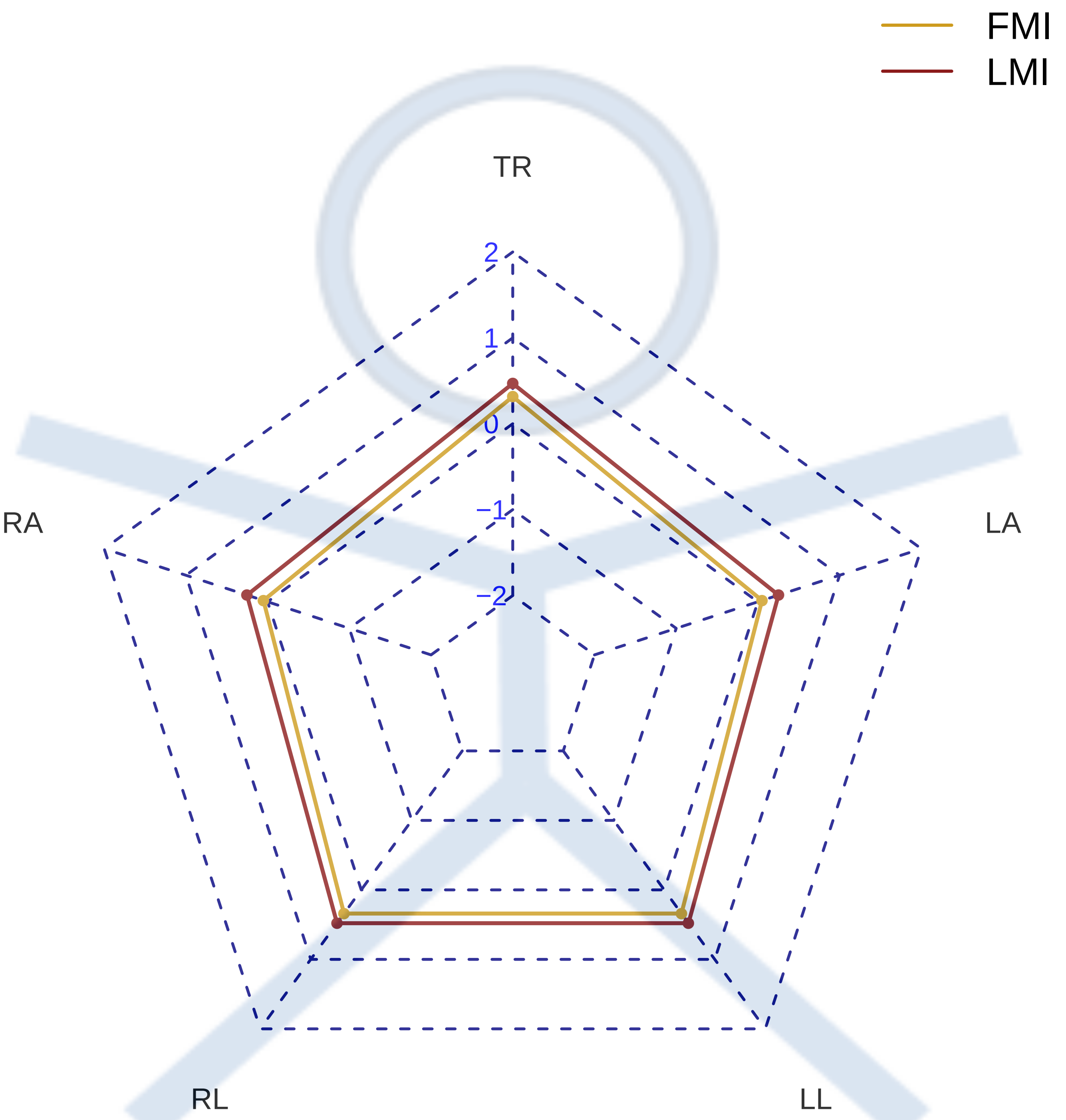

Radar chart of a 20 year old Hispanic female that is 160 cm tall.

Trunk Fat Mass/FMI: 12.0/4.7   Trunk Lean Mass/LMI: 21.0/8.2.

Right/Left Arm FMI: 0.5/0.5   Right/Left Arm LMI: 0.9/0.9.

Right/Left Arm Fat Mass: 1.4/1.4   Right/Left Arm Lean Mass: 2.2/2.2.

Right/Left Leg FMI: 2.0/2.0   Right/Left Leg LMI: 2.7/2.7.

Right/Left Leg Fat Mass: 5.0/5.0   Right/Left Leg Lean Mass: 7.0/7.0.
